# Genetic loci and prioritization of genes for kidney function decline derived from a meta-analysis of 62 longitudinal genome-wide association studies

**DOI:** 10.1016/j.kint.2022.05.021

**Published:** 2022-06-16

**Authors:** Mathias Gorski, Humaira Rasheed, Alexander Teumer, Laurent F. Thomas, Sarah E. Graham, Gardar Sveinbjornsson, Thomas W. Winkler, Felix Günther, Klaus J. Stark, Jin-Fang Chai, Bamidele O. Tayo, Matthias Wuttke, Yong Li, Adrienne Tin, Tarunveer S. Ahluwalia, Johan Ärnlöv, Bjørn Olav Åsvold, Stephan J. L. Bakker, Bernhard Banas, Nisha Bansal, Mary L. Biggs, Ginevra Biino, Michael Böhnke, Eric Boerwinkle, Erwin P. Bottinger, Hermann Brenner, Ben Brumpton, Robert J. Carroll, Layal Chaker, John Chalmers, Miao-Li Chee, Miao-Ling Chee, Ching-Yu Cheng, Audrey Y. Chu, Marina Ciullo, Massimiliano Cocca, James P. Cook, Josef Coresh, Daniele Cusi, Martin H. de Borst, Frauke Degenhardt, Kai-Uwe Eckardt, Karlhans Endlich, Michele K. Evans, Mary F Feitosa, Andre Franke, Sandra Freitag-Wolf, Christian Fuchsberger, Piyush Gampawar, Ron T. Gansevoort, Mohsen Ghanbari, Sahar Ghasemi, Vilmantas Giedraitis, Christian Gieger, Daniel F Gudbjartsson, Stein Hallan, Pavel Hamet, Asahi Hishida, Kevin Ho, Edith Hofer, Bernd Holleczek, Hilma Holm, Anselm Hoppmann, Katrin Horn, Nina Hutri-Kähönen, Kristian Hveem, Shih-Jen Hwang, M. Arfan Ikram, Navya Shilpa Josyula, Bettina Jung, Mika Kähönen, Irma Karabegović, Chiea-Chuen Khor, Wolfgang Koenig, Holly Kramer, Bernhard K. Krämer, Brigitte Kühnel, Johanna Kuusisto, Markku Laakso, Leslie A. Lange, Terho Lehtimäki, Man Li, Wolfgang Lieb, Lifelines cohort study, Lars Lind, Cecilia M. Lindgren, Ruth J. F. Loos, Mary Ann Lukas, Leo-Pekka Lyytikäinen, Anubha Mahajan, Pamela R. Matias-Garcia, Christa Meisinger, Thomas Meitinger, Olle Melander, Yuri Milaneschi, Pashupati P. Mishra, Nina Mononen, Andrew P. Morris, Josyf C. Mychaleckyj, Girish N. Nadkarni, Mariko Naito, Masahiro Nakatochi, Mike A. Nalls, Matthias Nauck, Kjell Nikus, Boting Ning, Ilja M. Nolte, Teresa Nutile, Michelle L. O’Donoghue, Jeffrey O’Connell, Isleifur Olafsson, Marju Orho-Melander, Afshin Parsa, Sarah A. Pendergrass, Brenda W. J. H. Penninx, Mario Pirastu, Michael H. Preuss, Bruce M. Psaty, Laura M. Raffield, Olli T. Raitakari, Myriam Rheinberger, Kenneth M. Rice, Federica Rizzi, Alexander R. Rosenkranz, Peter Rossing, Jerome I. Rotter, Daniela Ruggiero, Kathleen A. Ryan, Charumathi Sabanayagam, Erika Salvi, Helena Schmidt, Reinhold Schmidt, Markus Scholz, Ben Schöttker, Christina-Alexandra Schulz, Sanaz Sedaghat, Christian M. Shaffer, Karsten B. Sieber, Xueling Sim, Mario Sims, Harold Snieder, Kira J. Stanzick, Unnur Thorsteinsdottir, Hannah Stocker, Konstantin Strauch, Heather M. Stringham, Patrick Sulem, Silke Szymczak, Kent D. Taylor, Chris H. L. Thio, Johanne Tremblay, Simona Vaccargiu, Pim van der Harst, Peter J. van der Most, Niek Verweij, Uwe Völker, Kenji Wakai, Melanie Waldenberger, Lars Wallentin, Stefan Wallner, Judy Wang, Dawn M. Waterworth, Harvey D. White, Cristen J. Willer, Tien-Yin Wong, Mark Woodward, Qiong Yang, Laura M. Yerges-Armstrong, Martina Zimmermann, Alan B. Zonderman, Tobias Bergler, Kari Stefansson, Carsten A. Böger, Cristian Pattaro, Anna Köttgen, Florian Kronenberg, Iris M. Heid

**Affiliations:** 1 Department of Genetic Epidemiology, University of Regensburg, Regensburg, Germany.; 2 Department of Nephrology, University Hospital Regensburg, Regensburg, Germany.; 3 K. G. Jebsen Center for Genetic Epidemiology, Department of Public Health and Nursing, Faculty of Medicine and Health Sciences, NTNU, Norwegian University of Science and Technology, Trondheim, Norway; 4 MRC Integrative Epidemiology Unit, Population Health Sciences, Bristol Medical School, University of Bristol, Bristol, United Kingdom; 5 Institute for Community Medicine, University Medicine Greifswald, Greifswald, Germany.; 6 DZHK (German Center for Cardiovascular Research), partner site Greifswald, Greifswald, Germany.; 7 Department of Population Medicine and Lifestyle Diseases Prevention, Medical University of Bialystok, Bialystok, Poland; 8 Department of Clinical and Molecular Medicine, NTNU, Norwegian University of Science and Technology, Trondheim, Norway; 9 BioCore - Bioinformatics Core Facility, Norwegian University of Science and Technology, Trondheim. Norway; 10 Department of Internal Medicine, Division of Cardiology, University of Michigan, Ann Arbor, MI 48109, USA; 11 deCODE Genetics/Amgen, Inc., Reykjavik, Iceland.; 12 Statistical Consulting Unit StaBLab, Department of Statistics, LMU Munich, Munich, Germany.; 13 Saw Swee Hock School of Public Health, National University of Singapore and National University Health System, Singapore, Singapore.; 14 Department of Public Health Sciences, Loyola University Chicago, Maywood, IL, USA.; 15 Institute of Genetic Epidemiology, Department of Biometry, Epidemiology and Medical Bioinformatics, Faculty of Medicine and Medical Center–University of Freiburg, Freiburg, Germany.; 16 Renal Division, Department of Medicine IV, Faculty of Medicine and Medical Center–University of Freiburg, Freiburg, Germany.; 17 Memory Impairment and Neurodegenerative Dementia (MIND) Center, University of Mississippi Medical Center, Jackson, MS, USA.; 18 Division of Nephrology, Department of Medicine, University of Mississippi Medical Center, Jackson, MS, USA.; 19 Steno Diabetes Center Copenhagen, Gentofte, Denmark.; 20 The Bioinformatics Center, Department of Biology, University of Copenhagen, Copenhagen, Denmark.; 21 Division of Family Medicine and Primary Care, Department of Neurobiology, Care Sciences and Society, Karolinska Institutet, Stockholm, Sweden.; 22 School of Health and Social Studies, Dalarna University, Stockholm, Sweden.; 23 Department of Endocrinology, Clinic of Medicine, St. Olavs Hospital, Trondheim University Hospital, Trondheim, Norway; 24 Division of Nephrology, Department of Internal Medicine, University of Groningen, University Medical Center Groningen, Groningen, the Netherlands.; 25 Division of Nephrology, University of Washington, Seattle, WA, USA.; 26 Kidney Research Institute, University of Washington, Seattle, WA, USA.; 27 Cardiovascular Health Research Unit, Department of Medicine, University of Washington, Seattle, WA, USA.; 28 Department of Biostatistics, University of Washington, Seattle, WA, USA.; 29 Institute of Molecular Genetics, National Research Council of Italy, Pavia, Italy.; 30 Department of Biostatistics and Center for Statistical Genetics, University of Michigan, Ann Arbor, MI, USA.; 31 Human Genetics Center, University of Texas Health Science Center, Houston, TX, USA.; 32 Charles Bronfman Institute for Personalized Medicine, Icahn School of Medicine at Mount Sinai, New York, NY, USA.; 33 Digital Health Center, Hasso Plattner Institute and University of Potsdam, Potsdam, Germany.; 34 Division of Clinical Epidemiology and Aging Research, German Cancer Research Center (DKFZ), Heidelberg, Germany.; 35 Network Aging Research, Heidelberg University, Heidelberg, Germany.; 36 Clinic of Thoracic and Occupational Medicine, St. Olavs Hospital, Trondheim University Hospital, Trondheim, Norway; 37 Department of Biomedical Informatics, Vanderbilt University Medical Center, Nashville, TN, USA.; 38 Department of Epidemiology, Erasmus MC, University Medical Center Rotterdam, Rotterdam, the Netherlands.; 39 Department of Internal Medicine, Erasmus MC, University Medical Center Rotterdam, Rotterdam, the Netherlands.; 40 The George Institute for Global Health, University of New South Wales, Sydney, Australia.; 41 Singapore Eye Research Institute, Singapore National Eye Center, Singapore, Singapore.; 42 Ophthalmology and Visual Sciences Academic Clinical Program (Eye ACP), Duke - NUS Medical School, Singapore, Singapore.; 43 Department of Ophthalmology, Yong Loo Lin School of Medicine, National University of Singapore and National University Health System, Singapore, Singapore.; 44 Genetics, Merck & Co., Inc, Kenilworth, NJ, USA.; 45 Institute of Genetics and Biophysics ‘Adriano Buzzati-Traverso’-CNR, Naples, Italy.; 46 IRCCS Neuromed, Pozzilli, Italy.; 47 Institute for Maternal and Child Health, IRCCS ‘Burlo Garofolo’, Trieste, Italy.; 48 Department of Health Data Science, University of Liverpool, Liverpool, UK.; 49 Department of Epidemiology, Johns Hopkins Bloomberg School of Public Health, Baltimore, MD, USA.; 50 Institute of Biomedical Technologies, National Research Council of Italy, Milan, Italy.; 51 Bio4Dreams–Business Nursery for Life Sciences, Milan, Italy.; 52 Institute of Clinical Molecular Biology, Christian-Albrechts-University of Kiel, Kiel, Germany.; 53 Department of Nephrology and Medical Intensive Care, Charité - Universitätsmedizin Berlin, Berlin, Germany; 54 Department of Nephrology and Hypertension, Friedrich Alexander University Erlangen-Nürnberg (FAU), Erlangen, Germany.; 55 Department of Anatomy and Cell Biology, University Medicine Greifswald, Greifswald, Germany.; 56 Laboratory of Epidemiology and Population Sciences, National Institute on Aging, Intramural Research Program, US National Institutes of Health, Baltimore, MD, USA.; 57 Division of Statistical Genomics, Department of Genetics, Washington University School of Medicine, St. Louis, MO, USA.; 58 Institute of Medical Informatics and Statistics, Kiel University, University Hospital Schleswig-Holstein, Kiel, Germany.; 59 Eurac Research, Institute for Biomedicine (affiliated with the University of Lübeck), Bolzano, Italy.; 60 Institute of Molecular Biology and Biochemistry, Center for Molecular Medicine, Medical University of Graz, Graz, Austria.; 61 Department of Genetics, School of Medicine, Mashhad University of Medical Sciences, Mashhad, Iran.; 62 Molecular Geriatrics, Department of Public Health and Caring Sciences, Uppsala University, Uppsala, Sweden.; 63 Research Unit Molecular Epidemiology, Helmholtz Zentrum München–German Research Center for Environmental Health, Neuherberg, Germany.; 64 Institute of Epidemiology, Helmholtz Zentrum München - German Research Center for Environmental Health, Neuherberg, Germany.; 65 German Center for Diabetes Research (DZD), Neuherberg, Germany.; 66 Iceland School of Engineering and Natural Sciences, University of Iceland, Reykjavik, Iceland; 67 Department of Nephrology, St. Olavs Hospital, Trondheim University Hospital, Trondheim, Norway; 68 Montreal University Hospital Research Center, CHUM, Montreal, Quebec, Canada.; 69 Medpharmgene, Montreal, Quebec, Canada.; 70 CRCHUM, Montreal, Canada.; 71 Department of Preventive Medicine, Nagoya University Graduate School of Medicine, Nagoya, Japan.; 72 Kidney Health Research Institute (KHRI), Geisinger, Danville, PA, USA.; 73 Department of Nephrology, Geisinger, Danville, PA, USA.; 74 Clinical Division of Neurogeriatrics, Department of Neurology, Medical University of Graz, Graz, Austria.; 75 Institute for Medical Informatics, Statistics and Documentation, Medical University of Graz, Graz, Austria.; 76 Institute for Medical Informatics, Statistics and Epidemiology, University of Leipzig, Leipzig, Germany.; 77 LIFE Research Center for Civilization Diseases, University of Leipzig, Leipzig, Germany.; 78 Department of Pediatrics, Tampere University Hospital, Tampere, Finland.; 79 Department of Pediatrics, Faculty of Medicine and Health Technology, Tampere University, Tampere, Finland.; 80 NHLBI’s Framingham Heart Study, Framingham, MA, USA.; 81 Population Sciences Branch, Division of Intramural Research, National Heart, Lung, and Blood Institute, National Institutes of Health, Bethesda, MD, USA; 82 Geisinger Research, Biomedical and Translational Informatics Institute, Rockville, MD, USA.; 83 Department of Nephrology and Rheumatology, Kliniken Südostbayern, Traunstein, Germany.; 84 KfH Kidney Center Traunstein; 85 Department of Clinical Physiology, Tampere University Hospital, Tampere, Finland.; 86 Department of Clinical Physiology, Finnish Cardiovascular Research Center - Tampere, Faculty of Medicine and Health Technology, Tampere University, Tampere, Finland.; 87 Genome Institute of Singapore, Agency for Science Technology and Research, Singapore, Singapore.; 88 Deutsches Herzzentrum München, Technische Universität München, Munich, Germany.; 89 DZHK (German Center for Cardiovascular Research), Partner Site Munich Heart Alliance, Munich, Germany.; 90 Institute of Epidemiology and Medical Biometry, University of Ulm, Ulm, Germany.; 91 Division of Nephrology and Hypertension, Loyola University Chicago, Chicago, IL, USA.; 92 Department of Medicine (Nephrology, Hypertensiology, Rheumatology, Endocrinology, Diabetology), Medical Faculty Mannheim, Heidelberg University, Mannheim, Germany.; 93 Department of Medicine, Kuopio University Hospital, Kuopio, Finland.; 94 Centre for Medicine and Clinical Research, University of Eastern Finland School of Medicine, Kuopio, Finland.; 95 Division of Biomedical Informatics and Personalized Medicine, School of Medicine, University of Colorado Denver-Anschutz Medical Campus, Aurora, CO, USA.; 96 Department of Clinical Chemistry, Fimlab Laboratories, Tampere, Finland.; 97 Department of Clinical Chemistry, Finnish Cardiovascular Research Center - Tampere, Faculty of Medicine and Health Technology, Tampere University, Tampere, Finland.; 98 Division of Nephrology and Hypertension, Department of Medicine, University of Utah, Salt Lake City, USA.; 99 Institute of Epidemiology and Biobank Popgen, Kiel University, Kiel, Germany.; 100 A list of members and affiliations appears in the Supplementary Online Material.; 101 Department of Medical Sciences, Uppsala University, Uppsala, Sweden.; 102 Nuffield Department of Population Health, University of Oxford, Oxford, UK.; 103 Broad Institute of Harvard and MIT, Cambridge, MA, USA.; 104 Wellcome Center for Human Genetics, University of Oxford, Oxford, UK.; 105 Nuffield Dept. of Women’s & Reproductive Health, University of Oxford, Level 3, Women’s Centre, John Radcliffe Hospital, Oxford, OX3 9DU.; 106 Li Ka Shing Centre for Health Information and Discovery, The Big Data Institute, University of Oxford, Oxford, UK; 107 The Mindich Child Health and Development Institute, Icahn School of Medicine at Mount Sinai, New York, NY, USA.; 108 Clinical Sciences, GlaxoSmithKline, Albuquerque, NM, USA.; 109 Oxford Center for Diabetes, Endocrinology and Metabolism, University of Oxford, Oxford, UK.; 110 TUM School of Medicine, Technical University of Munich, Munich, Germany.; 111 Independent Research Group Clinical Epidemiology, Helmholtz Zentrum München, German Research Center for Environmental Health, Neuherberg, Germany.; 112 Chair of Epidemiology, University of Augsburg, University Hospital Augsburg; 113 Institute of Human Genetics, Helmholtz Zentrum München, Neuherberg, Germany.; 114 Institute of Human Genetics, Technische Universität München, Munich, Germany.; 115 Hypertension and Cardiovascular Disease, Department of Clincial Sciences Malmö, Lund University, Malmö, Sweden.; 116 Department of Psychiatry, Amsterdam Public Health and Amsterdam Neuroscience, Amsterdam UMC/Vrije Universiteit and GGZ inGeest, Amsterdam, the Netherlands.; 117 Centre for Genetics and Genomics Versus Arthritis, Centre for Musculoskeletal Research, The University of Manchester, Manchester, UK; 118 Center for Public Health Genomics, University of Virginia, Charlottesville, Charlottesville, VA, USA.; 119 Division of Nephrology, Department of Medicine, Icahn School of Medicine at Mount Sinai, New York, NY, USA.; 120 Department of Oral Epidemiology, Graduate School of Biomedical and Health Sciences, Hiroshima University, Hiroshima, Japan; 121 Public Health Informatics Unit, Department of Integrated Health Sciences, Nagoya University Graduate School of Medicine, Nagoya, Japan; 122 Laboratory of Neurogenetics, National Institute on Aging, National Institutes of Health, Bethesda, MD, USA.; 123 Data Tecnica International, Glen Echo, MD, USA.; 124 Institute of Clinical Chemistry and Laboratory Medicine, University Medicine Greifswald, Greifswald, Germany.; 125 Department of Cardiology, Heart Center, Tampere University Hospital, Tampere, Finland.; 126 Department of Cardiology, Finnish Cardiovascular Research Center - Tampere, Faculty of Medicine and Health Technology, Tampere University, Tampere, Finland.; 127 Department of Biostatistics, Boston University School of Public Health, Boston, MA, USA.; 128 Department of Epidemiology, University of Groningen, University Medical Center Groningen, Groningen, the Netherlands.; 129 Cardiovascular Division, Brigham and Women’s Hospital, Boston, MA, USA.; 130 TIMI Study Group, Boston, MA, USA.; 131 University of Maryland School of Medicine, Baltimore, MD, USA.; 132 Department of Clinical Biochemistry, Landspitali University Hospital, Reykjavik, Iceland.; 133 Diabetes and Cardiovascular Disease–Genetic Epidemiology, Department of Clincial Sciences in Malmö, Lund University, Malmö, Sweden.; 134 Division of Kidney, Urologic and Hematologic Diseases, National Institute of Diabetes and Digestive and Kidney Diseases, National Institutes of Health, Bethesda, MD, USA.; 135 Department of Medicine, University of Maryland School of Medicine, Baltimore, MD, USA.; 136 Geisinger Research, Biomedical and Translational Informatics Institute, Danville, PA, USA.; 137 Institute of Genetic and Biomedical Research, National Research Council of Italy, UOS of Sassari, Li Punti, Sassari, Italy.; 138 Cardiovascular Health Research Unit, Department of Medicine, Department of Epidemiology, Department of Health Services, University of Washington, Seattle, WA, USA.; 139 Department of Genetics, University of North Carolina, Chapel Hill, NC, USA.; 140 Centre for Population Health Research, University of Turku and Turku University Hospital, Turku, Finland.; 141 Department of Clinical Physiology and Nuclear Medicine, Turku University Hospital, Turku, Finland.; 142 Research Center of Applied and Preventive Cardiovascular Medicine, University of Turku, Turku, Finland.; 143 Department of Health Sciences, University of Milan, Milano, Italy.; 144 ePhood Scientific Unit, ePhood SRL, Milano, Italy.; 145 Department of Internal Medicine, Division of Nephrology, Medical University Graz, Graz, Austria.; 146 Department of Clinical Medicine, University of Copenhagen, Copenhagen, Denmark; 147 The Institute for Translational Genomics and Population Sciences, Department of Pediatrics, The Lundquist Institute for Biomedical Innovation at Harbor-UCLA Medical Center, Torrance, CA USA.; 148 Division of Endocrinology, Diabetes and Nutrition, University of Maryland School of Medicine, Baltimore, MD, USA.; 149 Neuroalgology Unit, Fondazione IRCCS Istituto Neurologico ‘Carlo Besta’, Milan, Italy.; 150 Department of Preventive Medicine, Northwestern University, Feinberg School of Medicine, Chicago, Illinois, USA.; 151 Human Genetics, GlaxoSmithKline, Collegeville, Pennsylvania, USA.; 152 Department of Medicine, University of Mississippi Medical Center, Jackson, MS, USA.; 153 Faculty of Medicine, School of Health Sciences, University of Iceland, Reykjavik, Iceland.; 154 Institute of Genetic Epidemiology, Helmholtz Zentrum München - German Research Center for Environmental Health, Neuherberg, Germany.; 155 Chair of Genetic Epidemiology, IBE, Faculty of Medicine, Ludwig-Maximilians-Universität München, München, Germany.; 156 Institute of Medical Biostatistics, Epidemiology and Informatics (IMBEI), University Medical Center, Johannes Gutenberg University, Mainz, Germany; 157 Institute of Medical Biometry and Statistics, University of Lübeck, University Hospital Schleswig-Holstein, Campus Lübeck, Lübeck, Germany; 158 Department of Cardiology, University of Groningen, University Medical Center Groningen, Groningen, the Netherlands.; 159 Department of Genetics, University of Groningen, University Medical Center Groningen, Groningen, the Netherlands.; 160 Durrer Center for Cardiovascular Research, The Netherlands Heart Institute, Utrecht, the Netherlands.; 161 Interfaculty Institute for Genetics and Functional Genomics, University Medicine Greifswald, Greifswald, Germany.; 162 Cardiology, Department of Medical Sciences, Uppsala University, Uppsala, Sweden.; 163 Uppsala Clinical Research Center, Uppsala University, Uppsala, Sweden.; 164 Institute of Clinical Chemistry and Laboratory Medicine, University Hospital Regensburg, Regensburg, Germany.; 165 Green Lane Cardiovascular Service, Auckland City Hospital and University of Auckland, Auckland, New Zealand.; 166 Department of Computational Medicine and Bioinformatics, University of Michigan, Ann Arbor, MI 48109, USA; 167 Department of Human Genetics, University of Michigan, Ann Arbor, MI 48109, USA; 168 The George Institute for Global Health, University of Oxford, Oxford, UK.; 169 Institute of Genetic Epidemiology, Department of Genetics and Pharmacology, Medical University of Innsbruck, Innsbruck, Austria.; 170 These authors contributed equally: Mathias Gorski, Humaira Rasheed and Alexander Teumer.; 171 These authors jointly supervised this work: Cristian Pattaro, Anna Köttgen, Florian Kronenberg and Iris M. Heid.

**Keywords:** acute kidney injury, diabetes, chronic kidney disease, gene expression

## Abstract

Estimated glomerular filtration rate (eGFR) reflects kidney function. Progressive eGFR-decline can lead to kidney failure, necessitating dialysis or transplantation. Hundreds of loci from genome-wide association studies (GWAS) for eGFR help explain population cross section variability. Since the contribution of these or other loci to eGFR-decline remains largely unknown, we derived GWAS for annual eGFR-decline and meta-analyzed 62 longitudinal studies with eGFR assessed twice over time in all 343,339 individuals and in high-risk groups. We also explored different covariate adjustment. Twelve genome-wide significant independent variants for eGFR-decline unadjusted or adjusted for eGFR-baseline (11 novel, one known for this phenotype), including nine variants robustly associated across models were identified. All loci for eGFR-decline were known for cross-sectional eGFR and thus distinguished a subgroup of eGFR loci. Seven of the nine variants showed variant-by-age interaction on eGFR cross section (further about 350,000 individuals), which linked genetic associations for eGFR-decline with age-dependency of genetic cross-section associations. Clinically important were two to four-fold greater genetic effects on eGFR-decline in high-risk subgroups. Five variants associated also with chronic kidney disease progression mapped to genes with functional *in-silico* evidence (*UMOD, SPATA7, GALNTL5, TPPP*). An unfavorable versus favorable nine-variant genetic profile showed increased risk odds ratios of 1.35 for kidney failure (95% confidence intervals 1.03–1.77) and 1.27 for acute kidney injury (95% confidence intervals 1.08–1.50) in over 2000 cases each, with matched controls). Thus, we provide a large data resource, genetic loci, and prioritized genes for kidney function decline, which help inform drug development pipelines revealing important insights into the age-dependency of kidney function genetics.

## INTRODUCTION

Glomerular filtration rate (GFR) is accepted as best overall index of kidney function^1^. A GFR<60 mL/min/1.73m^2^ defines chronic kidney disease (CKD)^2^, which affects about 10% of adults^3^. A decline in GFR over time is characteristic for CKD-progression, which can lead to kidney failure^4^ requiring dialysis or kidney transplantation with a high risk of premature mortality^5^. In population studies on kidney function, estimated GFR (eGFR) is usually derived from serum creatinine^6^ and annual eGFR-decline as the difference between two such assessments divided by the years between these assessments. Decline in eGFR is age-related, with a physiological loss of ~1 mL/min/1.73m^2^ per year^2^ generally and 3 mL/min/1.73m^2^ per year in the presence of diabetes mellitus (DM), a major risk factor for CKD-progression^7,8^. Therapeutic options to decelerate kidney function decline are limited. In addition to pharmacological inhibitors of the RAAS-system^9^, the recent introduction SGLT2 inhibitors show promising reno-protective effects^10,11^. An understanding of the mechanisms of kidney function decline and the developing of new therapeutic options is thus of high clinical and public health relevance^7,12^.

Genes underneath genome-wide association study (GWAS) loci for diseases and biomarkers help identify new therapies^13^. Open access GWAS summary statistics from large sample sizes are a highly queried resource, also for causal inference studies^14^. Hundreds of loci and genes are identified by cross-sectional GWAS for eGFR, i.e. GWAS for eGFR based on a single serum creatinine measurement^15–18^, which help explain population variability. However, the mechanisms underlying a genetic variant association with lower but stable eGFR over time might not always be disease-relevant. GWAS on parameters more directly linked to disease progression are thought to better inform drug development^19^.

Current evidence from GWAS on annual eGFR-decline is limited, owed to substantial logistics in conducting longitudinal studies and thus small sample sizes. Only one variant, in the *UMOD-PDILT* locus, has been identified at genome-wide significance^20^ (n~60,000). With an estimated heritability of 38% for annual eGFR-decline^20^, comparable to 33%−39% estimated for cross-sectional eGFR in general populations^21,15^, much more can be expected in larger sample sizes. Further three loci were genome-wide significant in an extreme phenotype approach, comparing individuals with large eGFR-decline or steep drop into CKD with respective controls^22^. While these are important binary clinical endpoints, methodological literature supports the use of regression methods on undichotomized variables^23^.

The limited availability of longitudinal GWAS is not only an issue for kidney function decline, but also generally: e.g. change in lung function (n=27,249^24^), glucose (n=13,807^25^), or blood pressure (n=33,720^26^); consequently, locus findings on biomarker change are few and often unstable^14^. A challenge beyond power is limited experience in longitudinal GWAS with regard to covariate adjustment: clinical trials for disease-related biomarker change require control for differences in baseline levels between therapy groups^27^. However, covariate adjustment in GWAS requires a careful choice^28^: it can reveal important mediator effects (e.g. DM adjusted for BMI^29^), alter the phenotype (e.g. waist-to-hip ratio “unexpected” by body-mass-index^28,30^), yield artefacts from heritable covariates (collider bias^28^) or non-sense association (e.g. sex adjusted for height^31^). The impact of covariate adjustment on longitudinal GWAS on eGFR-decline, and biomarker change generally, is not well explored.

We thus aimed to identify genetic loci associated with annual eGFR-decline and CKD-progression (defined as eGFR-decline among individuals with CKD at baseline) and to prioritize genes that may inform drug development for slowing down eGFR-decline and CKD-progression. We also aimed to fill the gap of large-data genome-wide SNP summary statistics for annual eGFR-decline and CKD-progression, to help future meta-analyses and Mendelian randomization studies. Finally, we wanted to understand the impact of different covariate adjustment and whether a SNP associated with eGFR-decline showed an age-dependent association on eGFR cross-sectionally (i.e. SNP-by-age interaction on eGFR cross-sectionally). By this, we aimed to contribute to a better understanding of the interpretation of genetic findings for eGFR-decline and other progression traits.

To achieve these aims, we (i) increased sample size for GWAS on annual eGFR-decline to >340,000 individuals based on the CKDGen consortium^32^ and UK Biobank^33^, (ii) applied a suite of covariate adjustment models, (iii) analyzed SNP-by-age interaction on eGFR cross-sectionally in >350,000 individuals independent of the GWAS on decline, and (v) conducted genetic risk score (GRS) analyses for acute kidney injury (AKI) and end-stagekidney disease (ESKD).

## METHODS

We conducted GWAS meta-analysis based on study-specific summary statistics. Each study utilized data on two measurements of serum creatinine over time and genome-wide SNP-information imputed to 1000 Genomes^34^ phase 1 or phase 3, the Haplotype Reference Consortium^35^ v1.1 or similar (Table S1&S2). Serum creatinine measured at baseline and follow-up were used to estimate eGFR at baseline and follow-up, respectively, according to the Chronic Kidney Disease Epidemiology Collaboration (CKD-EPI) equation^6^. Annual eGFR-decline was defined as “-(eGFR at follow-up - eGFR at baseline) / number of years of follow-up”. GWAS analyses were conducted separately by ancestry (if applicable), where ancestry was defined by genetic principal components or participants’ self-report. GWAS were based on linear regression with different covariate adjustment conducted overall and focused on individuals with DM or CKD at baseline.

Study-specific genome-wide summary statistics and detailed phenotype information were transferred to the meta-analysis center. For each SNP, summary statistics were pooled and genomic control corrected. Significant genetic variants were identified and respective locus regions selected.

Additionally, we investigated identified SNPs for SNP-by-age interaction on cross-sectional eGFR (based on creatinine or cystatin C, eGFRcrea, eGFRcys) using UK Biobank data that was independent of the SNP identification step (excluding the individuals in the decline GWAS). We computed the GRS and its association on eGFR-decline in the HUNT study via linear regression and provided odds ratios (OR) for GRS association in case-control studies on AKI and ESKD via logistic regression.

Detailed methods are provided in the Supplementary Methods.

## RESULTS

### Overview across studies and models for GWAS

This GWAS meta-analysis included 343,339 individuals from 62 studies (Supplementary Table S1&S3, Supplementary Figure S1, Methods) and 12,403,901 analyzable SNPs. Most studies were population-based (76%) and of European ancestry (74%). Study-specific median annual eGFR-decline was independent of sample size and follow-up length (Supplementary Figure S2A&S2B) and the median across studies was 1.32 mL/min/1.73m^2^ per year; follow-up length was 1–21 years (median [25^th^, 75th] = 5 years [4,7]); median age ranged from 33 to 77 years (Supplementary Figure S2C).

All analyses were adjusted for age-, sex, and study-specific covariates, which is not mentioned further from here on (stable across different modes of age-adjustment, Supplementary Figure S3). We had five GWAS results for eGFR-decline (Methods): (i) “unadjusted”, (ii) “DM-adjusted”, (iii) “adjusted for eGFR-baseline”, (iv) restricted to individuals with DM at baseline (unadjusted), and (v) restricted to individuals with CKD at baseline (unadjusted).

### Similarities and differences across different model adjustments

There is, to date, no standard conduct for GWAS on eGFR-decline with regard to covariate adjustment. We explored the impact of two potentially important covariates additional to age and sex: (i) DM, as an important risk factors for eGFR-decline and potential mediator, and (ii) eGFR at baseline, as adjustment for baseline levels in analyses of change over time has noted pros (larger effects, better detectability) and cons (biased effects)^36,37^.

With regard to DM-adjustment, this model was computed in all studies (n=343,339; 62 studies) and compared to unadjusted results for a subset of studies of varying scope (n=103,970). DM-adjusted SNP-associations on eGFR-decline were precisely the same as unadjusted, in terms of beta-estimates and standard errors (Supplementary Figure S4A, Supplementary Note S1). We therefore did not distinguish these two models further.

In contrast, adjustment for eGFR-baseline altered SNP-associations on eGFR-decline (Supplementary Figure S4B). Therefore, results from both eGFR-decline unadjusted and adjusted for eGFR-baseline were evaluated in the following. GWAS summary statistics for eGFR-decline adjusted for eGFR-baseline were formula-derived from GWAS summary statistics for unadjusted eGFR-decline and for eGFR-baseline together with study-specific phenotypic information (Supplementary Note S2). In a subset of studies (n=103,970), we validated that the formula-approach worked very well in our setting (Supplementary Note S3, Supplementary Figure S4C&D). Meta-analysis yielded GWAS results for eGFR-decline adjusted for eGFR-baseline for 320,737 individuals (50 studies, Supplementary Figure S1).

### Twelve variants identified for eGFR-decline unadjusted or adjusted for eGFR-baseline

**Figure 1:**
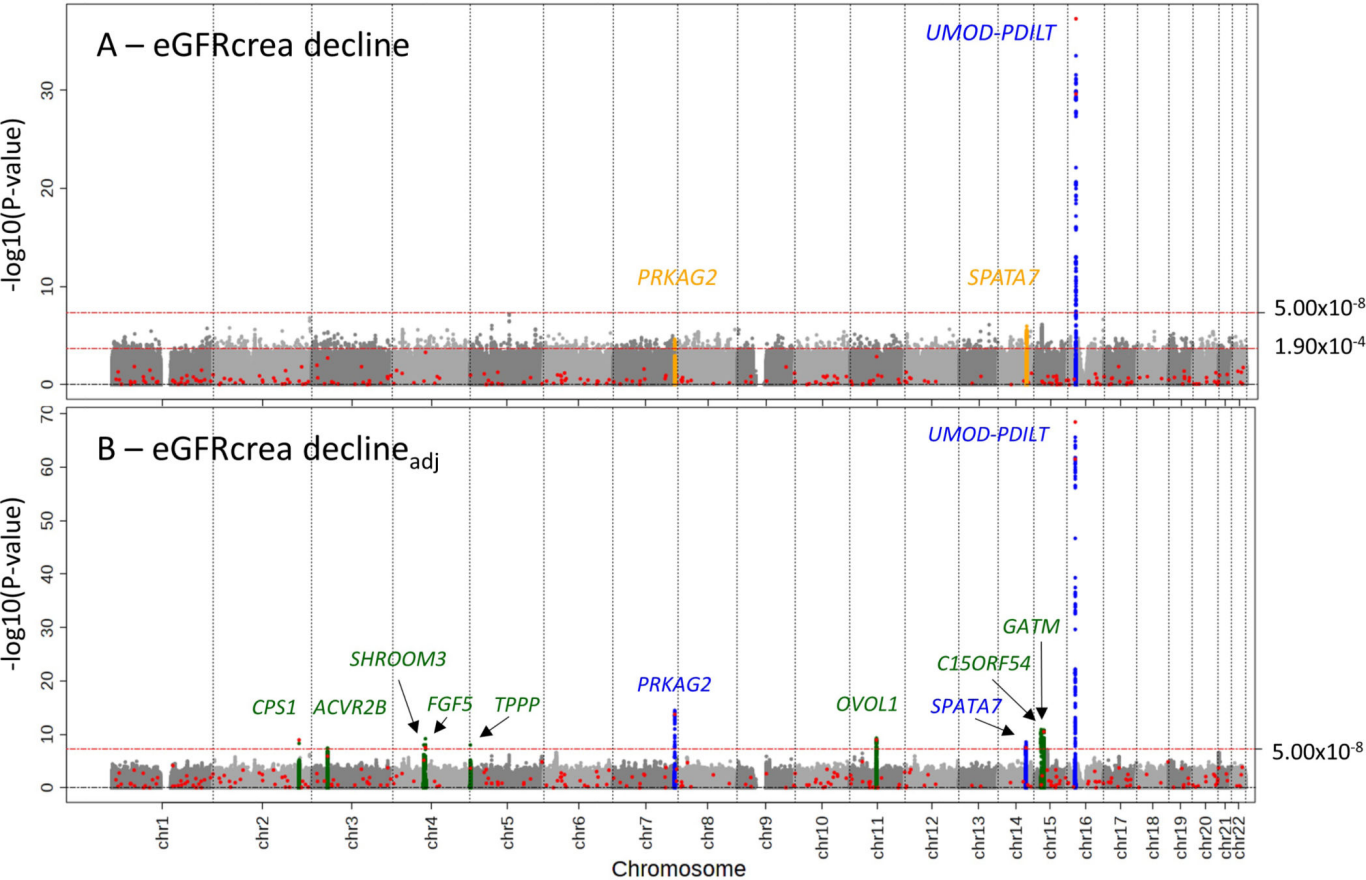
Eleven loci identified by GWAS for eGFR-decline unadjusted and/or adjusted for eGFR-baseline. We conducted GWAS for eGFR-decline baseline-unadjusted and baseline-adjusted (n up to 343,339 or 320,737, respectively). Shown are association P-values versus genomic position, identified loci annotated by nearest gene: (A) association for eGFR-decline baseline-unadjusted identified one genome-wide significant locus for decline (P<5×10^8^) and two Bonferroni-corrected significant loci among the 263 lead variants for cross-sectional eGFR^15^ outside of *UMOD-PDILT* (red dots, P<0.05/263=1.90×10^−4^; known locus for decline marked in blue; novel loci for this phenotype in orange); (B) association for eGFR-decline baseline-adjusted identified 8 additional loci (novel loci marked in green; known loci or loci already identified in (A) marked in blue). Altogether, 11 loci were identified with genome-wide significance for eGFR-decline unadjusted and/or adjusted for eGFR-baseline.

First, our genome-wide screen for eGFR-decline unadjusted for eGFR-baseline (n=343,339) identified two genome-wide significant independent variants near *UMOD-PDILT* ($P_{DECLINE}<5\times 10^{-8}$; Figure 1A, Table 1A): rs34882080, highly correlated with rs12917707 identified previously for this phenotype (r^2^=1.00)^20^, and rs77924615, known for altering *UMOD* expression and urine uromodulin^15^ and genome-wide significant for eGFR-decline for the first time.

Second, we evaluated the 263 additional lead variants known for cross-sectional eGFR GWAS^15^ for association with baseline-unadjusted eGFR-decline (candidate approach); we had a prior hypothesis that cross-sectionally known variants might also show association with eGFR-decline. We identified two additional variants for eGFR-decline near *PRKAG2* and *SPATA7*, both new loci for this phenotype, at Bonferroni-corrected significance ($P_{DECLINE}<0.05/263=1.90\times 10^{-4}$; Table 1A).

Third, our genome-wide screen for eGFR-decline adjusted for eGFR-baseline (n=320,737) identified 12 independent variants across 11 loci ($P_{DECLINE\text{\_}adj_{-}BL}<5\times 10^{-8}$, Figure 1B), including the four variants already identified by the baseline-unadjusted analyses (directly or via high correlation, r^2^≥0.9). The 8 variants additionally identified pointed to novel loci for this phenotype. Of these, 5 variants also showed directionally consistent, significant association for eGFR-decline unadjusted for eGFR-baseline (Bonferroni-corrected, $P_{DECLINE}<0.05/12=4.17\times 10^{-3}$; near *FGF5*, *OVOL1*, *TPPP*, *C15ORF54*, and *ACVR2B*; Table 1B), but 3 variants did not ($P_{DECLINE}$ from 0.156 to 0.710; near *GATM*, *CPS1*, *SHROOM3*, Table 1C).

Overall, we found 12 variants across 11 loci with genome-wide significant association for eGFR-decline unadjusted and/or adjusted for eGFR-baseline ($P_{DECLINE}$ or $P_{DECLINE\text{\_}adj\text{\_}BL}<5\times 10^{-8}$). All but one variant/locus were novel for this phenotype. All resided in loci known for eGFR cross-sectional GWAS^15^, but none was associated with DM-status (Supplementary Table S4).

The 12 variants’ associations showed no between-ancestry heterogeneity, stable statistics in various sensitivity analyses, and no impact by DM-adjustment (Supplementary Table S5&S6). Meta-analysis restricted to African American (n=9,038) did not identify associations for published *APOL1* risk variants^38^, but two other suggestive variants (Supplementary Table S7).

The 12 variants included 9 variants with non-zero effects on eGFR-decline unadjusted for eGFR-baseline (i.e. Bonferroni-corrected significant, i.e. $P_{DECLINE}<4.17\times 10^{-3}$).

### SNP-effects for eGFR-decline were larger when baseline-adjusted than baseline-unadjusted

**Figure 2:**
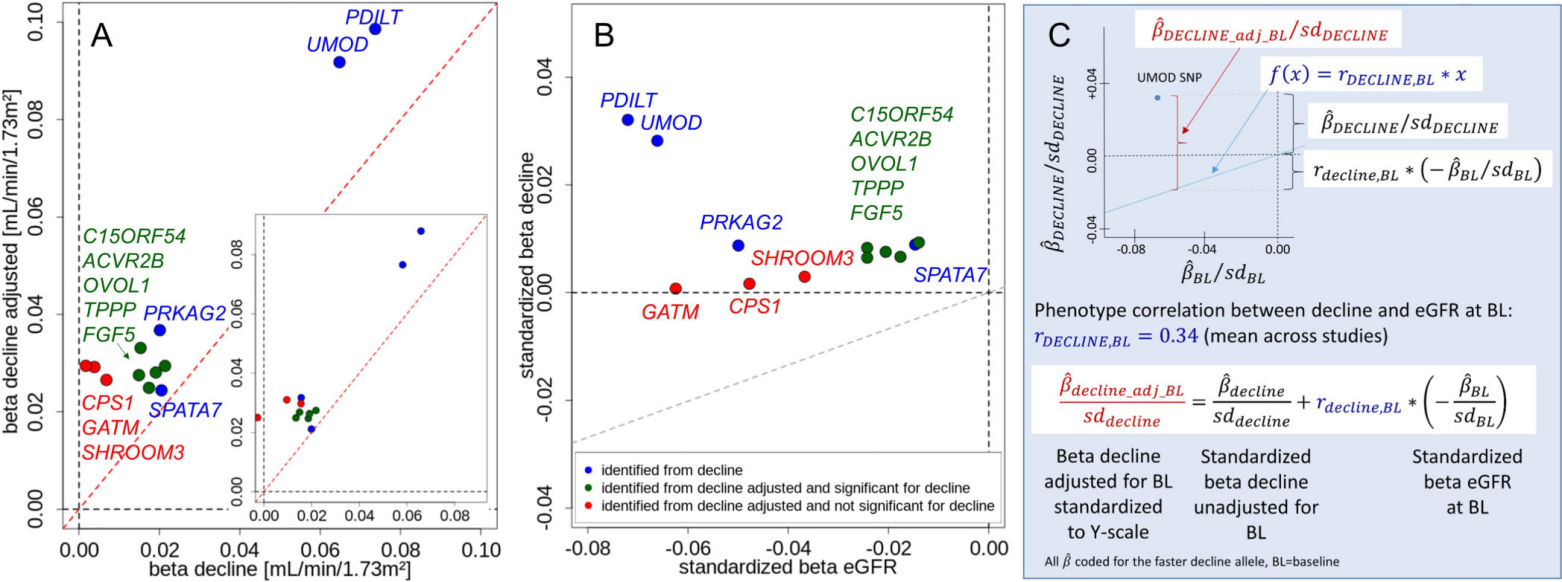
Relationship of SNP-effects on eGFR-decline baseline-unadjusted with baseline-adjusted effects for the 12 identified variants. Shown are: **(A)** SNP-effects per year and allele for eGFR-decline baseline-unadjusted (“decline”) versus eGFR-decline baseline-adjusted in all studies (n_decline_=343,339; n_decline-adj_=320,737) and restricted to studies where baseline-adjusted results were computed rather than formula-derived (inserted panel, n=103,970); red line indicates identify line); **(B)** standardized SNP-effects per year and allele for eGFR-decline baseline-unadjusted (${\hat{\beta }}_{DECLINE}/sd_{DECLINE}$, n=343,339) and per allele for cross-sectional eGFR on ln-scale (${\hat{\beta }}_{BL}/sd_{BL}$, n=765,348 ^15^); grey line indicates phenotype correlation line y=0.34*x (0.34=mean phenotype correlation across studies). For A&B: coding allele is the faster-decline allele (=cross-sectional eGFR-lowering allele). Color codes whether SNP was identified for decline baseline-unadjusted and/or baseline-adjusted. **(C)** Illustration of the SNP-effect for eGFR-decline baseline-adjusted (standardized to Y-scale) as a sum of the SNP-effect baseline-unadjusted (standardized) and the correlation-weighted SNP-effect on eGFR at baseline (standardized).

Several interesting aspects emerged when comparing genetic effect sizes of the 12 identified variants across models. First, we observed consistently larger effects for eGFR-decline baseline-adjusted than baseline-unadjusted (Figure 2A), also when restricting to studies where the baseline-adjusted model was directly computed (inserted small panel, Figure 2A). This, together with the smaller standard errors (Supplementary Figure S4B), explained the larger yield of genome-wide significant loci in the baseline-adjusted GWAS.

Second, we contrasted effect sizes for eGFR-decline unadjusted for eGFR-baseline with those for cross-sectional eGFR^15^ (Figure 2B). Three variants showed relatively extreme cross-sectional effects and no effect on decline (near *GATM*, *SHROOM3*, *CPS1*). For the other 9 variants, the faster-decline allele was always the cross-sectional eGFR-lowering allele (Spearman correlation coefficient=−0.32). A similar more schematic presentation (Figure 2C) illustrates the mathematical relationship between baseline-adjusted and baseline-unadjusted effect sizes (Supplementary Note S4). This yields a corollary on the directionality of baseline-adjusted effect sizes: when the faster-decline allele (i.e. ${\hat{\beta }}_{DECLINE}>0$) coincides with the baseline eGFR-lowering allele (i.e. ${\hat{\beta }}_{BL}<0$), then the baseline-adjusted eGFR-decline effect size is larger than baseline-unadjusted (i.e. ${\hat{\beta }}_{DECLINE\text{\_}adj\text{\_}BL}>{\hat{\beta }}_{DECLINE}$) – in theory. Our data confirmed this empirically (Figure 2A). The larger genetic effect sizes for eGFR-decline adjusted for eGFR-baseline are thus a direct consequence of the phenotypic and genetic correlation between eGFR-decline and eGFR-baseline. The genetic effect for eGFR-decline unadjusted for eGFR-baseline provides the relevant effect size for further use and to distinguish between a “genuine association with eGFR-decline” (9 variants) and a pure “collider bias” effect (3 variants).

### Four genes with compelling biological in-silico evidence mapped to novel eGFR-decline loci

All 11 identified loci for eGFR-decline coincided with loci detected for cross-sectional eGFR: among the 12 identified variants, 11 variants were genome-wide significant for cross-sectional eGFR^15^ and the variant near *TPPP* showed P=7.63×10^−6^ cross-sectionally with genome-wide significant variants nearby (Supplementary Figure S5A-C, Supplementary Note S5).

The 8 loci with genuine association for eGFR-decline included the well-known *UMOD-PDILT* locus. Biological evidence at the other seven loci was summarized using the Gene PrioritiSation tool^18^ generated from GWAS data on cross-sectional eGFR including evidence for SNP-modulated gene expression (eQTL, false-discovery-rate < 0.05): four lead variants or highly correlated proxies were eQTLs in tubule-interstitial kidney tissue with upregulating effects for *SPATA7* and *GALNTL5* (in *PRKAG2* locus, kidney-tissue specific), a downregulating effect for *FGF5* (kidney-tissue specific), and an upregulating effect for *TPPP* using NEPTUNE^39^. This supported these four genes in novel loci for eGFR-decline as kidney-tissue relevant and potentially causal genes for the association signals.

### SNPs for eGFR-decline showed SNP-by-age interaction on cross-sectional eGFR

**Figure 3:**
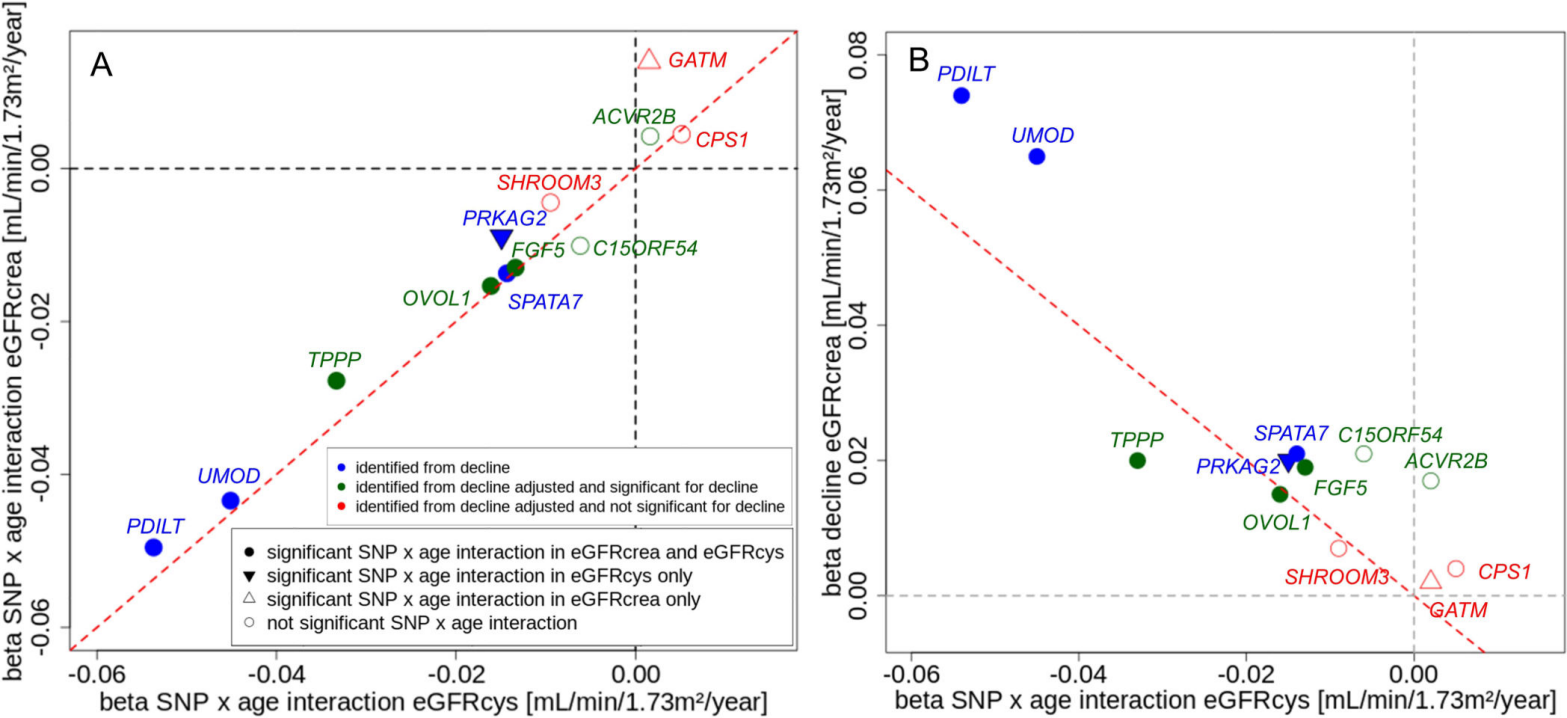
Relationship of SNP-by-age interaction effects for eGFRcys with those of eGFRcrea and with SNP-effects for eGFR-decline for the 12 identified variants. Shown are SNP-by-age interaction effect sizes per year and allele for cross-sectional eGFRcys (UK Biobank individuals independent from GWAS, n_SNPxage_=351,601; main age effect modelled non-linearly, main SNP-effect linearly, age effect and SNP effect in interaction term linearly, age centered at 50 years) versus: **(A)** SNP-by-age interaction effects on cross-sectional eGFRcrea (n_SNPxage_=351,462), **(B)** SNP-effects on eGFR-decline baseline-unadjusted per year and allele (n_decline_=343,339). Coding allele is the faster-decline allele (=cross-sectional eGFR-lowering allele); color code as in Figure 2; red line indicates identity line; symbol types code significance of interaction term (P< 0.05/12). Among the 9 SNPs with genuine eGFR-decline association, 7 SNPs showed interaction for eGFRcrea or eGFRcys (all negative), and all 3 SNPs without genuine eGFR-decline association showed no interaction for eGFRcys (one with positive significant interaction for eGFRcrea).

In the absence of birth cohort effects, we hypothesized that a SNP associated with eGFR-decline might also show an age-dependent association on cross-sectional eGFR, which is SNP-by-age interaction on cross-sectional eGFR. Of note, the age-effect on eGFR should reflect the age-effect on filtration rate, not on creatinine metabolism, within limits of uncertainty of the CKD-EPI formula^6^. To empirically assess this hypothesis, we tested the identified 12 SNPs for SNP-by-age interaction on cross-sectional eGFRcrea or eGFRcys in UK Biobank data, which was independent from and similarly-sized as the decline GWAS (n=351,462 or 351,601 for eGFRcrea or eGFRcys, respectively; Methods). For 8 of the 12 SNPs, we found SNP-by-age interaction for eGFRcrea and/or eGFRcys at Bonferroni-corrected significance ($P_{SNPxage}<0.05/12=4.17\times 10^{-3}$, Table 2). Interaction effect sizes were similar between eGFRcrea and eGFRcys (Figure 3A), except for the SNP near *GATM*.

The age-dependency of all SNP-effects and main age-effects were approximately linear (Supplementary Figure S6, Supplementary Note S6). The SNP-by-age interaction effect size can also be interpreted as the genetically modified age-effect on eGFR. This effect was large: e.g., 5 unfavorable alleles decreased eGFRcys by −0.136 mL/min/1.73m^2^ per year, which was ~10% of the overall age-effect on eGFRcys (−1.024 mL/min/1.73m^2^per year, Supplementary Note S6). SNP-by-age interaction effects on eGFRcys were highly correlated with SNP-effects on eGFR-decline (both in units of mL/min/1.73m^2^ per allele and year: “per year of age-difference between individuals” and “per year of person’s aging”, respectively; Figure 3B).

There was a noteworthy pattern with regard to presence and direction of SNP-by-age interaction: (i) among the 9 variants with genuine association for eGFR-decline, 7 variants showed significant SNP-by-age interaction on cross-sectional eGFRcys (Table 2A&B). All interaction effects were negative, i.e. the cross-sectional SNP-effect became larger (in absolute value) with older age. (ii) Among the three SNPs without genuine association for eGFR-decline, two showed no SNP-by-age interaction; the third (near *GATM*) showed SNPby-age interaction, but only for eGFRcrea and with positive direction (${\hat{\beta }}_{SNPxage}=+0.138$, $P_{SNPxage}=9.71\times 10^{-5}$). Thus, the *GATM* SNP-effect on cross-sectional eGFRcrea gets smaller (in absolute value) by higher age. This might be explained by *GATM* being the rate-limiting enzyme in creatine synthesis in muscle, age-related loss of muscle mass, and thus decreased creatinine production with increasing age - in line with the lack of interaction with eGFRcys, which is unrelated to muscle mass.

### A concept of three classes of SNPs for cross-sectional eGFR distinguished by their eGFR-decline association

**Figure 4:**
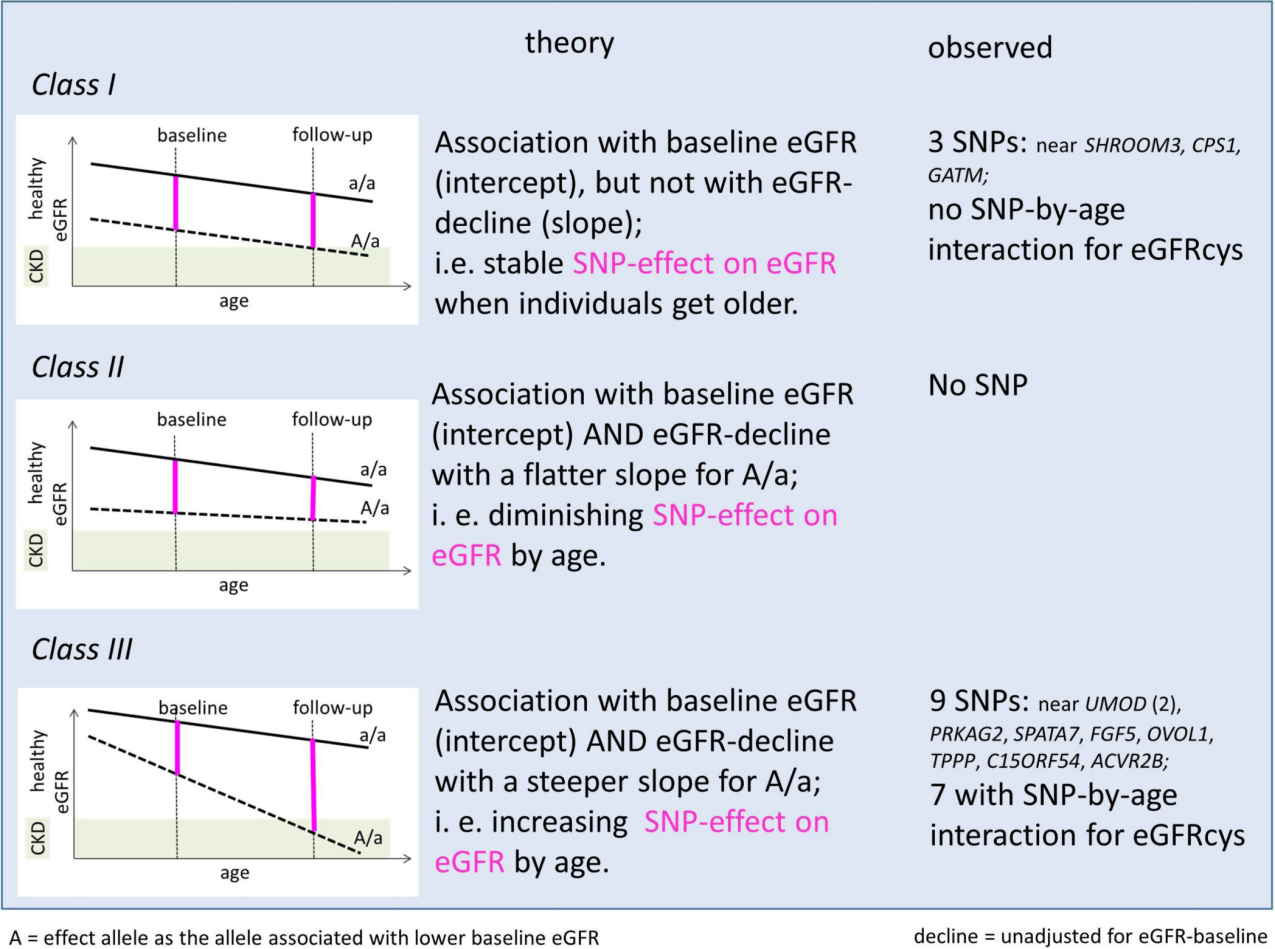
A concept for three classes of SNP-associations on cross-sectional eGFR distinguished by the presence and direction of the SNP-association with eGFR-decline. Let *A/a* be the genotype group of individuals with, on average, lower cross-sectional eGFR compared to *a/a* (*A*=effect allele). Let’s further assume that eGFR-declines monotonously by age (approximated as linear decline) and that there is no “cross-over” between genotype groups. Shown are (left) a graphical scheme, (middle) the theoretical association, (right) the observed SNPs in line with the respective class. In the three graphical schemes, **black** lines illustrate mean eGFR-decline by genotype group; SNP-effects on eGFR for these individuals captured cross-sectionally at different ages are magenta. When a cross-sectional study captures individuals of relevant ages, the SNP-effects on eGFR should show an interaction by age for *class II* and *class III* SNPs (positive and negative, respectively). The 9 variants with genuine eGFR-decline association were *class III*, while the other 3 variants were *class I*.

Our results suggested that SNPs for eGFR-decline were found among SNPs associated with eGFR cross-sectionally. This motivated the idea of, in theory, three classes of SNP-associations on cross-sectional eGFR (intercept) distinguished their eGFR-decline association unadjusted for eGFR-baseline (slope; Figure 4): no association with slope (*class I*), association of the eGFR-baseline lowering allele with flatter slope (*class II*), or association of the eGFR-baseline lowering allele with steeper slope (*class III*).

In our data, we found (i) three of the 12 SNPs as *class I*, in line with the lack of SNP-by-age interaction on eGFR cross-sectionally (judged for eGFRcys). (ii) No variant was *class II*, consistent with the lack of positive SNP-by-age interaction on eGFRcys. (iii) The 9 variants with genuine eGFR-decline association were *class III*, and 7 of these showed negative SNP-by-age interaction on eGFR. Thus, our data supported two classes of genetic effects on eGFR: no association with slope or steeper slope for the eGFR-lowering allele.

### Larger SNP-effects for eGFR-decline were observed in high-risk subgroups

Individuals with DM and/or CKD (defined as eGFR<60 mL/min/1.73m^2^) are at higher risk for CKD-progression and kidney failure, prompting us to quantify SNP-effects on eGFR-decline in these high-risk subgroups (meta-analysis for eGFR-decline unadjusted for eGFR-baseline restricted to DM or CKD at baseline, n= 37,375 or 26,653 respectively, Methods). For the 9 variants with genuine eGFR-decline association, we found almost all effects to be two- to four-fold larger in DM or in CKD compared to the overall analysis (Table 3, average effect size [mL/min/1.73m^2^/year and allele]: 0.061 in DM, 0.079 in CKD, compared to 0.030 overall).

To get an idea of the magnitude, we scaled the effects to “per 5 unfavorable average alleles” resulting in a decline of 0.305 in DM, 0.395 in CKD, compared to 0.150 mL/min/1.73m^2^/year overall. This compared well to the 9-variant weighted GRS effect on eGFR-decline per 5 unfavorable average alleles in the HUNT study (n=2,235 with DM, n=502 with CKD, n=46,328 overall; Methods): 0.219 in DM, 0.262 in CKD, and 0.102 mL/min/1.73m^2^/year overall (one-sided P=1.57×10^−5^, P=0.0193, and P=1.06×10^−34^, respectively).

The genetic effect sizes were also larger in the two subgroups when viewed relative to the phenotype variance (on the example of HUNT, Methods): rs77924615 variant (*UMOD-PDILT* locus) explained 0.38% of the eGFR-decline variance in DM, 0.47% in CKD, and 0.22% overall; the 9-variants jointly explained 1.14%, 1.48%, and 0.51%, respectively. Of note, the explained variance of eGFR-decline overall was comparable to the explained variance of cross-sectional eGFR (rs77924615: 0.21%; 9 variants: 0.62%), but narrow-sense heritability was smaller (Supplementary Note S7).

### *GALNTL5*, *SPATA7*, and *TPPP* were identified as candidates for CKD-progression

Variants associated with CKD-progression and mapped genes might help identify drug targets against disease progression^19^. We queried the 9 SNPs with genuine association for eGFR-decline for significant association with CKD-progression, i.e. whether they still showed significant association with eGFR-decline when focusing on individuals with CKD at baseline (judged at P<0.05/9=5.56×10^−3^, n up to 26,547). We found five such SNPs: (i) two in the *UMOD-PDILT* locus, which confirmed *UMOD* for a role in CKD-progression, (ii) three SNPs in novel loci for eGFR-decline, which mapped to three genes with eQTL in kidney tissue (*GALNTL5* in *PRKAG2* locus, kidney-tissue specific; *SPATA7*, and *TPPP*), making these compelling candidates as CKD-progression genes.

### Unfavorable GRS increased the risk for ESKD and AKI

Finally, we wanted to understand the cumulative impact of the 9 genuine eGFR-decline variants for severe clinical endpoints. We thus evaluated the 9-variant weighted GRS in cases-control studies for ESKD and AKI via logistic regression (n_cases_=2,068 and 3,878, n_controls_=4,640 and 11,634, respectively; Methods). The GRS effect per 5 unfavorable average alleles showed a significant OR=1.12 for ESKD (95%CI=0.99–1.23; one-sided P=0.033) and OR=1.18 for AKI (95% CI=1.09–1.27; one-sided P<0.0001 Table 4). When comparing the individuals with GRS ≥90^th^ versus ≤10^th^ percentile (i.e. ≥14.6 unfavorable alleles versus ≤8.3 in UK Biobank), we found a significant OR=1.35 for ESKD (95%CI=1.03–1.77, one-sided P=0.0157) and OR=1.27 (95%CI=1.08–1.50, one-sided P=0.002, Table 4).

## DISCUSSION

**Figure 5:**
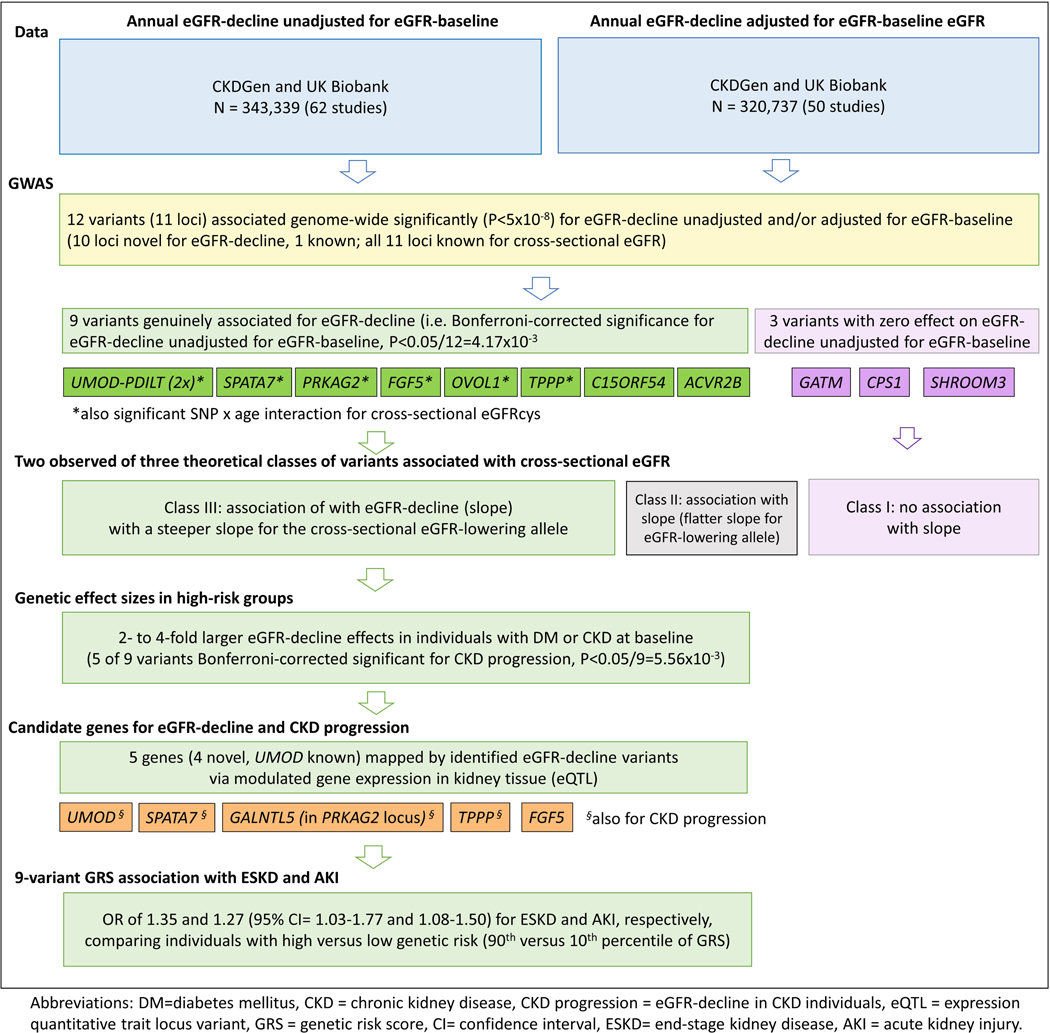
Data, analyses, and results in a nutshell.

Here, we provide data and results on a large longitudinal GWAS on annual eGFR-decline with >340.000 individuals from mostly population-based studies – to our knowledge the largest GWAS on annual eGFR-decline so far and probably one of the largest longitudinal GWAS of any trait. We identified 12 variants across 11 loci as genome-wide significant for annual eGFR-decline unadjusted and/or adjusted for eGFR-baseline (Figure 5). These included 9 variants across 8 loci with non-zero association unadjusted for eGFR-baseline, which we termed “genuinely” associated with eGFR-decline. Seven of these 9 variants also showed SNP-by-age interaction on cross-sectional eGFR in independent data of >350,000 individuals, while the three variants without genuine association did not. We generated and provide genome-wide summary statistics for eGFR-decline, CKD-progression, and eGFR-decline in DM. This data resource is informative for future meta-analyses, causal inference studies via Mendelian Randomization^40^, and drug development pipelines.

Clinically very important is our finding of the two-to four-fold larger genetic effects of almost all identified variants when focusing on individuals with DM or CKD at baseline, since these individuals are already at higher risk of kidney failure. This observation is in line with a “horse-racing effect”^41^ (“a faster horse is more likely observed up front”): individuals with an accumulation of faster eGFR-decline alleles are more likely observed with low eGFR at a given point in time, implying that these genetic effects might partly explain lower eGFR at baseline. A part of the larger eGFR-decline effect among CKD individuals might reflect collider bias. However, DM-status does not fulfill the characteristics of a collider for the SNP-associations with eGFR-decline (no impact by adjusting for DM-status, no SNP-association with DM-status), rendering the higher eGFR-decline effects in DM genuine.

The clinical relevance is further underscored by the 9-variant GRS being associated with increased risk of AKI and ESKD. This observation requires further analyses in future larger data. If substantiated, this may indicate a genetic risk of incomplete kidney function recovery after AKI and a genetic predisposition for ESKD.

The 9 identified variants across 8 loci included the *UMOD-PDILT* locus associated with eGFR-decline and CKD-progression, which is largely confirmatory but serves as proof-of-concept. A variant near *MIR378C* previously identified for CKD-progression^42^ (n~3000) was not confirmed here. Our other 7 loci are novel for eGFR-decline (near/in *PRKAG2-GALNTL5*, *SPATA7*, *FGF5*, *OVOL1*, *TPPP*, *C15ORF54*, and *ACVR2B*). These included at least three loci associated with CKD-progression (defined as eGFR-decline in individuals with CKD at baseline), mapping to the genes *GALNTL5*, *SPATA7* and *TPPP* by SNP-modulated expression in tubolo-interstitium^15,18^. These associations and genes for CKD-progression are in strong demand as genetic information on a disease progression phenotype, in order to help identify treatment^19^. Our data particularly flags *TPPP* by its locus’ large effect on eGFR-decline and CKD-progression, making it second only after *UMOD*. This also documents the value of longitudinal GWAS in revealing relevance of genes like *TPPP*: the *TPPP* locus was one of hundreds of small effect loci cross-sectionally, but among the few loci longitudinally.

Our results highlight some overlap of quantitative eGFR-decline genetics with binary extreme decline genetics^22^, but also distinction. All loci identified here were directionally consistent, nominally significant with “rapid3” and/or “CKDi25” (one-sided P<0.05) and two were genome-wide significant for rapid3 or CKDi25 (*UMOD*-*PDILT*, *PRKAG2*-*GALNTL5*). Particularly the loci identified here for CKD-progression, which is among individuals with CKD at baseline, complement the previously reported associations with CKDi25, which is among individuals without CKD at baseline. Methodologically, regression applied to a quantitative rather than dichotomized outcome has larger power and statistical advantages.

While all variants identified for eGFR-decline captured loci known from cross-sectional eGFR^15^, these associations are important on various accounts. First, the mere fact that eGFR-decline genetics is a subgroup of cross-sectional eGFR genetics is informative for future searches. Second, the finding that the full genetic signals were the same enabled the use of fine-mapping results from cross-sectional GWAS in >1 million individuals^18^ to prioritize genes also for longitudinal eGFR-decline. Third, all faster-decline alleles were the cross-sectional eGFR-lowering alleles. Together, this supported two classes of genetic variants for cross-sectional eGFR, distinguished by lack or presence of a slope effect, with steeper slope for the cross-sectional eGFR-lowering allele. The data rendered the third theoretical option, i.e. presence of a slope effect with flatter slope for the cross-sectional eGFR-lowering allele, void.

Some limitations warrant mentioning. Although this GWAS is currently the largest GWAS on eGFR-decline so far, more loci for eGFR-decline and CKD-progression might be detectable upon further increased sample size. The yield of eGFR-decline loci in >340,000 individuals was comparably low considering older GWAS for cross-sectional eGFR having already detected >50 loci in 170,000 individuals^43^. We used the CKD-EPI formula containing an ancestry term (Levey et al., Ann Intern Med), accounted for by ancestry-specific GWAS; future work should utilize the new ancestry-term-free CKD-EPI formula 2021 (Inker et al., NEJM). Evaluating the potential existence of sex-specific genetic effects on eGFR-decline is of interest, but was not addressed in this project. The target population is primarily population-based, including kidney diseases proportional to respective prevalence, and primarily European ancestry. Larger all-ancestry meta-analyses on eGFR-decline will open up opportunities to also utilize differential linkage disequilibrium between ancestries to help narrow down causal variants and genes. The interpretability of the SNP-by-age interaction on cross-sectional eGFR is limited to the age spectrum in the data (40–70 years) and by the power given the sample size; still, the sample size used was large and the age range typical also for most eGFR-decline GWAS studies. Two aspects need mentioning regarding the phenotype definition: uncertainty in eGFR-decline may be larger for studies with shorter follow-up, which decreases power, but measurement error in the outcome does not induce bias in linear regression^44^. By defining annual eGFR-decline from two eGFR assessments over time, our SNP associations capture only the linear component of decline. Serial eGFR assessments are better to characterize eGFR-trajectories, but at the cost of limiting sample size, since such studies are few and typically small. Furthermore, generalized additive mixed models for nonlinear eGFR-trajectories are complex and require particularly large sample sizes. The linear modelling of eGFR-decline is a reasonable approximation of monotonous decline, maintaining large sample sizes and limiting model complexity to be applicable for GWAS. Overall, the choice of the adjustment, target population, and phenotype definition are important to consider when interpreting results. While some modelling aspects are addressed here, other covariate adjustment or relative decline as phenotype might reveal further or other genetic loci. Future work is warranted to quantify effects in different target populations and the genetically determined shape of the decline, which requires more – and larger – longitudinal studies, ideally with more than two eGFR assessments over time.

Methodologically unique is our contrasting of GWAS SNP-associations on eGFR-decline for different covariate adjustment, which fills an important gap and helps design future studies. This is highly relevant, since covariate adjustment can alter GWAS findings and interpretation^28–31,45^. Adjusting for baseline DM-status had no impact, but genetic effects for eGFR-decline were larger when restricting to DM-individuals; this suggests DM-status as modulator for the SNP-association with eGFR-decline rather than mediator (i.e. in the causal pathway from SNP to eGFR-decline) or collider (i.e. generating biased association). Adjustment for eGFR-baseline yielded larger eGFR-decline effects and more genome-wide significant variants. Glymour et al. highlight that adjustment for baseline levels in analyses of change may help detect effects, but can induce spurious associations when the rate of change observed after baseline reflects a rate of change experienced in the past^36^. This might reflect the situation here rendering the larger genetic effects adjusted for eGFR-baseline - and the larger genetic effects when restricting to individuals with CKD at baseline – reflective of collider bias. Glymour et al. recommend the documentation of change effects without baseline adjustment^36^. In line with this, we considered a variant’s association with eGFR-decline genuine, when the variant reached genome-wide significance baseline-unadjusted or baseline-adjusted and Bonferroni-corrected significance baseline-unadjusted. The baseline-unadjusted model provides the relevant genetic effect sizes for eGFR-decline.

Interestingly, two of the three associations without genuine eGFR-decline association may relate to biomarker generation rather than kidney function: *GATM* and *CPS1*, known for a role in creatine biosynthesis^41^ and urea cycle^42^, respectively, reside in loci without supporting association with cross-sectional cystatin-based eGFR^18^. Conversely, the *SHROOM3* locus was associated with cystatin-based eGFR^18,15^ and experimental studies support a role of *SHROOM3* in kidney pathology^46–48^; thus, *SHROOM3* appears to have an effect on cross-sectional kidney function, but not on kidney function decline within the limits of detectability by sample size.

A further unique aspect of our work is the empirical evidence for a link between SNP-effects on eGFR-decline with SNP-by-age interaction effects on cross-sectional eGFR. By this, we provide important insights into the age-dependency of kidney function genetics as well as into the genetic dependency of aging eGFR in adult general populations, where “aging” includes onset of age-related diseases as they develop in populations. Considering the much broader availability of cross-sectional than longitudinal data, the further parallel exploitation of SNP-by-age interaction might be a promising route to help improve our understanding of the mechanisms of kidney function decline over time.

In summary, we provide GWAS summary statistics, identified genetic loci, and prioritized genes for kidney function decline and CKD-progression. While *UMOD* has drawn attention already, *GALNTL5*, *SPATA7*, and *TPPP* may now receive more focus as therapeutic targets for disease progression. Our exploration of different covariate adjustment and the comparison to age-dependency of SNP-effect on eGFR cross-sectional provides important insights into the interpretation of these effects. With the emerging large biobank data linking medical records, longitudinal GWAS will become very important in the future. Our methodological framework is informative and applicable also generally for longitudinal phenotypes.

### Availability of data and materials

To support future work, we provide genome-wide summary statistics on eGFR-decline unadjusted for eGFR-baseline (adjusted for age, sex and DM-status) overall and restricted to individuals with DM or CKD at baseline (all adjusted for age and sex) (https://www.uniregensburg.de/decline and http://ckdgen.imbi.uni-freiburg.de). The summary statistics on eGFR-decline in individuals with CKD at baseline can be considered genetic effects on CKD-progression. We also provide genome-wide summary statistics on eGFR-decline adjusted for eGFR-baseline (additionally to adjustment for age and sex), but these summary statistics should be used with great care and an understanding that beta-estimates are subject to collider bias. For quantification of the genetic effect on eGFR-decline, the results unadjusted for eGFR-baseline should be utilized.

## Supplementary Material

1 Supplementary Methods
**Note S1.** Equivalence of DM-adjusted versus not DM-adjusted GWAS on eGFR-decline in the validation meta-analysis
**Note S2.** Formula-based covariate adjustment using GWAS summary statistics
**Note S3.** Validation of the formula-derived association for eGFR-decline adjusted for eGFR-baseline
**Note S4.** Graphical illustration of the relationship between SNP-effects on eGFR-decline unadjusted and adjusted for eGFR-baseline
**Note S5.** Comparison of the signals for eGFR-decline unadjusted and adjusted for eGFR-baseline and cross-sectional eGFR for the 11 identified loci
**Note S6.** Age-dependency of SNP-effects and main age effect on eGFR
**Note S7.** Narrow-sense heritability
**Figure S1.** Meta-analysis workflow
**Figure S2.** Study-specific median annual eGFR-decline versus sample size, follow-up time and median age
**Figure S3.** Influence of alternative adjustments for age on eGFR-decline in UK Biobank
**Figure S4A.** No influence from adjusting SNP-associations for eGFR-decline for diabetes mellitus (DM)
**Figure S4B.** Differences between SNP-association for eGFR-decline unadjusted versus adjusted for eGFR-baseline
**Figure S4C.** Validation of formula-derived adjustment for eGFR-baseline in eGFR-decline associations (part 1).
**Figure S4D.** Validation of formula-derived adjustment for eGFR-baseline in eGFR-decline associations (part 2)
**Figure S5.** Region plots of loci identified for eGFR-decline unadjusted and adjusted for eGFR-baseline
**Figure S6.** Age-dependency of eGFR and age-dependency of the variant effects on eGFR in UK Biobank
**Table S1.** Description of participating studies: study design
**Table S2.** Description of participating studies: genotyping and imputation
**Table S3.** Description of participating studies: phenotype distribution
**Table S4.** The 12 identified variants for eGFR-decline were associated with other kidney phenotypes, but not with DM-status
**Table S5.** The 12 identified variants for eGFR-decline do not show heterogeneity between ancestries and FHS is not an influential study
**Table S6.** No influence by DM-adjustment versus no DM-adjustment or by model-based versus formula-based adjusting for baseline eGFR (BL) on the 12 variants’ association with eGFR-decline
**Table S7.** Association of *APOL1* risk variants in African American and European CKDGen studies
Extended acknowledgements, study funding information and author contributions
Supplementary References
Author contributions

## Figures and Tables

**Table 1:** Twelve independent variants in 11 loci identified for association with eGFR-decline unadjusted and adjusted for eGFR-baseline. We conducted GWAS for eGFR-decline baseline-unadjusted and baseline-adjusted (“decline”, n up to 343,339; decline_adj_, n up to 320,737). This identified **(A)** 2 variants with genome-wide significance for eGFR-decline baseline-unadjusted (*UMOD*-*PDILT*, P_decline_<5×10^−8^) and 2 further variants in a candidate search of the 263 variants known for cross-sectional eGFR^15^ outside *UMOD*-*PDILT*, judged at Bonferroni-corrected significance (P_decline_<0.05/263=1.90×10^−4^; *PRKAG2*, *SPATA7*), **(B)** 5 variants with genome-wide significance for eGFR-decline baseline-adjusted AND Bonferroni-corrected significant baseline-unadjusted (P_decline-adj-BL_<5×10^−8^, P_decline_ <0.05/12=4.17×10^−3^), **(C)** 3 variants with genome-wide significance for eGFR-decline baseline-adjusted but not significantly associated baseline-unadjusted (P_decline-adj-BL_<5×10^−8^, P_decline_≥4.17×10^−3^). For each identified variant, we show results for decline (baseline-unadjusted), for decline baseline-adjusted, and for cross-sectional eGFR^15^. Beta-estimates are in mL/min/1.73^2^ per year and per faster-decline allele; significant P-values are stated in bold.

						decline		decline_adj_		cross-sectional	
SNPID	Locus Name	Chr	Pos	EA/OA	EAF	Beta	P	Beta	P	Beta	P
**A from GWAS/candidate search for decline (baseline-unadjusted)**											

rs34882080	*UMOD-PDILT*	16	20,361,441	a/g	0.815	0.065	**2.45×10** ^**−30**^	0.092	**3.31×10** ^**−62**^	−0.009	**2.86×10** ^**−95**^
rs77924615	*UMOD-PDILT*	16	20,392,332	g/a	0.798	0.074	**5.30×10** ^**−38**^	0.099	**3.75×10** ^**−69**^	−0.010	**1.45×10** ^**−138**^
rs10254101	*PRKAG2* *	7	151,415,536	t/c	0.276	0.020	**4.10×10** ^**−05**^	0.037	**1.78×10** ^**−14**^	−0.007	**1.85×10** ^**−67**^
rs1028455	*SPATA7* *	14	88,829,975	t/a	0.657	0.021	**5.90×10** ^**−06**^	0.024	**3.43×10** ^**−08**^	−0.002	**4.78×10** ^**−10**^

**B from GWAS for decline_adj_, with association for decline (baseline-unadjusted)**											

rs1458038	*FGF5*	4	81,164,723	c/t	0.690	0.019	**3.87×10** ^**−05**^	0.028	**6.85×10** ^**−10**^	−0.003	**7.49×10** ^**−24**^
rs4930319	*OVOL1*	11	65,555,458	c/g	0.333	0.015	**9.93×10** ^**−04**^	0.028	**5.27×10** ^**−10**^	−0.003	**2.21×10** ^**−24**^
rs434215	*TPPP* ^§^	5	699,046	a/g	0.277	0.020	**3.70×10** ^**−04**^	0.032	**7.19×10** ^**−09**^	−0.003	7.63×10^−06^
rs28857283	*C15ORF54* ^†^	15	39,224,711	g/a	0.656	0.021	**1.47×10** ^**−06**^	0.030	**1.31×10** ^**−11**^	−0.002	**6.20×10** ^**−09**^
rs13095391	*ACVR2B*	3	38,447,232	a/c	0.502	0.017	**1.77×10** ^**−04**^	0.025	**4.03×10** ^**−08**^	−0.003	**6.57×10** ^**−15**^

**C from GWAS for declineadj, without association for decline (baseline-unadjusted)**											

rs9998485	*SHROOM3*	4	77,362,445	a/g	0.466	0.007	0.156	0.027	**9.84×10** ^**−09**^	−0.005	**1.22×10** ^**−41**^
rs1047891	*CPS1*	2	211,540,507	a/c	0.293	0.004	0.441	0.029	**1.15×10** ^**−09**^	−0.007	**1.18×10** ^**−75**^
rs2453533	*GATM*	15	45,641,225	a/c	0.422	0.002	0.710	0.029	**1.72×10** ^**−11**^	−0.009	**4.57×10** ^**−141**^

**SNPID**=Variant identifier on GRCh37, **Locus name**=Nearest Gene, **Chr** and **Position**=Chromosome and Position on GRCh37, **EA/OA**=Effect allele / other allele, **EAF**=effect allele frequency, **beta** and **P**=genetic effect coefficient of association and association P-value.

* In *PRKAG2* and *SPATA7* loci, variants with smallest P_decline_ (rs73158188 and rs7160717, respectively) were highly correlated with these candidate-based variants (r^2^=1.00 and 0.93, respectively).

§ Since the *TPPP* locus lead variant had imputation quality <0.6 in 45% of the studies (median 0.64), we analyzed this locus omitting the imputation quality filter (with filter: decline_adj_ beta=0.033, P=1.00×10^−8^; decline beta=0.015, P=0.039; median imputation quality=0.74).

† In the *C15ORF54* locus, the identified lead variant for decline was highly correlated with a 2^nd^ signal lead variant for cross-sectional eGFR (rs28833881, r^2^=0.90), but not with the 1^st^ signal lead variant (rs12913015, r^2^=0.04).

**Table 2:** SNP-by-age interaction for cross-sectional eGFR for the 12 identified variants. For the 12 identified variants, we conducted SNP-by-age interaction analysis for cross-sectional eGFRcrea and eGFRcys in UK Biobank (excluding individuals from decline GWAS; n=351,462 for eGFRcrea, n=351,601 for eGFRcys; main age effect modelled non-linearly, main SNP effect linearly, age centered at 50 years). The interaction term (age effect and SNP effect modelled linearly) was judged at Bonferroni-corrected significance level (P<0.05/12=4.17×10^−3^). Beta-estimates are in mL/min/1.73^2^ per year and per cross-sectional eGFR-lowering allele (which was equivalent to faster-decline allele for each SNP); significant P-values are stated in bold.

SNPID	Locus Name	EA/OA	SNP x age interaction eGFRcrea		SNP x age interaction eGFRcys	
			Beta	P	Beta	P
**A from GWAS/candidate search for decline (baseline-unadjusted)**						

rs34882080	*UMOD-PDILT*	a/g	−0.043	**5.53×10** ^**−22**^	−0.045	**2.37×10** ^**−17**^
rs77924615	*UMOD-PDILT*	g/a	−0.050	**2.55×10** ^**−29**^	−0.054	**6.59×10** ^**−25**^
rs10254101	*PRKAG2*	t/c	−0.009	0.0263	−0.015	**9.84×10** ^**−04**^
rs1028455	*SPATA7*	t/a	−0.014	**2.19×10** ^**−04**^	−0.014	**1.06×10** ^**−03**^

**B from GWAS for decline_adj_, with association for decline (baseline-unadjusted)**						

rs1458038	*FGF5*	c/t	−0.013	**7.11×10** ^**−04**^	−0.013	**3.12×10** ^**−03**^
rs4930319	*OVOL1*	c/g	−0.015	**2.55×10^−05^**	−0.016	**1.84×10^−04^**
rs434215	*TPPP*	a/g	−0.028	**1.02×10** ^**−10**^	−0.033	**5.02×10^−11^**
rs28857283	*C15ORF54*	g/a	−0.010	5.09×10^−03^	−0.006	0.148
rs13095391	*ACVR2B*	a/c	0.004	0.227	0.002	0.695

**C from GWAS for decline_adj_, without association for decline (baseline-unadjusted)**						

rs9998485	*SHROOM3*	a/g	−0.004	0.206	−0.009	0.022
rs1047891	*CPS1*	a/c	0.004	0.228	0.005	0.244
rs2453533	*GATM*	a/c	0.014	**9.71×10** ^**−05**^	0.002	0.722

**SNPID**=Variant identifier on GRCh37, **Locus name**=Nearest Gene, **EA/OA**=Effect allele / other allele, **Beta** and **P**=genetic effect and association P-value. The *TPPP* variant rs434215 is well-imputed in the UK Biobank (imputation quality=0.82).

**Table 3:** The 9 variants’ effects on eGFR-decline unadjusted for eGFR-baseline in high-risk subgroups. Shown are the 9 variants with genuine association for eGFR-decline for their association with eGFR-decline restricted to individuals with baseline diabetes mellitus (DM, n up to 38,206) or baseline CKD (i.e. eGFR<60 mL/min/1.73m^2^, n up to 26,653). Beta-estimates and 95% confidence intervals (CI) are in mL/min/1.73m^2^ per year and per faster-decline allele.

SNPID	Locus Name	Decline among DM at baseline		Decline among CKD at baseline		Decline among all	
		Beta	95% CI	Beta	95% CI	Beta 95% CI
**A from GWAS/candidate search for decline (baseline-unadjusted)**							

rs34882080	*UMOD-PDILT*	0.159*	0.108, 0.211	0.138*	0.074, 0.203	0.065	0.054, 0.076
rs77924615	*UMOD-PDILT*	0.136*	0.084, 0.189	0.167*	0.099, 0.235	0.074	0.063, 0.085
rs10254101	*PRKAG2*	0.065	0.020, 0.110	0.095*	0.042, 0.148	0.020	0.010, 0.030
rs1028455	*SPATA7*	0.030	−0.011, 0.071	0.085*	0.034, 0.135	0.021	0.012, 0.029

**B from GWAS for decline_adj_, with association for decline (baseline-unadjusted)**							

rs1458038	*FGF5*	0.030	−0.013, 0.072	0.040	−0.013, 0.092	0.019	0.010, 0.028
rs4930319	*OVOL1*	0.021	−0.021, 0.062	0.031	−0.019, 0.080	0.015	0.006, 0.024
rs434215	*TPPP* ^§^	0.031	−0.024, 0.086	0.112*	0.043, 0.180	0.020	0.006, 0.035
rs28857283	*C15ORF54*	0.046	0.005, 0.086	0.042	−0.007, 0.091	0.021	0.013, 0.030
rs13095391	*ACVR2B*	0.029	−0.021, 0.080	0.006	−0.054, 0.066	0.017	0.008, 0.026

**Average**		**0.061**		**0.079**		**0.030**	

**SNPID**=Variant identifier on GRCh37, **Locus name**=Nearest Gene, **Beta**=genetic effect of genetic association where the effect alleles is the same as in Table 1 and Table 2, **95% CI** = 95% confidence interval of Beta (Beta±1.96*standard error of the association).

* Statistically significant different from zero (P< 0.05/9=5.56×10^−3^).

§ Since the lead variant had imputation quality <0.6 in 45% of the studies (median 0.64), we analyzed this variant omitting the imputation quality filter (with filter: decline among DM at baseline beta=−0.093, P=0.338, n=927; decline among eGFR <60 mL/min/1.73m^2^ beta=0.022, P=0.618, n=2924; median imputation quality=0.74).

**Table 4:** Genetic risk score (GRS) analyses for end-stage kidney disease (ESKD) and Acute Kidney Injury (AKI). In 3 case-control studies for ESKD and one for AKI, we computed the weighted GRS across the 9 eGFR-decline variants (counting the faster-decline alleles, weighted by effect size for eGFR-decline unadjusted for eGFR-baseline; divided by sum of weights and multiplied by 9, i.e. scaled as 0 to 18). Shown are odds ratios (OR), 95% confidence intervals (CI) and P-values (one-sided) for the quantitative GRS association (per 5 “average” unfavorable alleles) and for a high versus low GRS association (≥95^th^ versus ≤5^th^, ≥90^th^ versus ≤10^th^ GRS percentiles derived in UK Biobank) with (A) ESKD and (B) AKI. Associations are derived by logistic regression adjusted for matching variables age-group and sex (AKI additionally for principal components).

		Per 5 unfavorable average alleles			High versus low GRS group					
					5% versus 95%			10% versus 90%		
Study	Number of Cases	Number of Controls	OR	95% CI	P (1-sided)	OR	95% CI	P (1-sided)	OR	95% CI	P (1-sided)
**(A) ESKD** (cases: ICD10 code N18.0 or N18.5; controls: no ICD10 code N18, eGFR>60 mL/min/1.73m^2^, frequency-matched by age-group and sex)											
4D_KORA-F3	1,100	1,601	1.122	0.925,1.362	0.121	1.26	0.669,2.377	0.237	1.526	0.978,2.379	0.0313
GENDIAN_KORA-F4	470	1,545	1.146	0.923,1.423	0.108	0.954	0.468,1.946	0.449	1.036	0.625,1.719	0.445
UKBBCa_co	498	1,494	1.085	0.885,1.330	0.216	1.220	0.639,2.329	0.273	1.479	0.921,2.373	0.0525

**Meta-analysis**	**2,068**	**4,640**	1.117	0.993,1.256	**0.0329**	**1.150**	**0.785,1.686**	**0.236**	**1.349**	**1.027,1.773**	**0.0157**



**(B) AKI** (cases: ICD 10 code N17; controls: no ICD10 code N17, eGFR>60 mL/min/1.73m^2^, frequency-matched by age-group and sex)											

UKBBCaCo	3,878	11,634	1.179	1.095,1.270	6.47×10^−06^	1.524	1.204,1.931	4.70×10^−04^	1.272	1.080,1.499	1.97×10^−03^

**Study**=Study name, **OR**=Odds Ratio of the GRS-association, **95% CI**=95% confidence interval of the association, **P (1-sided)**=1-sided association P-value, **ESKD**=End-stage Kidney Disease, Individuals analyzed here are distinct from the eGFR-decline GWAS except for the KORA-F3 and KORA-F4 controls. **AKI**=Acute Kidney Injury, UKBBCaCo=cases and controls from UK Biobank distinct from UK Biobank study participants used in the GWAS for eGFR decline.
