# Supplementary material for: Genetic loci and prioritization of genes for kidney function decline derived from a meta-analysis of 62 longitudinal genome-wide association studies
Source: Kidney Int. Author manuscript; Available in PMC 2023 Mar 23. (PMC10034922; doi:10.1016/j.kint.2022.05.021)
Supplement: 1 — Supplementary Methods Note S1. Equivalence of DM-adjusted versus not DM-adjusted GWAS on eGFR-decline in the validation meta-analysis Note S2. Formula-based covariate adjustment using GWAS summary statistics Note S3. Validation of the formula-derived association for eGFR-decline adjusted for eGFR-baseline Note S4. Graphical illustration of the relationship between SNP-effects on eGFR-decline unadjusted and adjusted for eGFR-baseline Note S5. Comparison of the signals for eGFR-decline unadjusted and adjusted for eGFR-baseline and cross-sectional eGFR for the 11 identified loci Note S6. Age-dependency of SNP-effects and main age effect on eGFR Note S7. Narrow-sense heritability Figure S1. Meta-analysis workflow Figure S2. Study-specific median annual eGFR-decline versus sample size, follow-up time and median age Figure S3. Influence of alternative adjustments for age on eGFR-decline in UK Biobank Figure S4A. No influence from adjusting SNP-associations for eGFR-decline for diabetes mellitus (DM) Figure S4B. Differences between SNP-association for eGFR-decline unadjusted versus adjusted for eGFR-baseline Figure S4C. Validation of formula-derived adjustment for eGFR-baseline in eGFR-decline associations (part 1). Figure S4D. Validation of formula-derived adjustment for eGFR-baseline in eGFR-decline associations (part 2) Figure S5. Region plots of loci identified for eGFR-decline unadjusted and adjusted for eGFR-baseline Figure S6. Age-dependency of eGFR and age-dependency of the variant effects on eGFR in UK Biobank Table S1. Description of participating studies: study design Table S2. Description of participating studies: genotyping and imputation Table S3. Description of participating studies: phenotype distribution Table S4. The 12 identified variants for eGFR-decline were associated with other kidney phenotypes, but not with DM-status Table S5. The 12 identified variants for eGFR-decline do not show heterogeneity between ancestries and FHS is not an influential stud [file NIHMS1841277-supplement-1.pdf]

SUPPLEMENTARY ONLINE MATERIAL

Genetic loci and prioritized genes for kidney function decline from a meta-analysis of 62 longitudinal genome-wide association studies

Supplementary Methods

Supplementary Notes

Supplementary Note S1	Equivalence of DM-adjusted versus not DM-adjusted GWAS on eGFR-decline in the validation meta-analysis
Supplementary Note S2	Formula-based covariate adjustment using GWAS summary statistics
Supplementary Note S3	Validation of the formula-derived association for eGFR-decline adjusted for eGFR-baseline
Supplementary Note S4	Graphical illustration of the relationship between SNP-effects on eGFR-decline unadjusted and adjusted for eGFR-baseline
Supplementary Note S5	Comparison of the signals for eGFR-decline unadjusted and adjusted for eGFR-baseline and cross-sectional eGFR for the 11 identified loci
Supplementary Note S6	Age-dependency of SNP-effects and main age effect on eGFR
Supplementary Note S7	Narrow-sense heritability

Supplementary Figures

Supplementary Figure S1	Meta-analysis workflow
Supplementary Figure S2	Study-specific median annual eGFR-decline versus sample size, follow-up time and median age
Supplementary Figure S3	No influence of alternative adjustments for age on eGFR-decline in UK Biobank
Supplementary Figure S4A	No influence from adjusting SNP-associations for eGFR-decline for diabetes mellitus (DM)
Supplementary Figure S4B	Differences between SNP-association for eGFR-decline unadjusted versus adjusted for eGFR-baseline
Supplementary Figure S4C	Validation of formula-derived adjustment for eGFR-baseline in eGFR-decline associations (part 1).
Supplementary Figure S4D	Validation of formula-derived adjustment for eGFR-baseline in eGFR-decline associations (part 2)
Supplementary Figure S5	Region plots of loci identified for eGFR-decline unadjusted and adjusted for eGFR-baseline
Supplementary Figure S6	Age-dependency of eGFR and age-dependency of the variant effects on eGFR in UK Biobank

Supplementary Tables

Supplementary Table S1 Description of participating studies: study design

Supplementary Table S2 Description of participating studies: genotyping and imputation

Supplementary Table S3 Description of participating studies: phenotype distribution

Supplementary Table S4 The 12 identified variants for eGFR-decline were associated with other kidney phenotypes, but not with DM-status

Supplementary Table S5 The 12 identified variants for eGFR-decline do not show heterogeneity between ancestries and FHS is not an influential study

Supplementary Table S6 No influence by DM-adjustment versus no DM-adjustment or by model-based versus formula-based adjusting for baseline eGFR (BL) on the 12 variants' association with eGFR-decline

Supplementary Table S7 Association of *APOL1* risk variants in African American and European CKDGen studies

Extended acknowledgements, study funding information and author contributions

Supplementary Methods

General approach for GWAS meta-analysis

An analysis plan and standardized scripts for phenotype generation and GWAS analyses were developed and implemented in all 61 CKDGen studies and UK Biobank. The 61 CKDGen studies consisted of 58 studies that were long-term partners of CKDGen ("old" CKDGen studies) and three studies that have joined CKDGen more recently allowing for more elaborate analyses (AugUR, HUNT and MGI; extended analysis plan, see below). Most studies were population-based and thus including individuals with specific kidney diseases according to the prevalence in the general population. Each study conducted GWAS analyses according to this pre-defined plan, separately by ancestry (if applicable). Ancestry was defined by genetic principal components or participants' self-report. For each study, phenotypic information and genome-wide summary statistics per SNP were transferred to the meta-analysis centers.

Each study had been conducted according to the declaration of Helsinki. The studies have been approved by each local ethics committee. All participants in all studies provided written informed consent.

Meta-analyses were conducted, significant variants identified and respective locus regions selected. A GWAS across all available studies was shown to be advantageous over conducting a discovery followed by a replication stage on selected variants[S1,S2]. Therefore, rather than conducting a discovery GWAS in old CKDGen studies and a replication in recently joined CKDGen studies and UK Biobank, we included all studies into the GWAS meta-analysis on eGFR decline.

Phenotype definition

In each contributing study, serum creatinine was measured at least two times, utilizing two measurements at largest time distance (study-specific details in **Supplementary Table S1**). When measurements were obtained by Jaffé assay (before 2009), creatinine measurements were calibrated (multiplying by 0.95[S3]). Serum creatinine measured at baseline and follow-up was used to estimate eGFR at baseline and follow-up, respectively, according to the Chronic Kidney Disease Epidemiology Collaboration (CKD-EPI) equation[S4]. This equation contains an age, sex, and ancestry term for a best fit of creatinine-based eGFR to measured GFR. At baseline and follow-up, eGFR was winsorized at 15 and 200 mL/min/1.73m^2. Annual eGFR-decline was defined as "- (eGFR at follow-up - eGFR at baseline) / number of years of follow-up"; thus, eGFR-decline is positive when eGFR is lower at follow-up compared to baseline and comparable across studies with different follow-up length.

In each study, eGFR-decline was analyzed overall and restricted to individuals with CKD or DM at baseline. CKD at baseline was defined as eGFR<60 mL/min/1.73m^2 at baseline. In CKDGen, DM at baseline was defined as fasting plasma glucose ≥126 mg/dl (7.0 mmol/L)

or diabetes therapy, or (fasting glucose unavailable) as non-fasting plasma glucose ≥200 mg/dl (11.0 mmol/L) or diabetes therapy, or (glucose unavailable) as self-reported diabetes. For UK Biobank, DM was defined as HbA1c≥48 mmol/mol or diabetes therapy.

Study-specific generation of outcome variables according different adjustment models

In each study, different models for the SNP-association with annual eGFR-decline as outcome were computed genome-wide: (i) adjusted for age, sex, and DM and applied to all individuals (“decline DM-adjusted”); (ii) adjusted for age and sex restricted to individuals with DM or CKD at baseline (“decline in DM”, “decline in CKD”). In the recently joined CKDGen studies and UK Biobank, an extended suite of models was applied: additional analyses were (iii) adjusted for age and sex using all individuals (“decline”), (vi) adjusted for age, sex and eGFR baseline using all individuals (eGFR baseline on log-scale, ln(eGFR), “decline adjusted for baseline”). Further study-specific adjustments were applied (as applicable), including genetic principal components to account for population substructure.

These adjustments were implemented by generating residuals of annual eGFR-decline adjusted for the respective covariates and using these residuals as outcome in GWAS. This is a standard approach yielding comparable results to using the unadjusted phenotype as outcome in GWAS adjusting for the respective covariates. The utilized approach implies fewer covariates in GWAS being computationally more efficient. We standardized the creation of these outcome variables for GWAS by providing a centrally developed script, which also provided descriptive statistics on the study-specific phenotype.

Genotyping, imputation, and study-specific GWAS

In each study, genotyping was conducted using Affymetrix and Illumina arrays (**Supplementary Table S2**). Imputation was performed using 1000 Genomes[S5] phase 1 or phase 3, the Haplotype Reference Consortium[S6] v1.1 or customized reference panels, annotating all variants on the GRCh build 37 reference build; imputed genotypes were coded as allelic dosages and imputation quality was provided as IMPUTE2[S7] info score, MACH/minimac[S8] RSQ or similar**;** quality control before and after imputation was conducted study-specifically (**Supplementary Table S2**).

In each study, GWAS analyses were conducted according to the centrally defined analysis plan. CKDGen studies included different ancestries (European, African American, East Asian, South Asian, and Hispanic) and contributed analyses ancestry-specific. Since most CKDGen studies individuals were European ancestry (94.90%), UK Biobank analyses focused on unrelated European ancestry individuals where two assessments of eGFR were available (n=15,442). For each GWAS, linear regression on the respective outcome variable was computed per SNP (modelled as allele dosages linearly) adjusted for principle

components and other study-specific covariates as applicable (**Supplementary Table S2**). This yielded three GWAS results for “old” and recently joined CKDGen studies (decline DM-adjusted, decline in DM, decline in CKD) and two further GWAS results for recently joined CKDGen studies and UK Biobank (decline, decline adjusted for baseline). Summary statistics were collected and quality controlled centrally with GWAtoolbox[S9].

Study-specific summary statistics for decline adjusted for baseline

As noted above, GWAS results on eGFR-decline adjusted for eGFR-baseline was not available in all studies. GWAS meta-analyses logistics in so many studies are highly complex; it is not trivial to “add” analyses applying other models. However, there is mathematical help to facilitate covariate adjustment post-hoc, i.e. by formula, based on GWAS summary statistics unadjusted for eGFR-baseline and GWAS summary statistics for eGFR-baseline and study-specific phenotype information[S10]. We demonstrate how this works (**Supplementary Note S1**) and that it works in this setting by validation studies: we compared formula-derived summary statistics for baseline-adjusted decline with model-computed baseline-adjusted decline in a subset of studies (the recently joined CKDGen studies, UK Biobank, selected “old” CKDGen studies). For eGFR-decline adjusted for baseline in the following, we used formula-derived summary statistics for the “old” CKDGen studies and computed summary statistics for the recently joined studies and UK Biobank.

Meta-analyses of GWAS summary statistics

Before meta-analysis, we excluded, from each study file, multi-allelic variants, variants with a Minor Allele Count <10, and variants with an imputation quality <0.6 (R^2 from minimac[S8] or info score from Impute[S7]). Per study, genomic control (GC) correction was applied when the GC-factor lambda was >1. We excluded a study for a specific analysis, when it contributed <100 individuals after quality control for this analysis.

Per model, we conducted a fixed-effects inverse-variance-weighted meta-analysis using metal[S11]. To account for the sequential recruitment of studies, we meta-analyzed per-variant summary statistics across “old” CKDGen studies (GC-corrected) and across recently joined CKDGen studies plus UK Biobank (GC-corrected), and then meta-analyzed these two (again GC-corrected, **Supplementary Figure S1**). After meta-analysis, only variants present in ≥50% of GWAS files and minor allele frequency ≥1% were retained for further analyses.

Identification of associated loci

For our GWAS search, we selected genome-wide significant variants ($P<5.00\times10^{-8}$) in the meta-analyzed summary statistics and identified independent locus lead variants by an iterative approach, as applied previously[S12]: (i) from all genome-wide significant variants, we

selected the variant with the smallest P-value as the first lead variant and defined this variant's locus region as lead variant ±500kB, (ii) omitting this identified region, we selected the next variant with the smallest P-value, and (iii) repeated this procedure until no further variant with P-value<5.00×10^{-8} was observed. The *MHC* region (chr6:28.5-33.5MB) was considered a single locus. We checked for overlapping loci, but there were none.

For the candidate-based approach, we used the 265 lead variants previously reported for association with cross-sectional ln(eGFR)[S12], excluded the locus regions identified by the GWAS search, and, for the remaining candidate variants, judged significance at Bonferroni-corrected level.

For identified variants, we evaluated ancestry-related heterogeneity using MR-MEGA v.0.1.5 (Meta-Regression of MultiEthnic Genetic Association[S13], including three principle components. We also conducted sensitivity analyses incorporating further models of covariate adjustment for identified eGFR-decline associations in a validation meta-analysis.

SNP-by-age interaction on cross-sectional eGFR

We investigated the lead SNPs identified for (creatinine-based) eGFR-decline for SNP-by-age interaction on cross-sectional eGFR (based on creatinine or cystatin C, eGFRcrea, eGFRcys). For this we used data that was independent of the SNP identification step: unrelated European ancestry UK Biobank individuals with one eGFRcrea or eGFRcys assessment excluding the 15,442 individuals in the decline GWAS (yielding > 350,000 individuals).

For each SNP, we applied two linear regression models, one each for the outcome eGFRcrea or eGFRcys, using the covariates age, sex, SNP, SNP-by-age interaction term, and four principal components (age centered at 50 years). We modelled (i) the main age effect on the outcome allowing for non-linear effects (to avoid spurious effects from non-linear main age effect when modelling age linearly), (ii) the main SNP effect linearly per allele dosage, and (iii) for the SNP-by-age interaction effect, the SNP-effect was modelled linearly per allele dosage and the age effect was allowed to vary non-linearly (smooth function, varying coefficient model[S14], penalized thin-plate regression splines, mgcv-package in R[S15]). In a second analysis, the age effect in the SNP-by-age interaction was modelled linearly (i.e. linear effects for both SNP and age in the SNP-by-age term). We judged significance of the interaction at Bonferroni-corrected level.

Genetic effect sizes and GRS analysis for eGFR-decline

We provide SNP-specific effect sizes on eGFR-decline in mL/min/1.73m^2 per year over all individuals and focused on individuals with DM at baseline or CKD at baseline. We provide cumulative effects by GRS analysis in the population-based study HUNT (19-90 years old, European ancestry, up to 21 years of follow-up, mean of age-/sex-adjusted residuals for eGFR-

decline = 1.02 mL/min/1.73m^2/year). To compute the GRS, we counted the number of the faster-decline allele across identified variants for each study participant, weighted by the effect size for eGFR-decline unadjusted for eGFR-baseline, then divided by the sum of weights and multiplied by the number of variants in the GRS. By this, the GRS is scaled from 0 to 2 times the number of variants, where one unit reflects one "average" unfavorable allele. We tested the quantitative GRS with eGFR-decline via linear regression adjusted for age and sex (unadjusted for eGFR-baseline) and we compared individuals with high versus low GRS (≥95th versus ≤5th percentile, ≥90th versus ≤10th percentile derived from UK Biobank excluding individuals in the eGFR-decline GWAS). This was done over all individuals and restricted to individuals with DM at baseline or CKD at baseline.

We also computed a SNP's genetic effect size relative to the phenotype variance as beta-estimates^2 * Var(SNP) / Var(Y), i.e. beta-estimates^2 * 2*MAF*(1-MAF)/ (standard deviation of Y)^2, where MAF is the minor allele frequency of the respective variant. The joint effect of several variants was derived as the sum of the respective SNPs' effects. For this, again, we used the phenotype variance from HUNT: the standard deviation of age-/sex-adjusted residuals for eGFR-decline = 0.91 mL/min/1.73m^2 overall, 1.25 mL/min/1.73m^2 among individuals with DM, 1.39 mL/min/1.73m^2 with CKD, and for eGFR cross-sectional =0.12 mL/min/1.73m^2 on the log-scale.

GRS analyses for ESKD and AKI

We were interested in whether the GRS across the variants identified for eGFR-decline showed association with severe clinical endpoints, ESKD and AKI. For this, we used three case sets for ESKD and one case set for AKI as well as controls (eGFR>60 mL/min/1.73m^2) from population-based studies frequency-matched with regard to age-group and sex as described previously[S16]. Briefly, the three ESKD studies consisted of: (i) ESKD cases from unrelated European ancestry UK Biobank individuals (ICD10 code N18.0 or N18.5, i.e. need for dialysis) and matched UK Biobank controls (no record of any N18), excluding individuals in eGFR-decline GWAS; (ii) ESKD cases from GENDIAN and controls from KORA-F4; (iii) ESKD cases from the 4D-study[S17] and controls from KORA-F3. The study on AKI used AKI cases from UK Biobank (ICD10 code N17, "Acute Renal Failure") and UK Biobank controls (no record of N17), excluding individuals in eGFR-decline GWAS. By this, the cases and controls across all four studies were independent of eGFR-decline GWAS, except the KORA-F3 and KORA-F4 controls to keep the previously designed and published case-control comparisons with GENDIAN and 4D.

For each of these four case-control studies, we retrieved the respective SNPs and computed a weighted GRS across identified variants for each individual as described above. We tested the quantitative GRS with ESKD or AKI. We applied a one-sided test, since we were

only interested in this association when the GRS increased the odds of ESKD or AKI. We also compared individuals with high versus low GRS (≥95th GRS percentile, ≤5th percentile and ≥90th versus ≤10th GRS percentile, defined in UK Biobank individuals excluding individuals in eGFR-decline GWAS) and tested (one-sided) for increased odds of ESKD (meta-analysis across the three studies) or AKI. Associations are derived via logistic regression adjusted for matching variables age-groups and sex (for AKI additionally for the first two principal components).

Supplementary Note S1: Equivalence of DM-adjusted versus not DM-adjusted GWAS on eGFR-decline in the validation meta-analysis

In the recently joined studies (HUNT, MGI, AugUR) and UK Biobank, we had more adjustment models computed for GWAS on eGFR-decline, to better understand similarities and differences. In these, we compared the GWAS summary statistics for eGFR-decline adjusted for DM-status to GWAS without adjustment for DM-status (i.e. GWAS on age- and sex-adjusted residuals and with and without adjustment for DM-status at baseline). In each study, we found precisely the same beta-estimates and standard errors (SE): (i) for the 265 SNPs identified previously for cross-sectional eGFR[S12], for which we had a prior hypothesis that these contained the SNPs associated with eGFR-decline, as well as (ii) genome-wide where most of the SNP-associations are under the Null (**Supplementary Figure S4A**).

We added further “old” CKDGen studies to substantiate these findings in further studies and in an expanded validation meta-analysis (n=103,970). Again, we found DM-adjusted and not DM-adjusted beta-estimates and SEs to be precisely the same (**Supplementary Figure S4A**). Of note, this validation meta-analysis included general population studies and studies of specific scope: hospital-based (MGI), focused on individuals aged 70+ years (AugUR), or focused on individuals with chronic kidney disease (GCKD).

Given this equivalence, we did not distinguish any more between results DM-unadjusted or DM-adjusted.

Supplementary Note S2: Formula-based covariate adjustment using GWAS summary statistics

Let's assume we have a quantitative phenotype Y and a covariate C. Let's further assume, we have GWAS summary statistics as beta-estimates and respective standard errors, $\hat{\beta}_Y$ and $\widehat{SE}_Y$ (beta-estimate and standard error) from linear regression models per genetic variant, i.e. from $Y \sim \alpha + \beta_Y SNP$ (unadjusted model, omitting the indexing per variant). Let's assume we also have GWAS summary statistic $\hat{\beta}_C$ and $\widehat{SE}_C$ for the covariate C from the model $C \sim \alpha + \beta_C SNP$ (covariate model via linear regression, C binary or quantitative). We can then "adjust" the summary statistics formula-based, i.e. we can derive the GWAS summary statistics $\hat{\beta}_{YadjC}$ and $\widehat{SE}_{YadjC}$ for the adjusted model, $Y_{adjC} \sim \alpha + \beta_{YadjC} SNP + yC$, as described[S18] according to

$$\hat{\beta}_{YadjC} = \hat{\beta}_Y - \left(r_{YC} * \frac{sd_Y}{sd_C}\right) * \hat{\beta}_C \text{ and}$$

$$\widehat{SE}_{YadjC} = \sqrt{\widehat{SE}_Y^2 + \left(r_{YC} * \frac{sd_Y}{sd_C}\right)^2 * \widehat{SE}_C^2 - 2 * \left(r_{YZ} * \frac{sd_Y}{sd_C}\right) * corr\left(\hat{\beta}_Y, \hat{\beta}_C\right) * \widehat{SE}_Y * \widehat{SE}_C}.$$

Here, we assume that we know the standard deviation of C and Y, sd_C and sd_Y, respectively, the phenotypic correlation, r_{YC} (estimated as Pearson correlation coefficient between Y and C) and the genetic correlation between Y and C, $corr\left(\hat{\beta}_Y, \hat{\beta}_C\right)$, (using all genetic effects for Y and C genome-wide for estimation as reasonable proxy). When r_{YC} is zero, the adjusted model SNP-effects, $\hat{\beta}_{YadjC}$, are the same as the unadjusted model SNP-effects, $\hat{\beta}_Y$.

Alternatively, when we have GWAS summary statistics from the adjusted model, $Y_{adjC} \sim \alpha + \beta_{YadjC} SNP + yC,$ and the covariate model, $C \sim \alpha + \beta_C SNP$, we can "de-adjust" summary statistics formula-based, i.e. we can derive the GWAS summary statistics of the unadjusted model as

$$\hat{\beta}_Y = \hat{\beta}_{YadjC} + \left(r_{YC} * \frac{sd_Y}{sd_C}\right) * \hat{\beta}_C \text{ and}$$

$$\widehat{SE}_Y = \sqrt{\widehat{SE}_{YadjC}^2 + \left(r_{YC} * \frac{sd_Y}{sd_C}\right)^2 * \widehat{SE}_C^2 + 2 * \left(r_{YZ} * \frac{sd_Y}{sd_C}\right) * corr\left(\hat{\beta}_{YadjC}, \hat{\beta}_C\right) * \widehat{SE}_{YadjC} * \widehat{SE}_C}$$

.

We apply this on our example to summary statistics for annual eGFR-decline adjusted for eGFR-baseline (BL): given the beta-estimates for decline unadjusted for $\ln(\text{eGFRcrea}_{BL})$ (in fact, residuals adjusted for age, sex), $\hat{\beta}_{decline}$, and the beta-estimates for $\ln(\text{eGFRcrea}_{BL})$ (i.e. residuals adjusted for age and sex), $\hat{\beta}_{BL}$, we can "adjust" results for BL using the formula, i.e., derive the beta-estimates for decline adjusted for BL (residuals adjusted for age and sex), $\hat{\beta}_{decline\_adj\_BL}$, as

$$\hat{\beta}_{decline\_adj\_BL} = \hat{\beta}_{decline} - \left(r_{decline,BL} * \frac{sd_{decline}}{sd_{BL}}\right) * \hat{\beta}_{BL}.$$

Effect sizes here are given for the BL-lowering effect allele (which is usually the decline-increasing allele). The can also be written as

$$\frac{\hat{\beta}_{decline\_adj\_BL}}{sd_{decline}} = \frac{\hat{\beta}_{decline}}{sd_{decline}} + r_{decline,BL} * \left(-\frac{\hat{\beta}_{BL}}{sd_{BL}}\right).$$

This shows that the effect size of decline adjusted for BL standardized to the scale of standardized $\hat{\beta}_Y$ effects (i.e. divided by $sd_{decline}$) is the sum of (i) the (standardized) effect size of decline unadjusted (i.e. the vertical distance of this effect to the x-axis in a $\hat{\beta}_Y/sd_Y$ versus $\hat{\beta}_C/sd_C$ plane) and (ii) the vertical distance from the intersection point of the x-axis at $\hat{\beta}_C/sd_C$ (i.e. < 0 when the coding allele is the $\hat{\beta}_C$-lowering allele) to the phenotype correlation line, $f(x) = r_{YC} * x$, when the phenotype correlation is positive, like r_{YC}=0.33 in UK Biobank, i.e. to the point ($\hat{\beta}_C/sd_C$, 0.33*$\hat{\beta}_C/sd_C$). This also shows that $\hat{\beta}_{decline_{adj_{BL}}} < \hat{\beta}_{decline}$, since $\hat{\beta}_C < 0$, by definition.

Supplementary Note S3: Validation of the formula-derived association for eGFR-decline adjusted for eGFR-baseline

In the recently joined studies and UK Biobank, we had more adjustment models computed for GWAS on eGFR-decline, to better understand similarities and differences. In these, we compared the summary statistics for eGFR-decline adjusted for eGFR-baseline (i.e. age- and sex-adjusted residuals and additional adjusted for ln(eGFRcrea baseline)) with eGFR-decline unadjusted for eGFR-baseline (i.e. age- and sex-adjusted residuals) and found substantial differences (**Supplementary Figure S4B**). Thus, the two models, unadjusted and adjusted for eGFR-decline were considered further.

Generally, in GWAS meta-analysis, the number of GWAS models computed needs to be as parsimonious as possible to remain feasible. In each of the "old" CKDGen studies, we had GWAS summary statistics for eGFR-decline unadjusted for eGFR-baseline, GWAS summary statistics for cross-sectional eGFR, and study-specific phenotypic information. We knew that this enabled us to do the adjustment by formula[S10,S18] (**Supplementary Note S1**). For the "old" CKDGen studies, we thus derived GWAS summary statistics for eGFR-decline adjusted for eGFR-baseline applying this formula.

While the formula was established previously[S10], we validated that it worked in this setting using the recently joined CKDGen studies and UK Biobank, where we had the model "eGFR-decline adjusted for eGFR-baseline" computed: we also derived the SNP-associations for "eGFR-decline adjusted for eGFR-baseline" based on the formula for comparison in these studies for the purpose of validation. We found the formula to work very precisely per study: we observed equivalence in beta estimates and SEs when focused on the 265 SNPs identified previously for cross-sectional eGFR[S12], for which we had a prior hypothesis that these contained the SNPs associated with eGFR-decline, as well as genome-wide, where most SNP-

associations were under the Null (**Supplementary Figure S4C**; e.g., in UK Biobank for the 265 variants: Pearson correlation coefficient r=1.00 for betas and SEs; maximum difference in beta=3.26x10^{-2}, maximum differences in SEs =1.01x10^{-3}). We added further “old” CKDGen studies also to yield an expanded validation meta-analysis (n=103,970). Again, we found the formula to work precisely in each study and in the expanded validation meta-analysis (**Supplementary Figure S4C**).

The formula is mathematically derived and works perfectly when GWAS summary statistics for baseline eGFR are available. For studies with GWAS on cross-sectional eGFR, the sample size for cross-sectional eGFR is typical a bit larger than the sample size for eGFR-baseline for longitudinal studies (i.e. restricting to individuals in the follow-up). We evaluated the impact of using cross-sectional eGFR summary statistics rather than baseline eGFR summary statistics in the formula in three “old” CKDGen studies at the hand of the Regensburg meta-analysis center. There was no difference in SEs for the 265 variants or genome-wide, a slight difference for beta estimates of the 265 variants, and a larger (random, not biased) difference in betas genome-wide (**Supplementary Figure S4D**). This difference in genome-wide SNP-estimates can be attributed to random noise in the per-variant estimates under the null hypothesis (considering most genome-wide SNPs as not associated with eGFR-decline). We extended this validation experiment by three further studies, and found the same (**Supplementary Figure S4D**). In summary, we concluded that the formula-derived association estimates worked well in this setting for the 265 variants and also, with some more random noise, for the other genome-wide variants.

Of note, these validation meta-analyses included general population studies as well as studies of specific scope: hospital-based (MGI), focused on individuals aged 70+ years (AugUR), focused on individuals with chronic kidney disease (GCKD), or focused on individuals with DM (Diacore).

Supplementary Note S4: Graphical illustration of the relationship between SNP-effects on eGFR-decline unadjusted and adjusted for eGFR-baseline.

Figure 2C provides an informative geometrical illustration for the relationship between a SNP-effect on eGFR-decline baseline-unadjusted (standardized, depicted on Y-axis), $\hat{\beta}_{DECLINE}/sd_{DECLINE}$, and the SNP-effect on eGFR-decline baseline-adjusted (standardized to Y-axis scale), $\hat{\beta}_{DECLINE\_adj\_BL}/sd_{DECLINE} = \hat{\beta}_{DECLINE}/sd_{DECLINE} + r_{decline,BL} * \left(-\hat{\beta}_{BL}/sd_{BL}\right)$, where $r_{DECLINE,BL}$ is the phenotypic correlation of baseline-unadjusted eGFR-decline with baseline eGFR and $\hat{\beta}_{BL}/sd_{BL}$ is the standardized variant effect on baseline eGFR.

While this relationship was derived per study (**Supplementary Note S1**), this also holds approximately for meta-analyzed effect sizes, as mostly the same studies contributed to the respective meta-analyses. The difference between the two effects, baseline-adjusted and

baseline-unadjusted decline, $r_{decline,BL} * (-\hat{\beta}_{BL}/sd_{BL})$, can be visualized when adding the phenotype correlation line, $f(x) = r_{DECLINE,BL} * x$ (mean correlation across studies= 0.34): while the baseline-unadjusted decline effect, $\hat{\beta}_{DECLINE}/sd_{DECLINE}$, is the vertical distance from symbol to X-axis, the baseline-adjusted decline effect, $\hat{\beta}_{DECLINE\_adj\_BL}/sd_{DECLINE}$, is the vertical distance from symbol to phenotype correlation line.

Supplementary Note S5: Comparison of the signals for eGFR-decline unadjusted and adjusted for eGFR-baseline and cross-sectional eGFR for the 11 identified loci

We compared the association signals for the 11 identified loci for eGFR-decline (unadjusted for eGFR-baseline) with signals for eGFR-decline adjusted for eGFR-baseline with signals for eGFR cross-sectional[S12] in regional association plots (**Supplementary Figure S5A-C**),

For the 4 variants identified for eGFR-decline unadjusted for eGFR-baseline, we found unadjusted eGFR-decline signals to coincide with adjusted eGFR-decline signals and with cross-sectional eGFR signals (**Supplementary Figure S5A**). Lead variants for unadjusted eGFR-decline (i.e. the variant with the smallest P-value for unadjusted eGFR-decline) were the same or highly correlated with the respective cross-sectional lead variants (r^2=same, same, 1.00 and 0.93 for *UMOD-PDILT* (2), *PRKAG2* and *SPATA7*, respectively).

Among the 5 lead variants identified by GWAS on eGFR-decline adjusted for eGFR-baseline with significant association for eGFR-decline unadjusted for eGFR-baseline (i.e. "genuine" eGFR-decline variants, **Supplementary Figure S5B**), all signals for decline adjusted coincided with respective signals for decline unadjusted, except for the *TPPP* locus (but there, the signal for decline unadjusted sharpened when including the studies with lower imputation quality and then coincided). Three of the 5 lead variants were the same as (*FGF5*) or highly correlated with (*C15ORF54* and *ACVR2B,* R^2=0.61 and 0.98) the respective lead variants for decline unadjusted. In the *OVOL1* locus, the lead variant for decline adjusted (rs4930319) depicted the same association signal as for decline unadjusted, but was not highly correlated with the variant with the smallest P-value for decline unadjusted (R^2 with rs117829045=0.11) due to differing allele frequencies (MAF=0.11 and 0.33, respectively); the variants were suggested to be inherited via the same haplotypes (D'=1.00). Among the 5 variants, we found 3 signals for eGFR-decline adjusted for eGFR-baseline to coincide with the signal for cross-sectional eGFR (for *FGF5*, *OVOL1*, *ACVR2B)* and lead variants for decline adjusted as highly correlated with the respective lead variants for cross-sectional eGFR (r^2= 0.95, 0.98, 0.96, respectively; **Supplementary Figure S5B**). In *C15ORF54* and *TPPP* loci, the decline adjusted signal appeared to be a 2nd signal for cross-sectional eGFR: the lead variant for decline adjusted were not correlated with the lead variant for cross-sectional eGFR (R^2= 0.04 and 0.11). The lead variant for decline adjusted near *TPPP* depicted a cross-sectional signal 22kb distant from the reported cross-sectional lead variant with different allele

frequencies (MAF=0.49 and 0.27, respectively; D'=0.57); of note, the lead variants for decline adjusted captured a 2nd signal identified in the recently published cross-sectional eGFR analysis[S19] and there the lead variants were exactly the same. The *C15ORF54* lead variant for decline adjusted was highly correlated with a 2nd signal for cross-sectional eGFR (rs28833881, r^2=0.98).

For the 3 loci identified by eGFR-decline adjusted for eGFR-baseline without significant association with eGFR-decline unadjusted for eGFR-baseline (i.e., not a genuine eGFR-decline association), there was no signal for decline unadjusted (*GATM*, *CPS1*, *SHROOM3*; **Supplementary Figure S5C**). The lead variants for decline adjusted were the same or highly correlated with the respective cross-sectional eGFR lead variant (R^2=0.98, same, 0.59).

Supplementary Note S6: Age-dependency of SNP-effects and main age effect on eGFR.

Before interpreting SNP-by-age interaction effects on cross-sectional eGFRcrea and eGFRcys, we evaluated the main age effect on eGFRcrea and eGFRcys (i.e. age and sex in the model). We found large main age effects, which were fairly linear: beta-estimate per year of age [95%-CI] = -0.775 units [-0.780, -0.771] and -1.024, [-1.030, -1.019] on eGFRcrea or eGFRcys, respectively (**Supplementary Figure S6Z**). We nevertheless allowed for non-linear main age effects in the SNP-by-age interaction analyses, since the main age effect was large and even a slight deviation from non-linearity can distort interaction effects if unaccounted.

We found the age-dependency of the SNP-effects on eGFRcrea and eGFRcys (i.e. age-effect in the interaction term) to be fairly linear when non-linear modelling of main age effect was applied (**Supplementary Figure 6 SA,B,C**). Of note, when the main age effect was modelled linearly, the SNP-effects on eGFRcrea and eGFRcys appeared to be non-linearly modified by age, which is a known problem in interaction analyses (data not shown); this supported the choice of the main age effect modelled non-linearly.

Supplementary Note S7: Narrow-sense heritability

We estimated SNP-based heritability (h^2) for eGFR-baseline and for eGFR-decline unadjusted and adjusted for eGFR-baseline using the genomic relatedness matrix restricted maximum likelihood (GREML) method as implemented in the GCTA software package (https://yanglab.westlake.edu.cn/software/gcta/#Overview). For this, we used individual participant data from UK Biobank for the ~15,000 unrelated individuals of European ancestry that had baseline and follow-up eGFR measurements available.

We estimated narrow-sense heritability (h^2) for eGFR-decline at 1% (standard error 2%, P = 0.31) and 5% for eGFR-decline adjusted for baseline (standard error 2.1%, P = 0.0075) and 20% (standard error 2.5%, P< 1.00×10^{-100}) for eGFR-baseline.

The small heritability for eGFR-decline in UK Biobank might derive from a large measurement error in eGFR-decline based on a study with only two measurements only 4 years apart. The larger heritability for eGFR-decline adjusted for eGFR-baseline compared to unadjusted for eGFR-baseline is reflective of the collider bias.

Supplementary Figure S1: Meta-analysis workflow. Shown is the meta-analysis workflow to capture the sequential recruitment and different suite of computed models (eGFR-decline unadjusted and adjusted for eGFR-baseline, “decline” and “decline adjusted”). In the first level, we conducted a meta-analysis of summary statistics across studies that were part of CKDGen since a long time (“old CKDGen studies”, green boxes) and a meta-analysis across recently joined CKDGen studies (“new studies”, blue boxes) and UK Biobank (orange box). In a second level, we meta-analyzed these two results. At each level, genomic-control (GC) correction was applied, when lambda was >1.00.

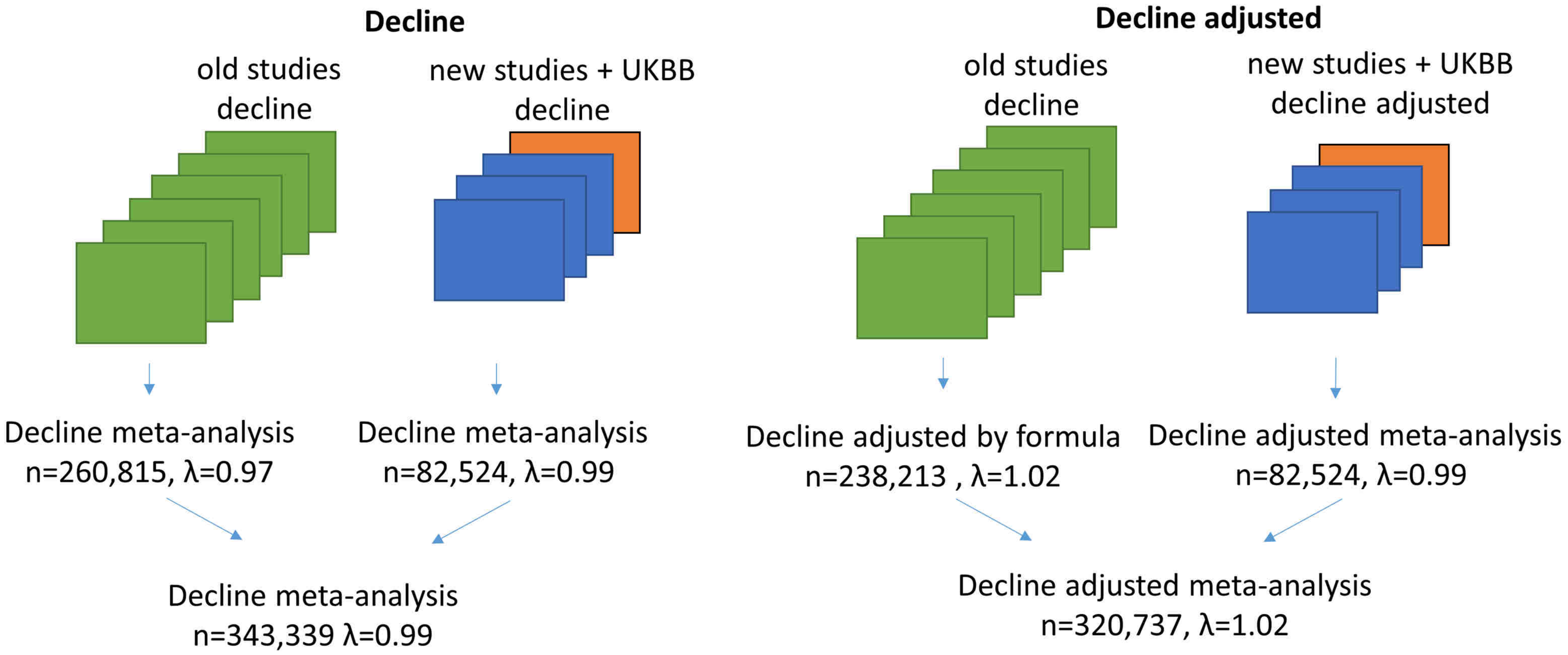

Supplementary Figure S2: Study-specific median annual eGFR-decline versus sample size, follow-up time and median age. Shown are, for each of the 62 studies, the study-specific median of annual eGFR-decline versus **(A)** number of individuals, **(B)** time to follow-up, and **(C)** median age at baseline. Whiskers represent interquartile range.

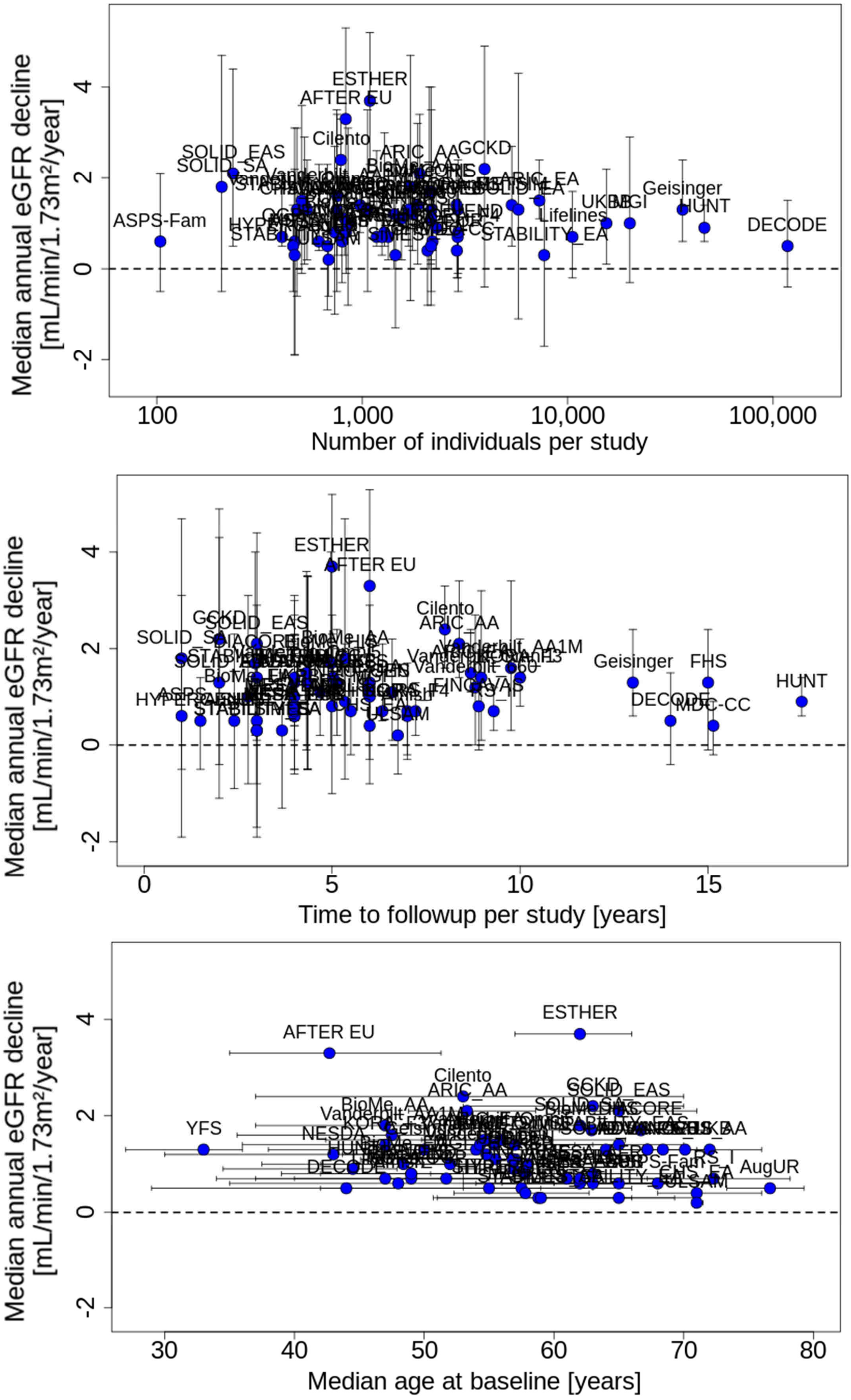

Supplementary Figure S3: No influence of alternative adjustments for age on eGFR-decline in UK Biobank. We explored alternative adjustments for age in UK Biobank (n=15,442, age range 40-70 years): **(A)** residuals of eGFR-decline adjusted for age, sex, and ln(eGFR-baseline) versus residuals of eGFR-decline adjusted for age, sex and residuals (ln(eGFR-baseline) adjusted for age and sex) and **(B)** residuals of eGFR-decline adjusted for age_centered (i.e. centered at 50 years) and sex with residuals of eGFR-decline adjusted for age_centered, (age_centered)² and sex. Alternative adjustments did not change the GWAS phenotype.

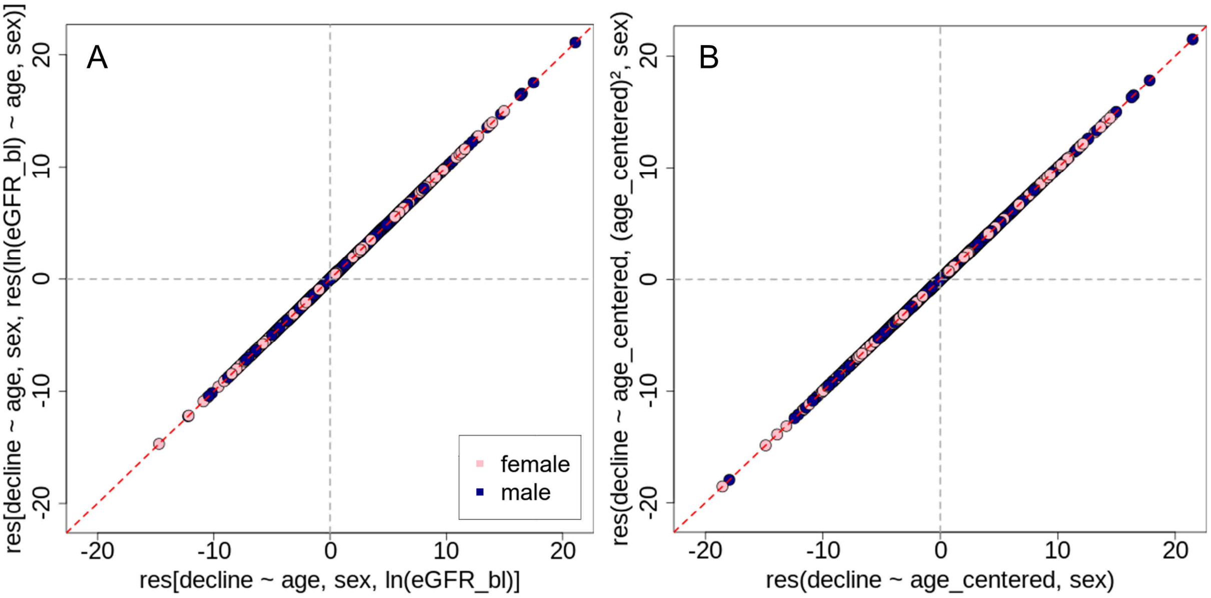

Supplementary Figure S4A: No influence from adjusting SNP-associations for eGFR-decline for diabetes mellitus (DM). We compared SNP-associations for eGFR-decline with DM-adjustment with SNP-associations for eGFR-decline without adjustment for DM in recently joined CKDGen studies, UK Biobank, several "old CKDGen studies", and their meta-analysis (total=103,970; **Supplementary Note S2**). Columns 1&2 show beta-estimates and standard errors (SE) among the 265 variants known for cross-sectional eGFR[S12], where we had a prior hypothesis that these might be associated with eGFR-decline. Columns 3&4 show betas and SEs genome-wide, where most SNP-associations are under the Null (i.e., not associated with eGFR-decline). Column 5 shows QQ-plots for P-values genome-wide. Coded allele is the cross-sectional eGFR-lowering allele, SNPs with minor allele frequency ≥0.05 are in green and with minor allele frequency <0.05 in orange. All SNPs have imputation quality>0.6 and MAC>10 for each study.

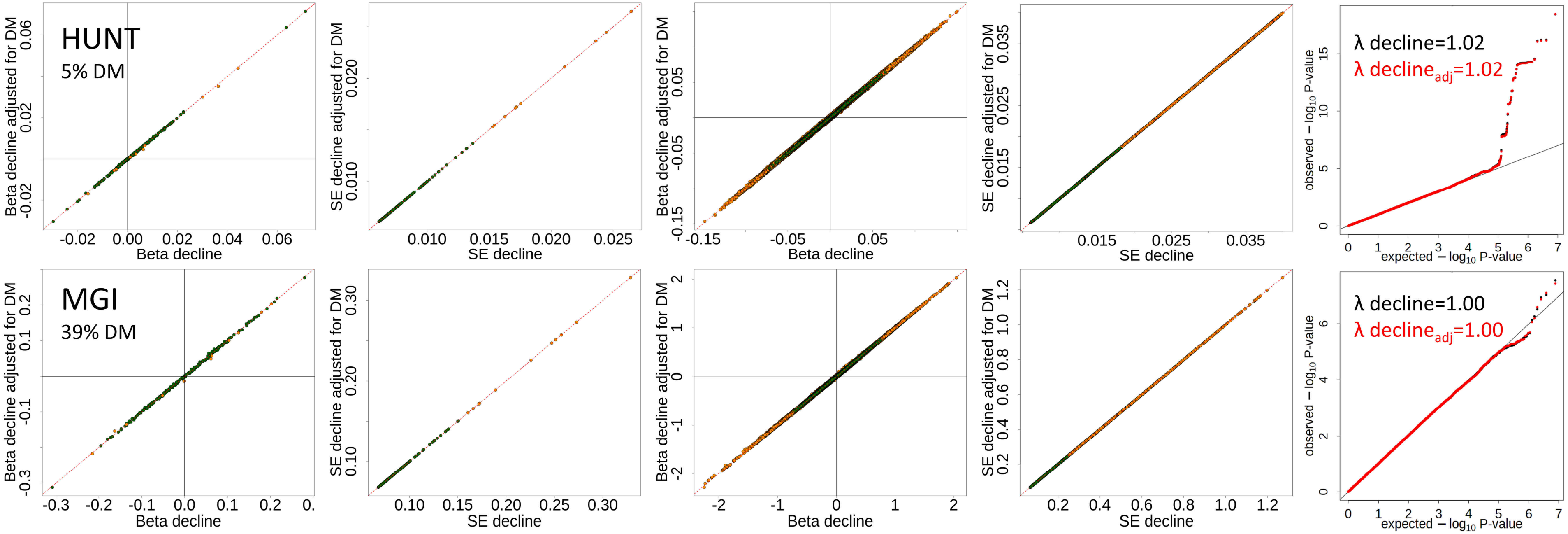

Supplementary Figure S4A: continued

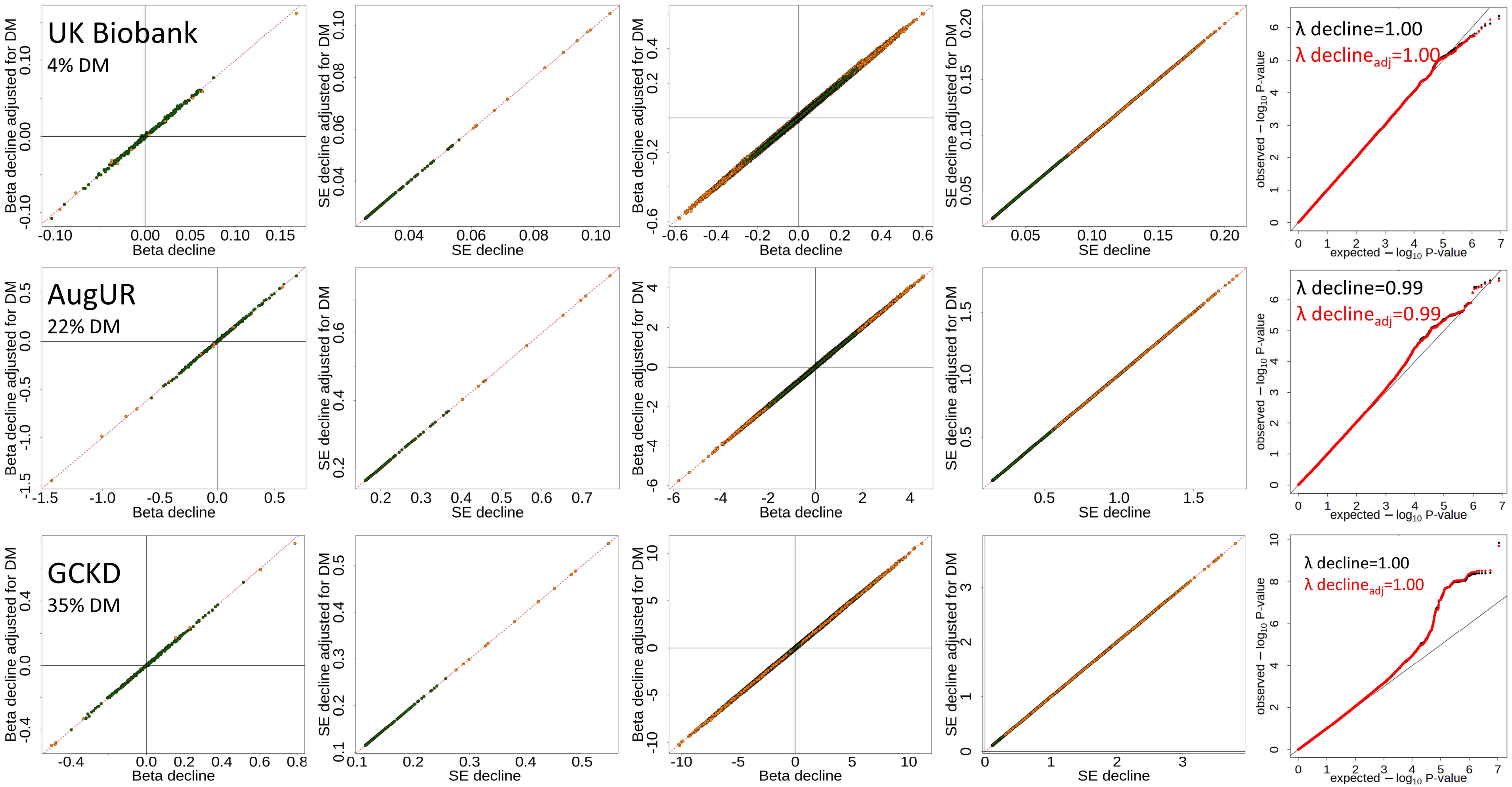

Supplementary Figure S4A: continued

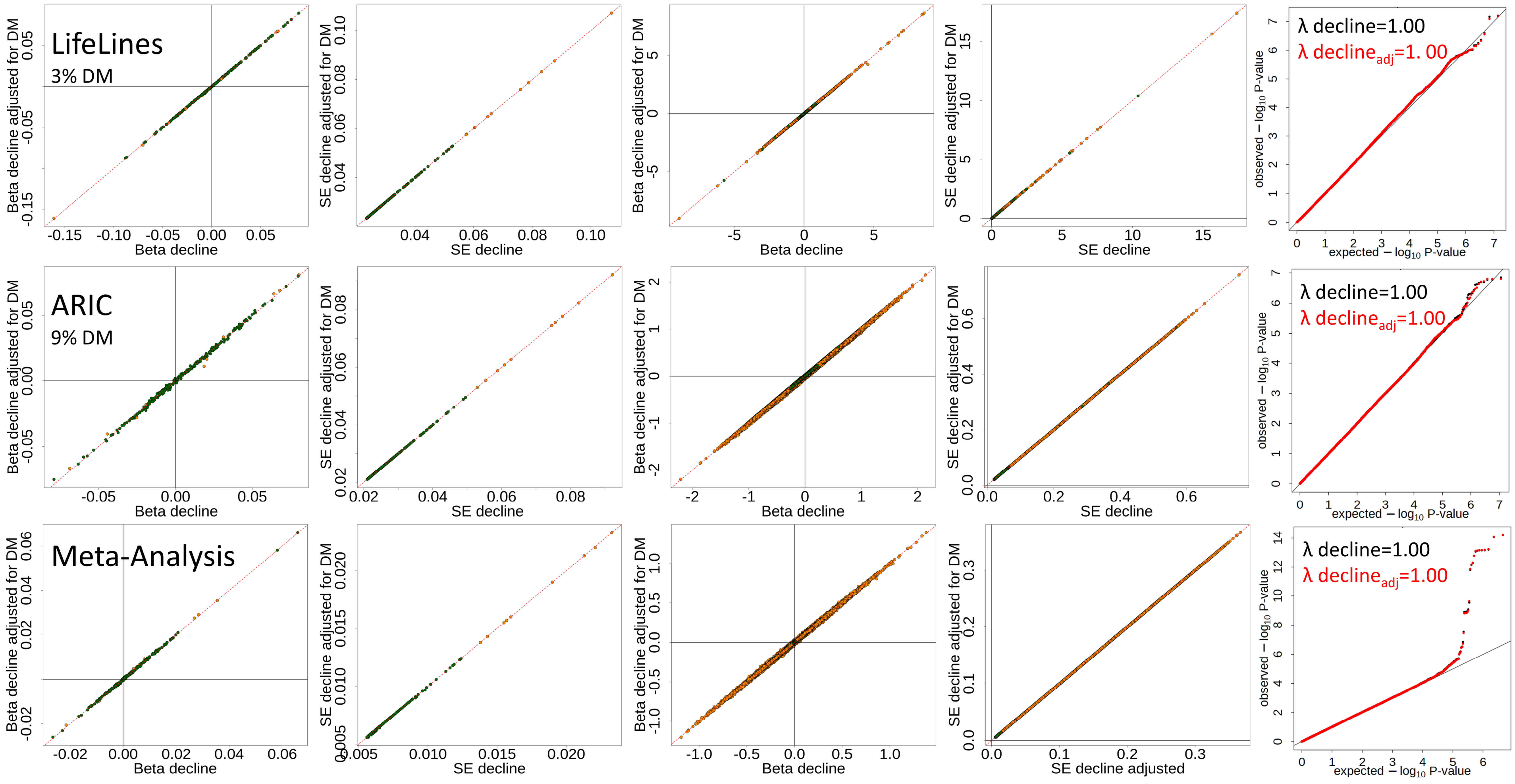

Supplementary Figure S4B: Differences between SNP-association for eGFR-decline unadjusted versus adjusted for eGFR-baseline We compared SNP-associations for eGFR-decline adjusted for eGFR-baseline with SNP-associations for eGFR-decline unadjusted for eGFR-baseline in recently joined studies, UK Biobank, several "old CKDGen studies", and their meta-analysis (total=103,970). Columns 1&2 show beta-estimates and standard errors (SE) among the 265 variants known for cross-sectional eGFR[S12], where we had a prior hypothesis that these might be associated with eGFR-decline. Columns 3&4 show betas and SEs genome-wide, where most SNP-associations are under the Null (i.e., not associated with eGFR-decline). Column 5 shows QQ-plots for P-values genome-wide. Coded allele is the cross-sectional eGFR- lowering allele, SNPs with minor allele frequency ≥0.05 are in green and with minor allele frequency <0.05 in orange. All SNPs have imputation quality>0.6 and MAC>10 for all studies.

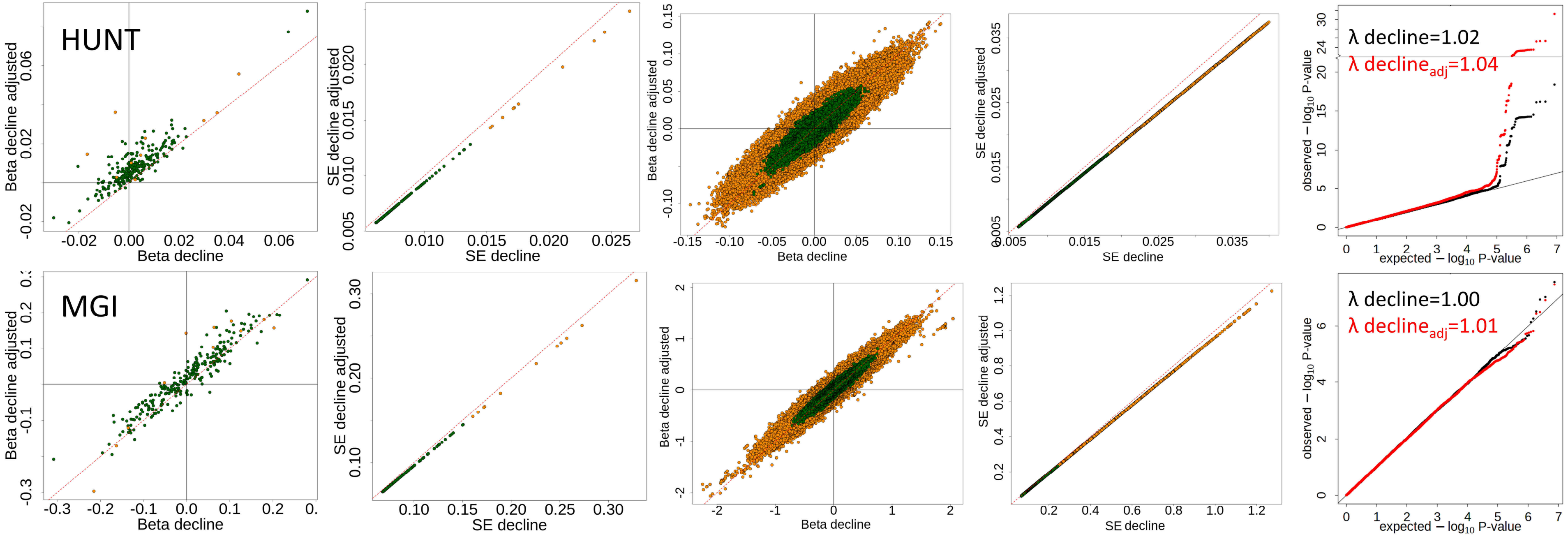

Supplementary Figure S4B: continued

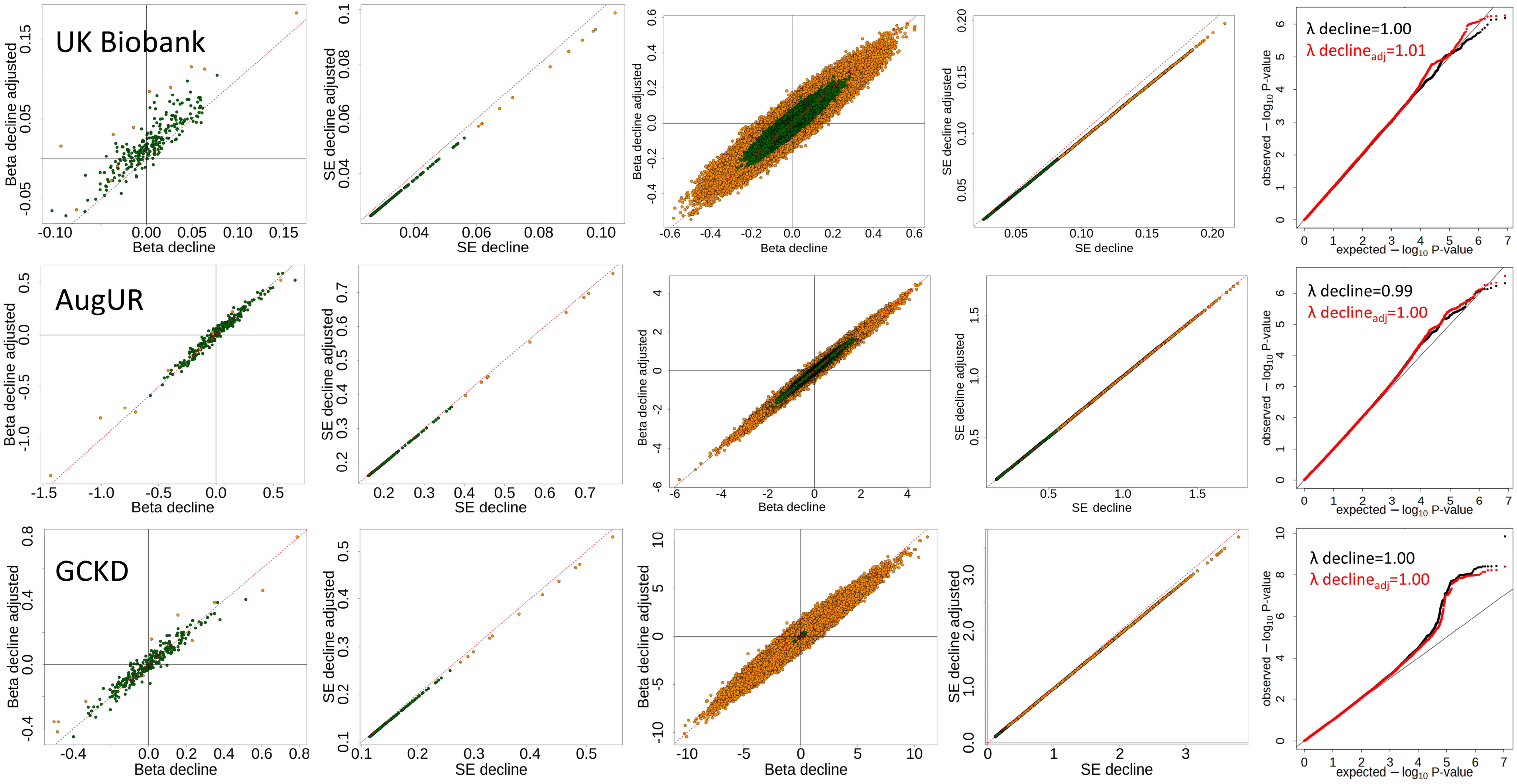

Supplementary Figure S4B: continued

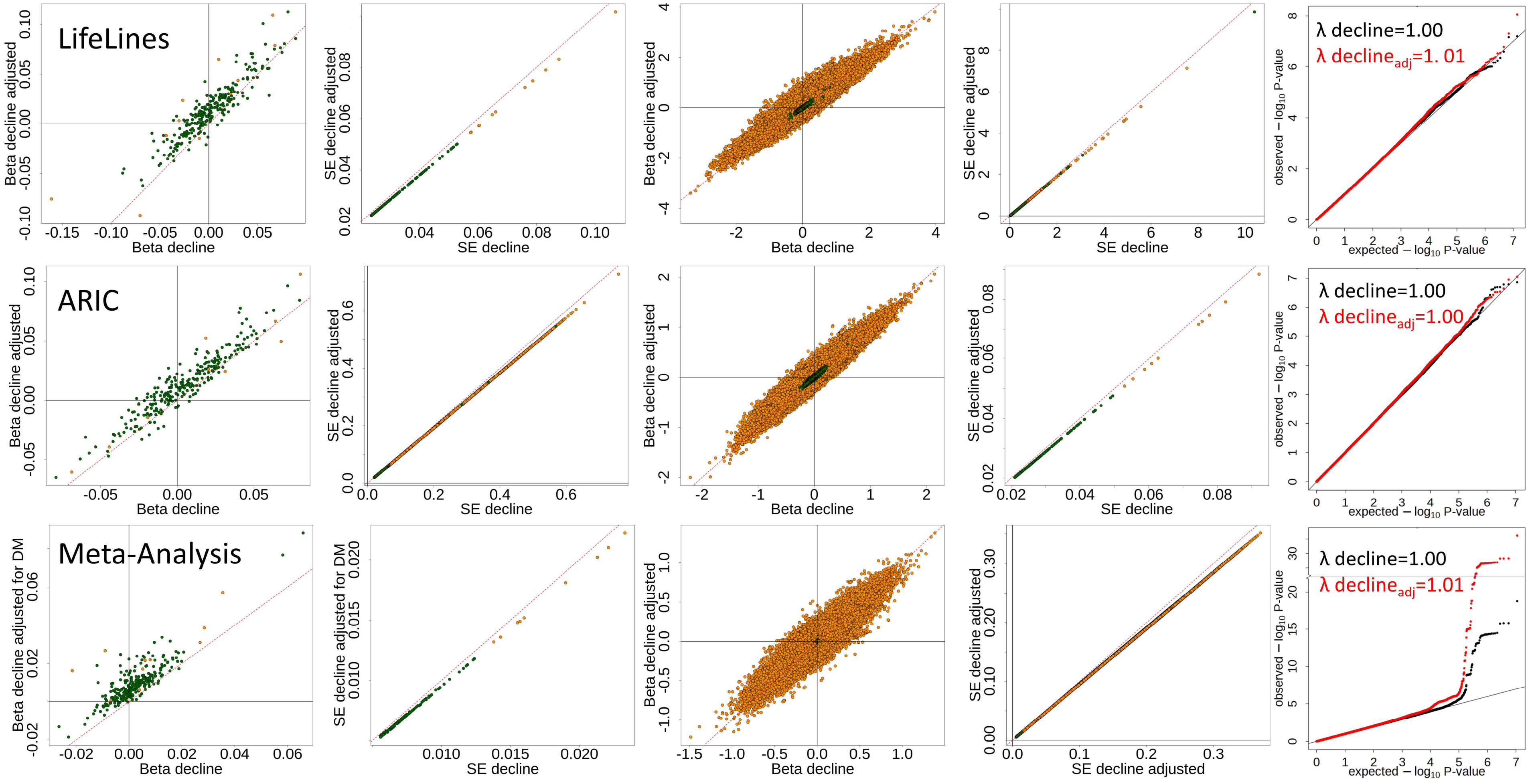

Supplementary Figure S4C: Validation of formula-derived adjustment for eGFR-baseline in eGFR-decline associations (part 1). We compared SNP-associations for eGFR-decline adjusted for eGFR-baseline by model with SNP-associations for eGFR-decline adjusted for eGFR-baseline by formula (using beta-estimates for eGFR-baseline) in recently joined studies, UK Biobank, several "old CKDGen studies", and their meta-analysis (total=103,970). Columns 1&2 show beta-estimates and standard errors (SE) among the 265 variants known for cross-sectional eGFR[S12], where we had a prior hypothesis that these might be associated with eGFR-decline. Columns 3&4 show betas and SEs genome-wide, where most SNP-associations are under the Null (i.e., not associated with eGFR-decline). Column 5 shows QQ-plots for P-values genome-wide. Coded allele is the cross-sectional eGFR-lowering allele, SNPs with minor allele frequency ≥0.05 are in green and with minor allele frequency <0.05 in orange. All SNPs have imputation quality>0.6 and MAC>10 for all studies.

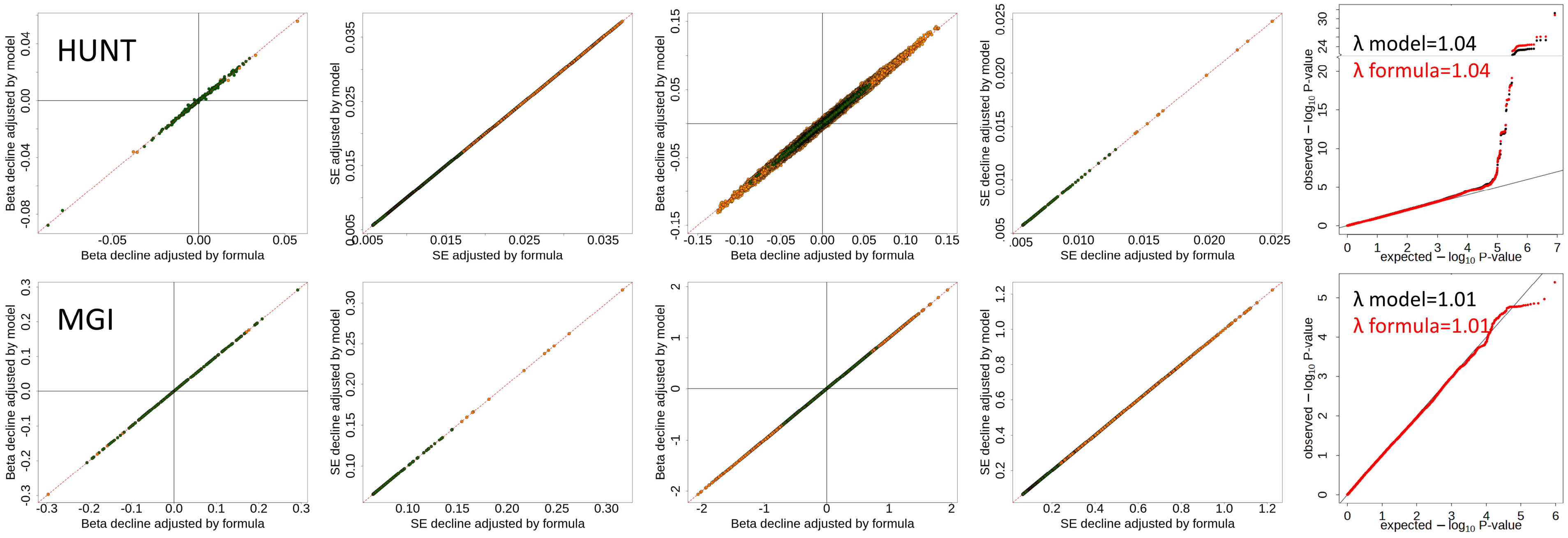

Supplementary Figure S4C: continued

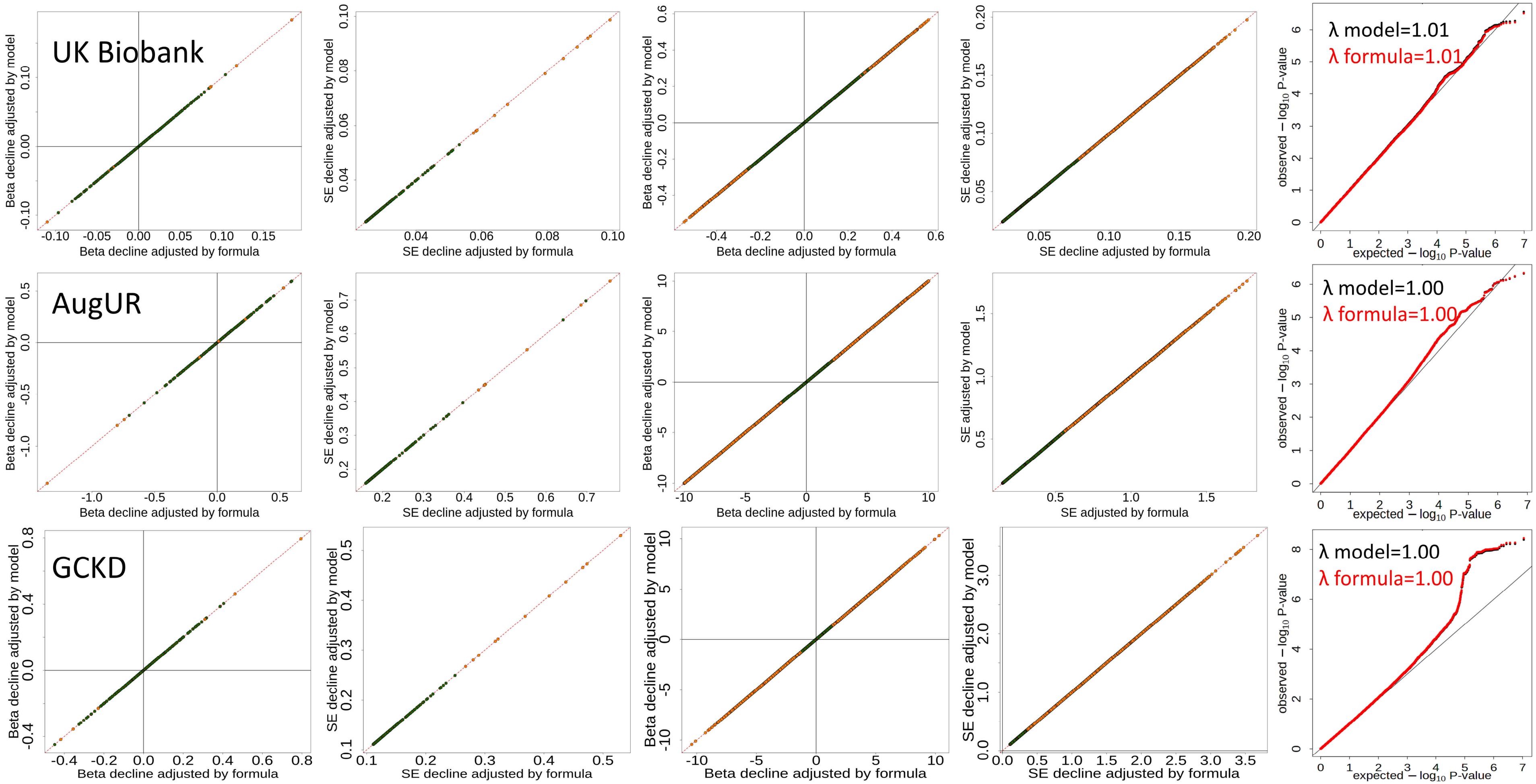

Supplementary Figure S4C: continued

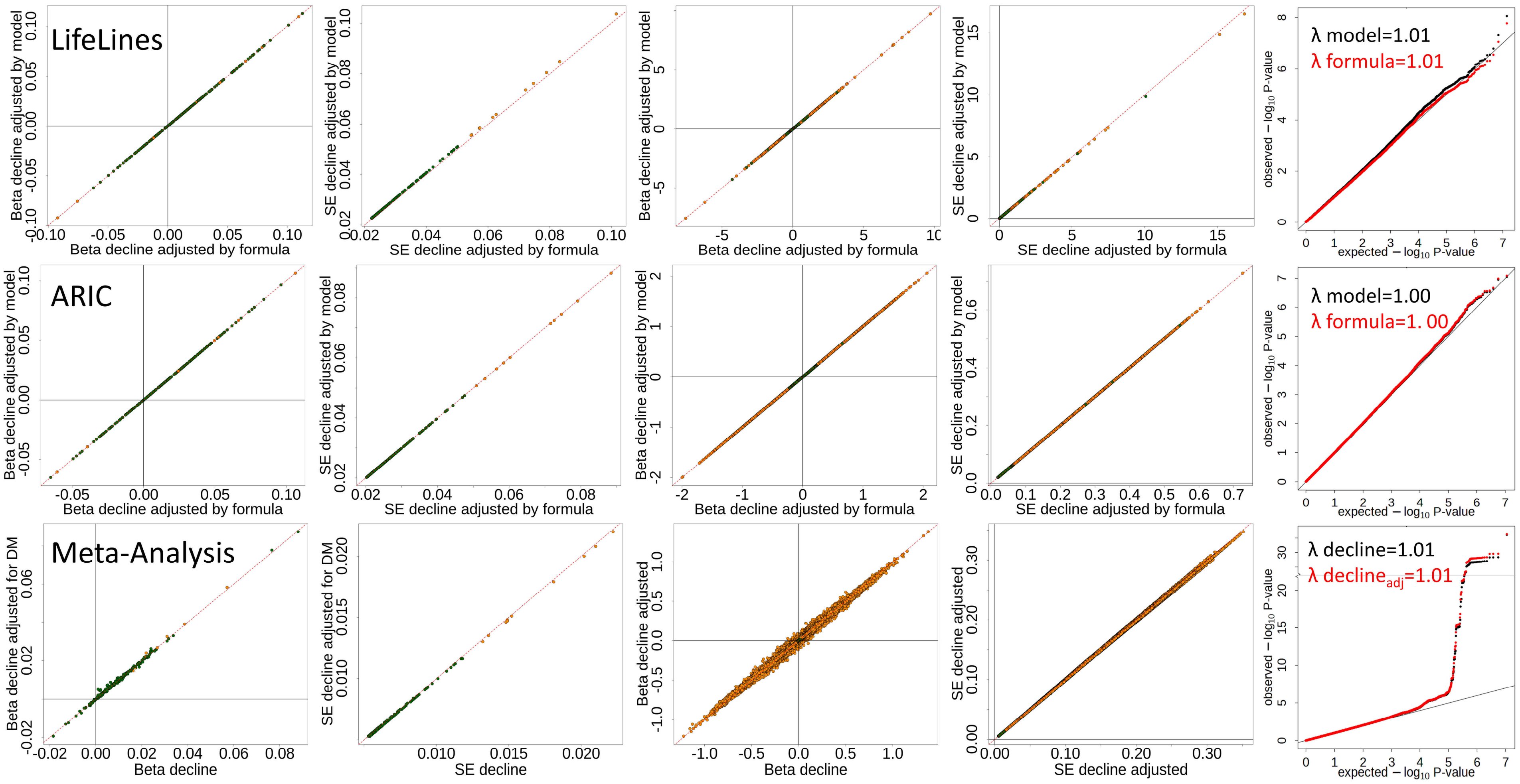

Supplementary Figure S4D: Validation of formula-derived adjustment for eGFR-baseline in eGFR-decline associations (part 2). In "old CKDGen studies", sample sizes were typically larger for cross-sectional eGFR than for baseline eGFR (i.e. restricted to individuals in follow-up). We compared SNP-associations for eGFR-decline adjusted for eGFR-baseline by model with SNP-associations for eGFR-decline adjusted for eGFR-baseline by formula using beta-estimates for cross-sectional eGFR in six "old" CKDGen studies. Columns 1&2 show beta-estimates and standard errors (SE) among the 265 variants known for cross-sectional eGFR[S12], where we had a prior hypothesis that these might be associated with eGFR-decline. Columns 3&4 show betas and SEs genome-wide, where most SNP-associations are under the Null (i.e., not associated with eGFR-decline). Column 5 shows QQ-plots for P-values genome-wide. Coded allele is the cross-sectional eGFR-lowering allele, SNPs with minor allele frequency ≥0.05 are in green and with minor allele frequency <0.05 in orange. All SNPs have imputation quality>0.6 and MAC>10 for all studies.

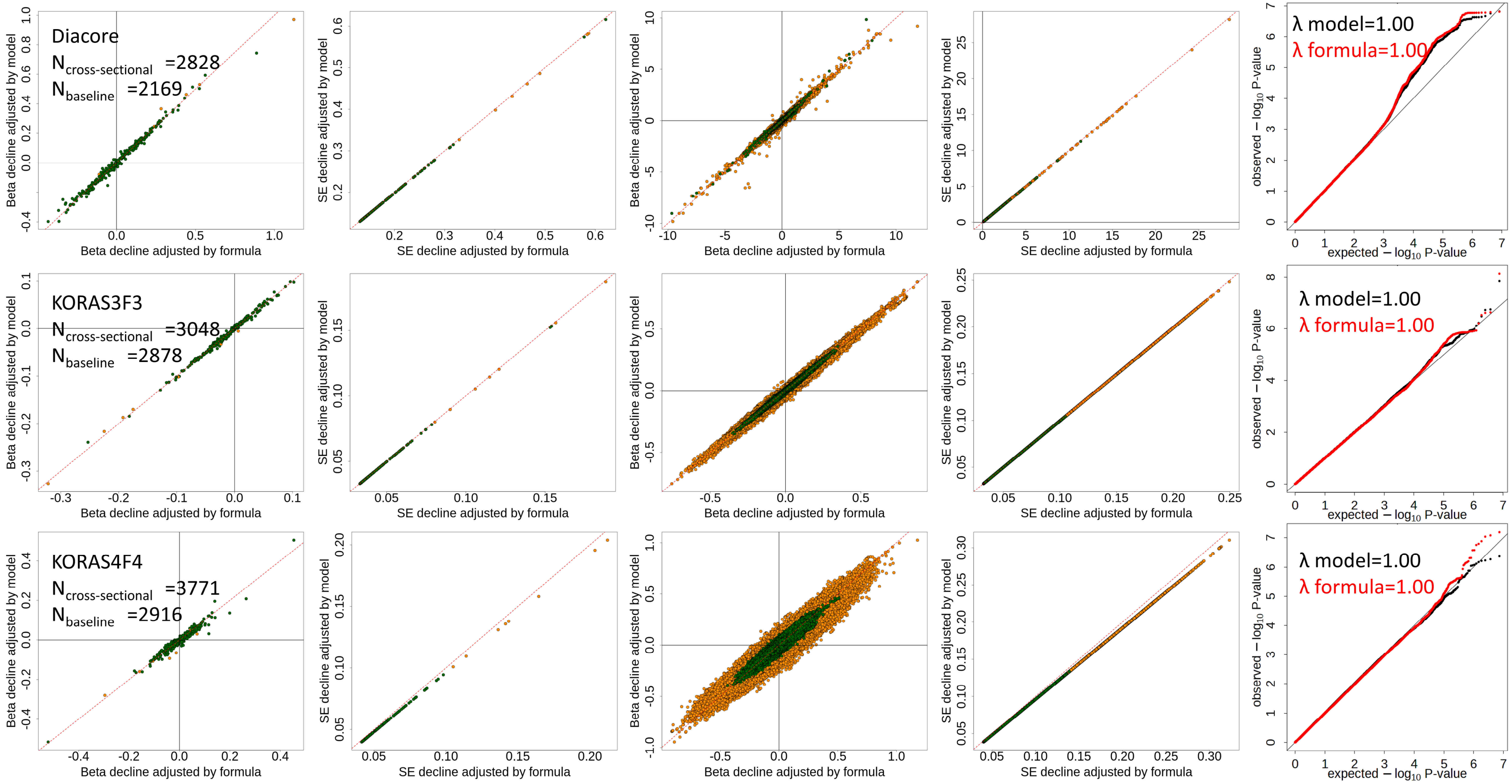

Supplementary Figure S4D: continued

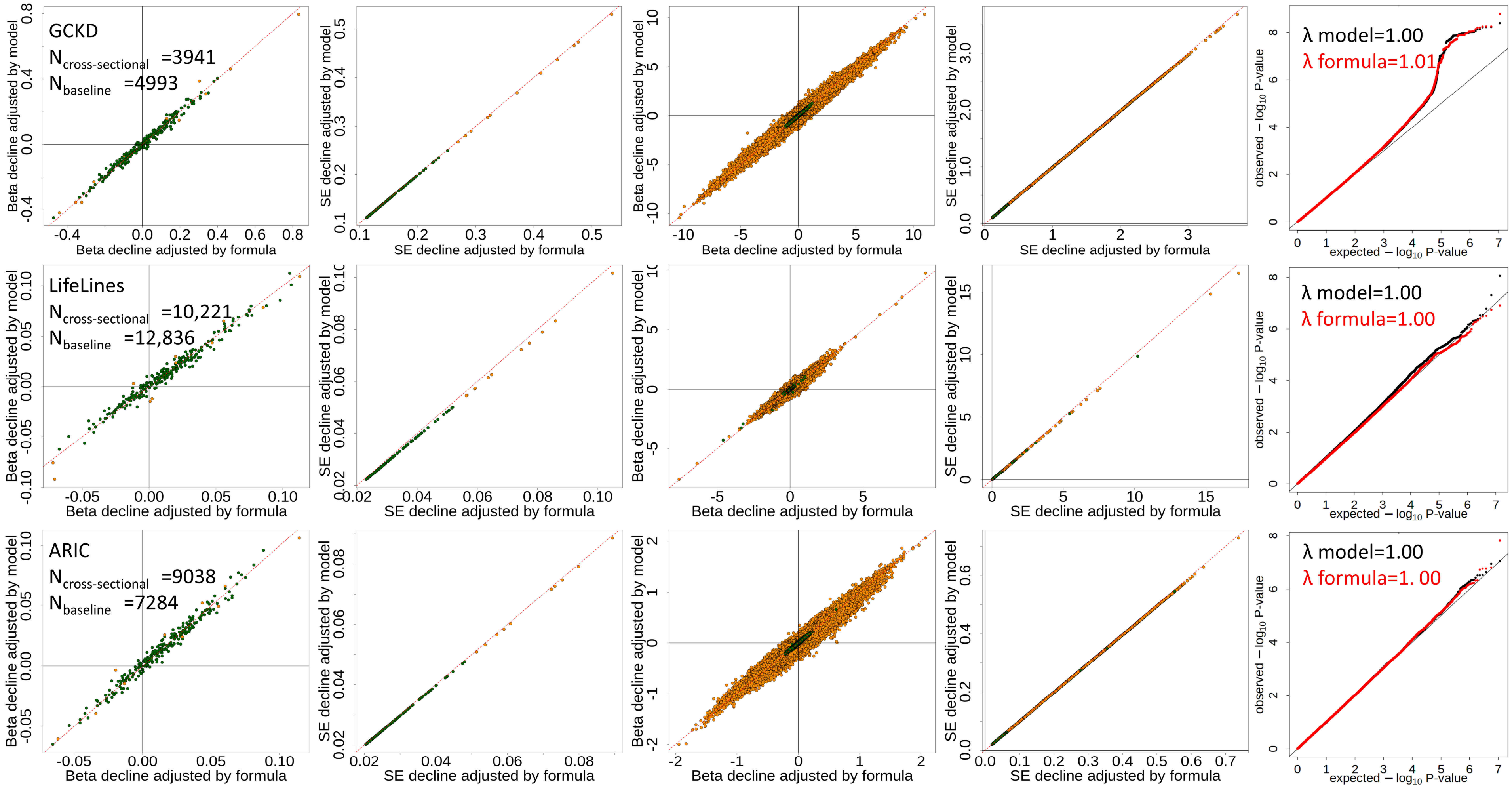

Supplementary Figure S5A: Region plots of the 4 variants in 3 loci identified for eGFR-decline unadjusted for eGFR-baseline. Shown are regional association plots (1st column) for cross-sectional eGFR[S12] ("eGFRcrea", n up to 765,348), (2nd and 3rd column) for eGFR-decline unadjusted for eGFR-baseline ("decline"; n up to 343,339; blue dashed line $P=0.05/263=1.90\times10^{-4}$ in 2nd column and P=0.05 in 3rd column), and (4th column) for eGFR-decline adjusted for eGFR-baseline ("$decline_{adj}$"; n up to 320,737). Reference variants are the cross-sectional eGFR lead variant (1st and 2nd column) and the $decline_{adj}$ lead variant (i.e. variant with the smallest P-value for $decline_{adj}$; 3rd and 4th column). Red lines indicate $P=5.00\times10^{-8}$. The decline signals coincide with the cross-sectional eGFR signals; decline lead variants are the same or highly correlated with cross-sectional eGFR lead variants (r^2=same, same, 1.00 and 0.93 for *UMOD-PDILT* (2), *PRKAG2* and *SPATA7*, respectively).

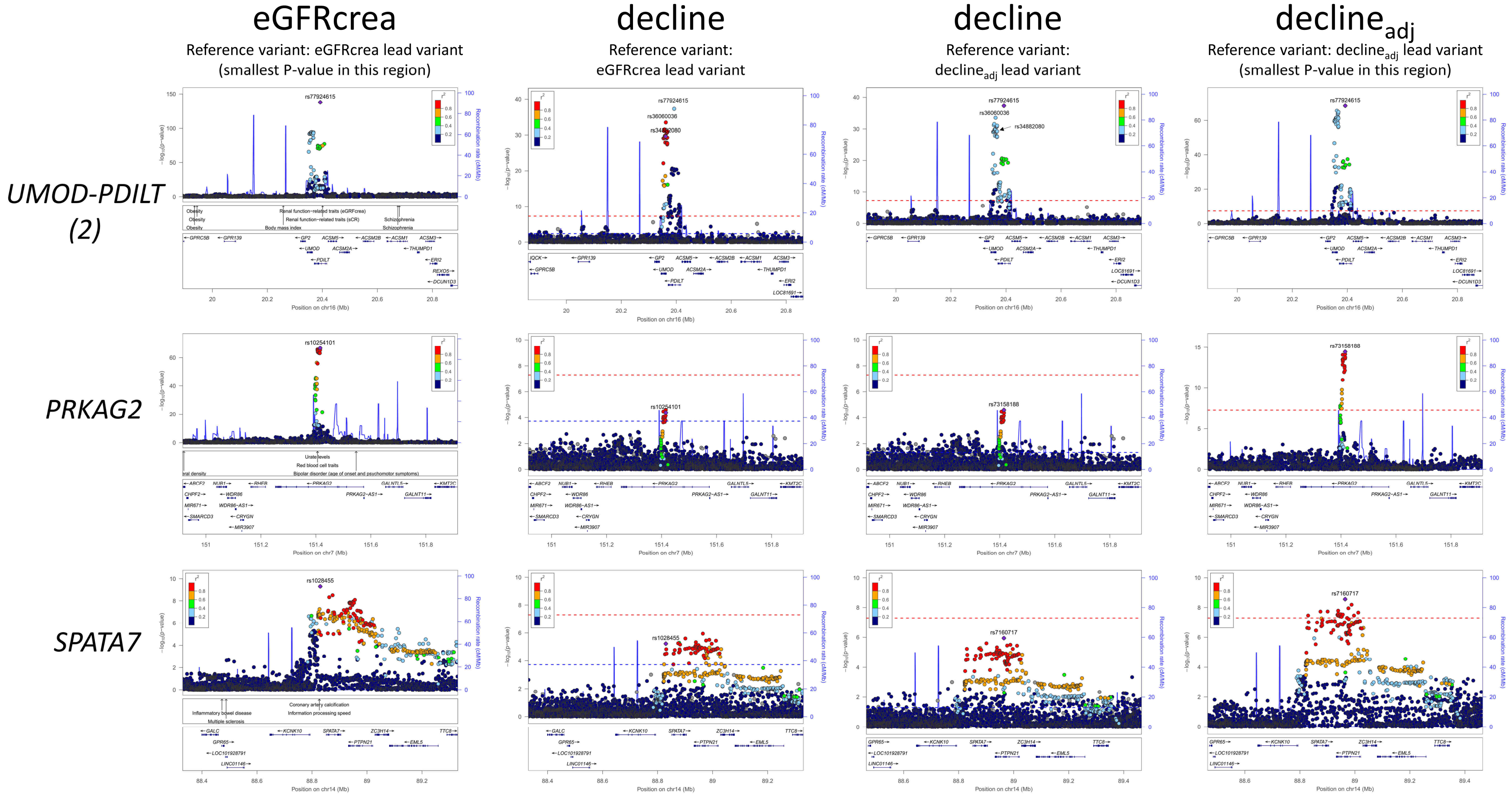

Supplementary Figure S5B: Regions of the 5 variants in 5 loci identified from GWAS for eGFR-decline adjusted for eGFR-baseline with significant association for eGFR-decline unadjusted for eGFR-baseline. Shown are regional association plots (1st column) for cross-sectional eGFR[S12] ("eGFRcrea", n up to 765,348), (2nd and 3rd column) for eGFR-decline unadjusted for eGFR-baseline ("decline"; n up to 343,339; blue dashed line $P=0.05/263=1.90\times10^{-4}$ in 2nd column and P=0.05 in 3rd column), and (4th column) for eGFR-decline adjusted for eGFR-baseline ("decline_{adj}"; n up to 320,737). Highlighted are lead variants for cross-sectional eGFR[S12] (1st and 2nd column; for *C15ORF54*, using the 2nd signal lead variant) or the decline_{adj} lead variant (3rd and 4th column). Red lines indicate $P=5.00\times10^{-8}$. Signals for decline_{adj} coincide with signals for cross-sectional eGFR.

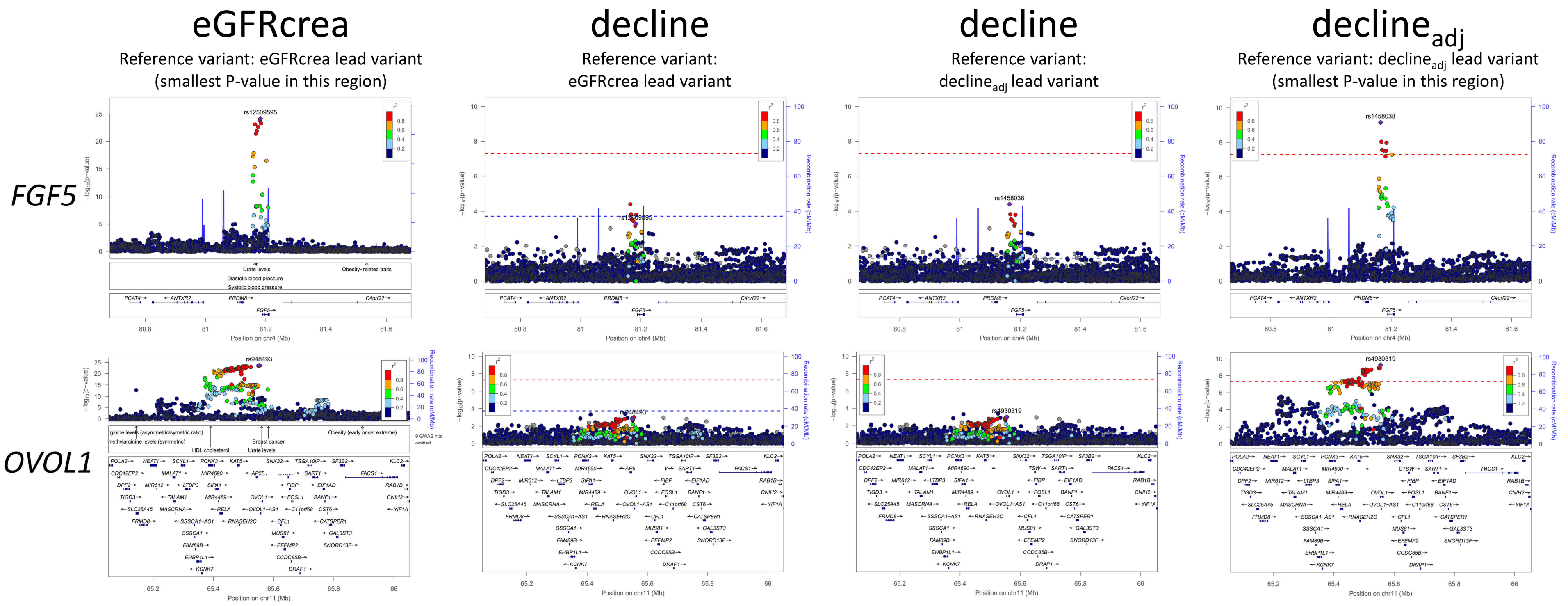

Supplementary Figure S5B (continued)

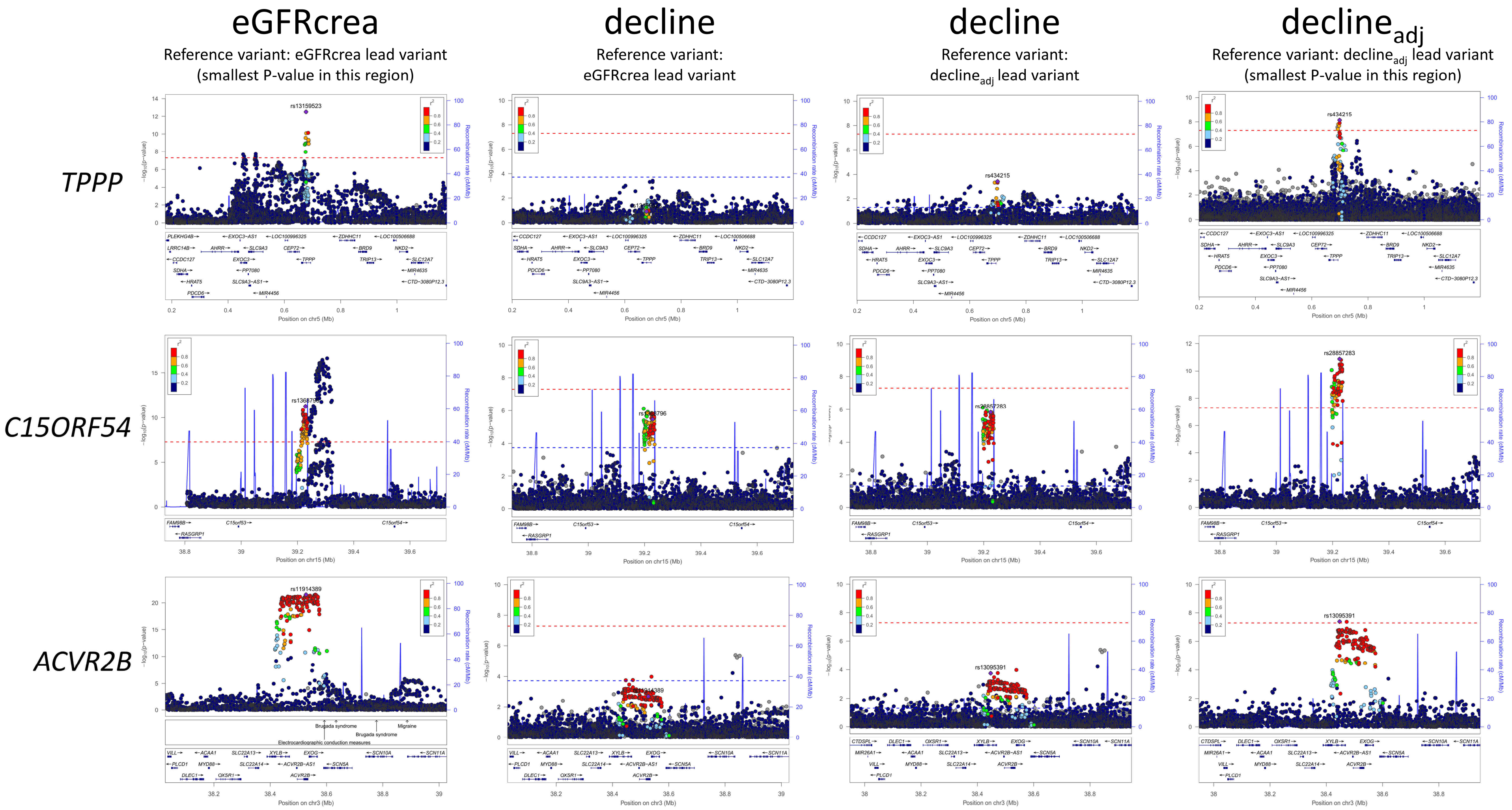

Supplementary Figure S5C: Regions of the 3 variants in 3 loci identified from GWAS for eGFR-decline adjusted for eGFR-baseline without significant association for eGFR-decline unadjusted for eGFR-baseline. Shown are regional association plots (1st column) for cross-sectional eGFR[S12] ("eGFRcrea", n up to 765,348), (2nd and 3rd column) for eGFR-decline unadjusted for eGFR-baseline ("decline"; n up to 343,339; blue dashed line $P=0.05/263=1.90\times10^{-4}$ in 2nd column and $P=0.05$ in 3rd column), and (4th column) for eGFR-decline adjusted for eGFR-baseline ("decline$_{adj}$"; n up to 320,737). Highlighted are lead variants for cross-sectional eGFR[S12] (1st and 2 column) and decline$_{adj}$ lead variants (3rd and 4th column). Red lines indicate $P=5.00\times10^{-8}$. Signals for decline$_{adj}$ coincide with signals for cross-sectional eGFR; there is no association for decline (unadjusted) in these regions.

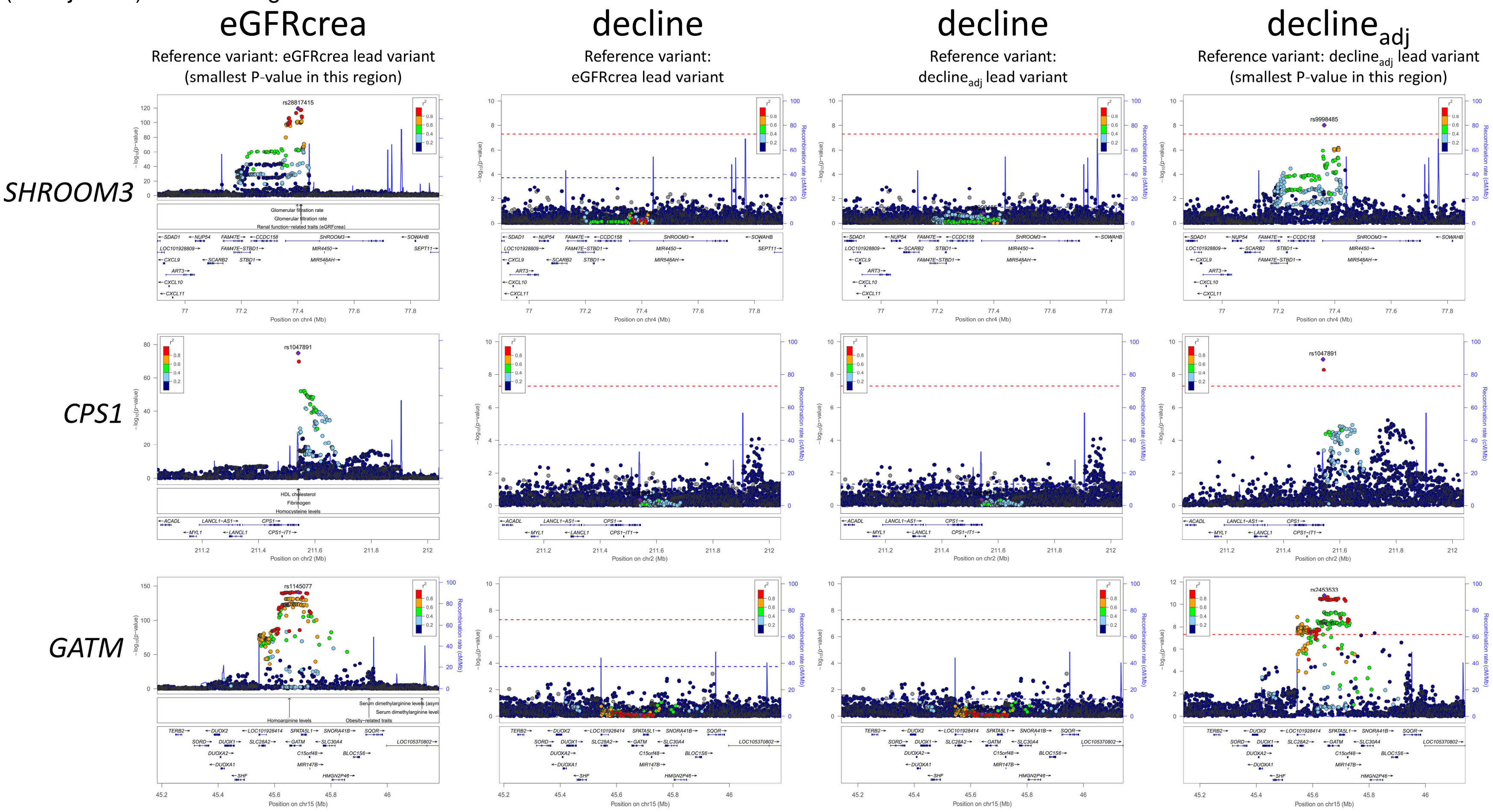

Supplementary Figure S6: Age-dependency of cross-sectional eGFR and age-dependency of SNP-effects on cross-sectional eGFR in UK Biobank. We conducted SNP-by-age interaction analyses on cross-sectional eGFRcrea and eGFRcys in individuals from UK Biobank that were independent from the GWAS (n=351,462; i.e. excluding the 15,442 individuals in the eGFR-decline GWAS) using linear regression with covariates sex, age, SNP, SNP-by-age and outcome eGFRcrea or eGFRcys. The SNP-effect was modelled as linear dosage effect (for main effect and in interaction term; i.e. additive genetic effect per allele). Age was centered at 50 years and modelled linearly as well as allowing for a smooth non-linear change by age. For cross-sectional eGFRcrea (1st row) and eGFRcys (2nd row), we show the age-dependency **(Z)** of the main age effect on eGFRcrea and eGFRcys, **(A)** on the SNP-effects of the 4 variants identified for eGFR-decline (unadjusted for eGFR-baseline), **(B)** on the SNP-effects of the 5 variants identified for eGFR-decline adjusted for eGFR-baseline with significant association for eGFR-decline unadjusted for eGFR-baseline, and **(C)** on the SNP-effects of the 3 variants identified for eGFR-decline adjusted for eGFR-baseline without significant association for eGFR-decline unadjusted for eGFR-baseline. In **A-C**, the main age effect was modelled non-linearly (to avoid residual confounding) and the interaction effects modelling the age-dependency of the SNP-effect linearly (green lines) are the ones reported in **Table 3**.

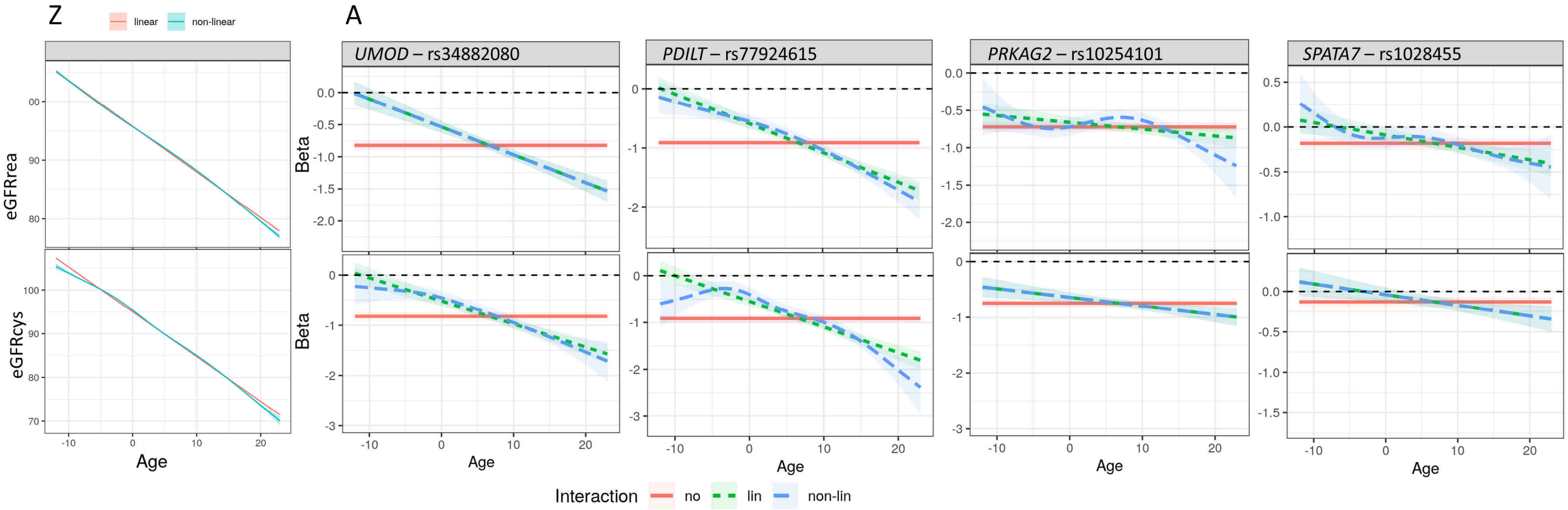

Supplementary Figure S6 (continued)

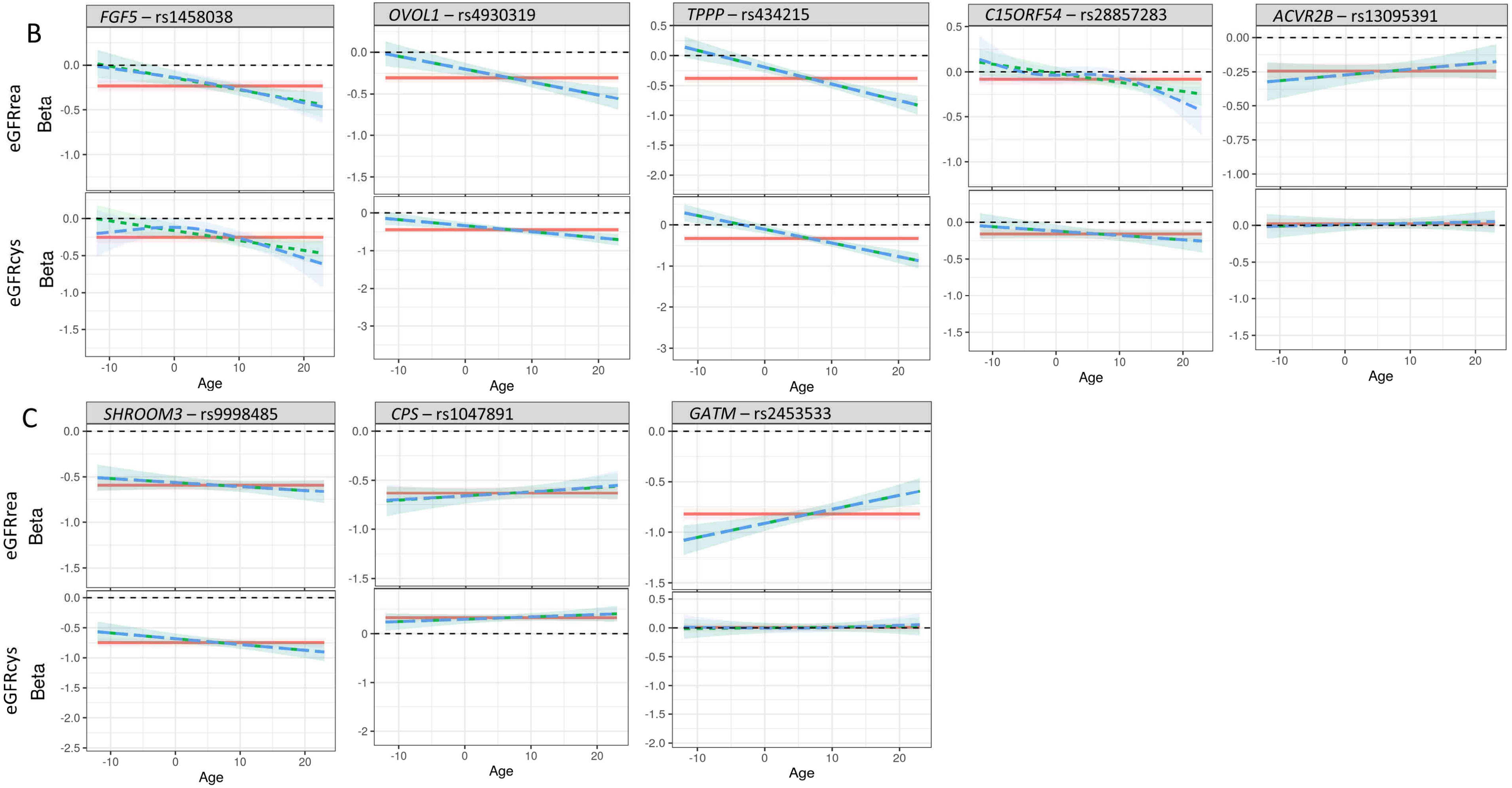

Supplementary Table S1: Description of participating studies: study design						
**Study**	**Full name of the study**	**Subgroup**	**Ancestry (EA/AA/HIS/EAS/SA)**	**Study Design (if not population-based, please specify selection and/or enrichment strategy)**	**Important study references, e.g. design paper (PMID)**	**Serum creatinine assay and year of measurement, baseline**
ADVANCE	Action in Diabetes and Vascular disease: preterAx and diamicroN mr Controlled Evaluation	5	EA	factorial, multicentre, randomised controlled trial, with a 5- to 6-year follow-up.	11848259	Jaffe, 2001-2003
		6	EA			
		UKB	EA			
AFTER EU	AFTER (EURAGEDIC) European Rational Approach for the Genetics of Diabetic Complications		EA	Adult onset Type 1 Diabetes	18496510, 20357380	Modified Jaffe
Amish	Amish Studies		EA	Population based "founder" cohort	18440328, 26374108, 15621217	Modified kinetic Jaffe reaction
ARIC	Atherosclerosis Risk in Communities study	AA	AA	Population-based	2646917	Modified kinetic Jaffé reaction, 1989
		EA	EA			
ASPS	Austrian Stroke Prevention Study		EA	Population-based	10408549, 7800110	Modified kinetic Jaffe reaction, 1991 - 2005
ASPS-Fam	Autrian Stroke Prevention Family Study		EA	Family-based	25309438, 25443291	Modified kinetic Jaffe reaction, 2006 - 2012
BioMe	BioMe™ BioBank Program	Omni AA	AA	Population-based	25349204	Jaffe, 2008
		Omni EA	EA			
		Omni HA	HIS			
CHS	Cardiovascular Health Study	AA	AA	Population-based	1669507	Colorimetric method on a Kodak Ektachem 700 Analyzer (Eastman Kodak, Rochester, NY), 1989-90 and 1992-93
		EA	EA			
Cilento	Cilento Study		EA	Population-based, Isolated Population Study	17476112, 19550436	Jaffe, 2005
DECODE	deCODE genetics/Amgen		EA	Population-based	20686651, 25082825	Ensymatic and modified kinetic Jaffe reaction assay since 1997
DIACORE	DIAbetes COhoRtE		EA	Prospective cohort study of patients with diabetes mellitus type 2	23409726	Serum Creatinine was measured 2010-2013 using an enzymatic assay traceable to NIST.
ESTHER	Epidemiological investigation of the chances of preventing, recognizing early and optimally treating chronic diseases in an elderly population		EA	Population-based	23446902, 15578318	Kinetic Jaffe-method, 2000 - 2002
FHS	The Framingham Heart Study		EA	Community- and family-based	5921755, 1208363, 17372189	Modified Jaffe method
FINCAVAS	The Finnish Cardiovascular Study		EA	Fincavas follow-up cohort of consecutive patients undergoing exercise stress test	16515696	Entzymatic photometric, 1992-2015
GCKD	German Chronic Kidney Disease study		EA	Included are European ancestry CKD patients aged 18-74 years with an eGFR between 30–60 mL/min per 1.73 m2 or an eGFR >60 mL/ min per 1.73 m2 and a urinary albumin-to-creatinine ratio (UACR) >300 mg/g, albuminuria >300 mg/day, a urinary protein-to-creatinine ratio >500 mg/g, or proteinuria >500 mg/day	21862458, 25271006	Serum creatinine was measured using the Ceratinine plus enzymatic assay (Roche) on a Modular (P) analyzer in 2012
Geisinger Research (MyCode)	MyCode Community Health Initiative		EA	Population-based	26866580	Enzymatic method done by Roche Cobas instruments, 1996+
HANDLS	Healthy Aging in Neighborhoods of Diversity across the Life Span study		AA	Population-based prospective longitudinal study	20828101	Modified Jaffe 2004-2009
HYPERGENES	Hypergenes - European Network for Genetic-Epidemiological Studies	controls	EA	Case-control for Hypertension	22184326	Jaffe assay 2002
Jackson Heart Study (JHS)	Jackson Heart Study		AA	Community and family-based	16320381	IDMS calibrated serum creatinine was used from visit 1 and visit 3… creatinine measurements were made from 2000 on but calibration to the same standard was done in 2015 (see PMID: 25806862 for a full description).
JMICC	Japan Multi-institutional Collaborative Cohort (J-MICC) Study		EAS	Population-based	17696755, 32963210	Enzymatic method, 2007-2010
KORA	Cooperative Health Research in the Augsburg Region	F3	EA	Population-based	16032514	Modified kinetic Jaffe reaction, 1994
		F4	EA			
Lifelines	Lifelines Cohort Study		EA	Population-based	18075776, 25502107, 26333164	Enzymatic, IDMS traceable, Roche (Modular); 2006-2013
MDC-CC	Malmö Diet and Cancer Study-Cardiovascular Cohort		EA	Population-based	11916347	Jaffé method and the IDMS-traceable standard was used
MESA	Multi-Ethnic Study of Atherosclerosis	AFR	AA	Population-based without CVD	12397006	Baseline is year 2002, exam 2 2004, exam 3 2005 and exam 4 2007. All assays rate relectance spectrophotometry using thin film adaptation of the creatine aminohydrolase method on the Vitros analyzer (Johnson and Johnson Clinical Diagnositcs)
		EAS	EAS			
		EUR	EA			
		HIS	HIS			
METSIM	Metabolic Syndrome in Men study		EA	Population-based	28119442	Kinetic Jaffé method, 2005-2010
NESDA	Netherlands Study of Depression and Anxiety		EA	Population-based, predominantly cases with major depression	18763692	Partly Jaffe, partly enzymatic; 2004-2007
OGP	Ogliastra Genetic Park Study		EA	Population-based	20823129	Colorimetric method Jaffè without deproteinization (Biotecnica instruments).Creatinine forms a colored orange-red complex in an alkaline picrate solution. The difference in absorbance at fixed times during conversion is proportional to the concentration of creatinine in the sample. 2005-2008
PIVUS	Prospective Investigation of Vasculature in Uppsala Seniors		EA	Population-based	16141402	Kinetic jaffe method
POPGEN	POPGEN control sample		EA	Population-based	16490960	Serum creatinine was measured 2005-2008 using an enzymatic assay
PREVEND	Prevention of Renal and Vascular End-stage Disease study		EA	Population-based	12356629	An isotope dilution mass spectrometry (IDMS) traceable enzymatic method on a Roche Modular analyzer using reagents and calibra- tors from Roche (Roche Diagnostics, Mannheim, Germany) '97-'98
RS	Rotterdam Study	I	EA	Population-based	29064009	Enzymatic assay, 1999
		II	EA			Enzymatic assay, 2000
		III	EA			Enzymatic assay, 2006
SHIP	Study of Health in Pomerania	1	EA	Population-based	20167617	Jaffe, 2002
SiMES	Singapore Malay Eye Study		EAS	Population-based	17365815, 21490949	Jaffe, 2004-2007
SINDI	Singapore Indian Eye Study		EAS	Population-based	19995197, 24244560	Jaffe, 2007-2009
SOLID-TIMI 52	SOLID-TIMI 52	EA	EA	Clinical trial	21982651	Jaffe, 2010
		EAS	EAS			
		SA	SA			

STABILITY	STabilization of Atherosclerotic plaque By Initiation of darapLadlb TherapY	EA	EA	Clinical trial	24678955, 20934559	Jaffe, 2009
		EAS	EAS			
		SA	SA			
ULSAM	Uppsala study of adult men		EA	Population-based	21335440	Kinetic jaffe method
Vanderbilt	Vanderbilt BioVU	660	EA	Population-based with enrichment for a variety of disease studies	18500243	Extracted from clinical records
		AA1M	AA			
		Omni1	EA			
		Omni5	EA			
YFS	The Young Finns Study		EA	Population-based	18263651, 23069987	Serum creatinine was determined spectrophotometrically by the Jaffé method (picric acid; Olympus Diagnostica GmbH) from frozen plasma samples. Year 2001.
AugUR	The German AugUR study		EA	Prospective cohort study in the elderly	26489512	Serum Creatinine was determined on a encymatic Siemens-Kit ECREA, 2018
HUNT	Trøndelag Health Study, Norway		EA	Population-based	22879362	Modified kinetic Jaffé reaction, 1995-1997
MGI	Michigan Genomics Initiative		EA	Hospital-based		Jaffe, variable year of measurement
UKBB	Uk Biobank		EA	Population-based	25826379	Enzymatic analysis on a beckman Coulter AU5800

AA: African American ancestry; EA: European ancestry; HIS: Hispanics; SA: South Asian ancestry; EAS: East Asian ancestry

Supplementary Table S2: Description of participating studies: genotyping and imputation												
Study	Exclusions prior to genotyping and/or ge	Genotyping Array	Genotype calling	QC filters for genotyped SNPs used for imputation	No of SNPs used for imputation	Pre-phasing software [1]	Imputation	Imputation reference panel	Filtering of imputed genotypes	Software used for GWAS[3]	Handling of population stratification	Type of reported imputation quality
ADVANCE	Ethnic outliers, sex mismatches, call rate < 95%	Affymetrix 5.0, Affymetrix 6.0, Affymetrix UKB	Affymetrix power tools 1.17.0	avg_het <23% or >30%; call rate <97%; MAF <1%; snp call rate <95%; HWE <0.001;	Affymetrix 5.0 : 363,062; Affymetrix 6.0 : 702,628; Affymetrix UKB : 759,238	ShapeIT2	Impute2	1000 Genomes Project Phase 3 Version 5	MAF<0.005; info score<0.3	PLINK 1.90 beta	PC1-PC2	Info Score
AFTER EU	sample call rate <98%, extreme heterozygosity, sex mismatches, non-European ancestry, cryptic relatedness, duplicates	Illumina HumanCore Exome v1.0/v1.1	Illumina Genome Studio	Call Rate <=95%, HWE Filter 10e-06, INDELS removed, non 1KG variants removed, 40% MAF difference with 1000G, Duplicate SNPs	318,207	ShapeIT2	Minimac3	1000 Genomes Project Phase 3 Version 5 (updated on Oct 20, 2015)	none	EPACTS	PC1-PC5	r^2
Amish	age <18, severe chronic disease, call rate <95%, pHWE<10E-6	Affymetrix 500K and 6.0	BRLMM	Sample call rate <95%, pHWE<5E-6, MAF <0.01	397,704	ShapeIT2	Impute2	1000 Genomes Project Phase 1 Release Version 3 ALL (March 2012)	none	MMAP	NA	Info Score
ARIC EA	Of the 9713 genotyped individuals of European ancestry, we excluded 658 individuals based on discrepancies with previous genotypes, disagreement between reported and genotypic sex, one randomly selected member of a pair of first-degree relatives, or outlier based on measures of average DST or >8 SD away on any of the first 10 principal components.	Affymetrix 6.0	Birdseed	call rate <95%, MAF<0.5%, pHWE<10e-5	682,749	ShapeIT2	Impute2	1000 Genomes Project Phase 1 Release Version 3 ALL (March 2012)	none	SNPTEST v2	PC1-PC10	Info Score
ARIC AA	Of the 3,207 genotyped individuals of Africa ancestry, we excluded 336 individuals based on discrepancies with previous genotypes, disagreement between reported and genotypic sex, one randomly selected member of a pair of first-degree relatives, or outlier based on measures of average DST or >6 SD away on any of the first 10 principal components.	Affymetrix 6.0	Birdseed	call rate <95%, MAF<1%, pHWE<10e-5	773,317	ShapeIT2	Impute2	1000 Genomes Project Phase 1 Release Version 3 ALL (March 2012)	none	SNPTEST v2	PC1-PC10	Info Score
ASPS	Ethnic outliers; duplicates; gender mismatch; cryptic relatedness; sample call rate < 98%; excess heterozygosity	Illumina Human610-Quad BeadChip	Illumina	call rate < 98 %; MAF < 1% ; pHWE < 5×10-6	566,930	ShapeIT2	Impute2	1000 Genomes Project Phase 1 Release Version 3 ALL (March 2012)	none	EPACTS (v3.2.6)	PC1-PC4	Info Score
ASPS-Fam	Ethnic outliers; duplicates; gender mismatch; cryptic relatedness; sample call rate < 98%; excess heterozygosity	Affymetrix Genome-Wide Human SNP Array 6.0	Birdseed v2	call rate < 98 %;MAF < 5%;pHWE < 1×10-6	501,288	ShapeIT2	Impute2	1000 Genomes Project Phase 1 Release Version 3 ALL (March 2012)	none	EPACTS (v3.2.6)	PC1-PC4	Info Score
BioMe	none	Illumina HumanOmniExpressExome-8 v1.0	BeadStudio	Removed samples:<br>1. Sample call rate: < 98%<br>2. Heterozygosity: coefficient < -0.1 or > 0.3 for common variants (MAF>1%)<br>3. inbreeding coefficient < 0.4 or > 0.9 for rare variants (MAF<1%)<br>4. MAF = 0<br>5. HWE < 1x10-5	AA/HIS: 828,109 EA: 688,734	AA/HIS: ShapeIT2 EA: minimac	AA/HIS: IMPUTE2 EA: Michigan Imputation Server	AA/HIS: 1000 Genomes Project Phase 1 Release Version 3 EA: Haplotype Reference Consortium 1.1	none	EPACTS-3.2.6-patched	PC1-PC8	AA/HIS: Info Score EA: r^2
CHS AA	Beyond laboratory genotyping failures, participants were excluded if they had a call rate<=95% or if their genotype was discordant with known sex or prior genotyping (to identify possible sample swaps).	Illumina HumanOmni1-Quad_v1 BeadChip	Illumina GenomeStudio	call rate < 97%, HWE P < 10-5, > 1 duplicate error or Mendelian inconsistency (for reference CEPH trios), heterozygote frequency = 0	940,567	no pre-phasing	Impute2	1000 Genomes Project Phase 3	Variants with insufficient effective minor alleles are filtered prior to analysis. This threshold was set at 5 effective alleles. Where effective alleles is defined as MAF*sampleN*2*impQuality.	custom R software	PC1-PC5	r^2
CHS EA	European ancestry participants were excluded from the GWAS study sample due to the presence at study baseline of coronary heart disease, congestive heart failure, peripheral vascular disease, valvular heart disease, stroke or transient ischemic attack or lack of available DNA. Beyond laboratory genotyping failures, participants were excluded if they had a call rate<=95% or if their genotype was discordant with known sex or prior genotyping (to identify possible sample swaps).	Illumina 370CNV BeadChip	Illumina BeadStudio	call rate < 97%, HWE P < 10-5, > 2 duplicate errors or Mendelian inconsistencies (for reference CEPH trios), heterozygote frequency = 0, SNP not found in HapMap.	359,592	MaCH	Minimac1	1000 Genomes Project Phase 3	Variants with insufficient effective minor alleles are filtered prior to analysis. This threshold was set at 10 effective alleles. Where effective alleles is defined as MAF*sampleN*2*impQuality.	custom R software	PC1-PC5	r2
Cilento	Gender mismatch	Illumina 370K (n=859) Illumina OmniExpress(n=758)	Illumina BeadStudio	SNPs in common between the two arrays, call rate<95%, MAF<1%.	~190,000	Eagle	Sanger Imputation Service	Haplotype Reference Consortium	none	EPACTS (fixed version febbrary 2017)	NA	Info Score
DECODE	Call rate < 97%	The chip-typed samples were assayed with the Illumina HumanHap 300, HumanCNV 370, HumanHap 610, HumanHap 1M, HumanHap 660, Omni-1, Omni 2.5 or Omni Express bead chips at deCODE genetics	Graphtyper	Yield < 95%, MAF>0.01, HW < 0.001		Inhouse software	Inhouse software, similar to IMPUTE	Icelandic reference panel - variants matched with Haplotype Reference Consortium or 1000 Genomes Project Phase 3	None	Inhouse software	for quantitative traits: BOLT LMM or variance covariance matrix prop. to the kinship matrix / for binary: adj. for county of birth	Info Score
DIACORE	all patients included	Axiom UK Biobank Array	Axiom GT1 in Genotyping Console 4.0	1) Missing phenotype<br>2) Ancestry not European<br>3) Relatedness 2nd degree or closer<br>4) Genetic gender discordant with phenotypic gender<br>5) Gonosomal aberation<br>6) Excess of Heterocygosity<br>7) Low callrate	799,756	ShapeIT2	Minimac1	1000 Genomes Project Phase 3 Version 5	none	epacts 3.2.6	PC1-PC10	r^2

ESTHER	Quality control was performed according to Nat. Protoc. 2010 Sept.; 5(9): 1564-1573, Anderson et al.: Gender mismatch, sample call rate < 97%,removal of duplicated or related samples, removal of ethnic outliers (Germans only remained), MAF 0.01, GENO 0.05, HWE 0.00001	Illumina Infinium OncoArray-500K BeadChip	GenomeStudio	MAF < 0.01	368,205	ShapeIT	Impute2	1000 Genomes Project Phase 3 Version 5	none	SNPTEST v2.5.2	not required	Info Score
FHS	call rate >97%,sample failures, genotyped sex different from recorded sex, extreme heterozygosity or high Mendelian error rate	Affymetrix GeneChip Human Mapping 500K Array Set® and 50K Human Gene Focused Panel®	Affymetrix BRLMM	call rate ≥97%, pHWE≥1E-6, Mishap p≥1e-9, ≤100 Mendel errors, MAF≥1%	412,053	ShapeIT	MACH	1000 Genomes Project Phase 1 Release Version 3 (March 2012)	none	GWAF	PCs asscoated with trait with p<0.05	r^2
FINCAVAS	call rates < 95%, pHWE < 1E-6, sex mismatch, MDS outliers, excess heterozygosity	Illumina HumanCore Exome and Metabochip	Genome Studio	call rate<95%, pHWE<1e-6, monomorphic removed	HCE: 306,474. MC: 155,499	Eagle2	Minimac3	Haplotype Reference Consortium 1.1	None	EPACTS	PC1-PC5	r^2
GCKD	Call rate < 97%, failed sex check, outside 2 SD of mean heterozygosity, cryptic relatedness and genetic ancestry outlier	Illumina Omni2.5Exome BeadChip	Illumina GenomeStudio	Exclude SNPs with call rate < 96%, or HWE p < 1E-5, or MAF < 1%	2,337,794	Eagle	Minimac3	Haplotype Reference Consortium 1.1	none	EPACTS	no associated PCs	r^2
Geisinger Research (MyCode)	none	Illumina Human Omni express Exome	Illumina's Genotype studio	Removed samples and markers having:<br>1. IMPUTE2 info score < 0.7<br>2. Marker call rate < 99%<br>3. Sample call rate < 90%<br>4. MAF < 0.01<br>5. HWE < 1e-07<br>6. Removed SNPs having insertions and deletions	589,485	SHAPEIT 2	Impute2	1000 Genomes Project Phase 1 Release Version 3 ALL (March 2012)	Removed SNPs with info score<0.7	PLATO v0.0.1	not required	Info Score
HANDLS	Ethnic outliers, cryptic relateds, and sex mismatches, call rate < 95%	Illumina 1M genotyping array	Illumina GenomeStudio	MAF < 0.01, HWE pvalue < 1.0E-07, call rate < 95%	907,763	MACH 1.0	Michigan Imputation Server	1000 Genomes Project Phase 3 Version 5	None	EPACTS (v3.2.6)	PCs	r^2
HYPERGENES	Ethnic outliers, sex mismatches, related, call rate<95%; Extremes in heterozygosity	Illumina 1M Duo genotyping array	Illumina GenomeStudio	MAF<0.01; Call rate <99%; HWE < 0.00000004	909,532	ShapeIT	Minimac1	1000 Genomes Project Phase 1 Release Version 3 (March 2012)	none	EPACTS (v3.2.6)	PCs	r^2
Jackson Heart Study (JHS)	sex mismatches, sample duplications or swaps, sample call rate <95%	Affymetrix 6.0	Birdseed	call rate <95%	868,969	MACH 1.0	Minimac1	1000 Genomes Project Phase 1 Release Version 3 (March 2012), ALL	none	EPACTS (v3.2.6)	PC1-PC10 and kinship matrix for continuous traits	r^2
JMICC	sample call rate < 98 %, sex mismatches, related samples (IBD 0.1875), samples not mapping to JPT (1000 genomes)	Illumina HumanOmniExpressExome	GenomeStudio	Call rate < 98%, pHWE <10e-6, MAF < 1 %, exclude SNPs do not match or not present in 1000 Genomes phase 3 reference panel, remove SNPs with allele freqeuncy difference >20% between scaffold and EAS in 1000GP3, remove duplicates	570,162	ShapeIT2	Minimac3	1000 Genomes Project Phase 3	none	EPACTS	PC1-PC5	r^2
KORA_F3	check for European ancestry, check for population outlier	Illumina Omni 2.5/Illumina Omni Express	Genome Studio	call rate >97%, missmatch of phenotypic and genetic gender, 5SD from mean heterozygosity rate, comparison with other genotyping of the same individuals (Metabochip, Exome, Omni)	587,981	ShapeIT	Michigan Imputation Server	1000 Genomes Project Phase 3 Version 5	none	EPACTS (v3.2.6)	PC1-PC10	r^2
KORA_F4	check for European ancestry, check for population outlier	Affymetrix Axiom	Affymetrix Software	call rate >97%, missmatch of phenotypic and genetic gender, 5SD from mean heterozygosity rate, comparison with other genotyping of the same individuals (Metabochip, Exome, Omni)	508,532	ShapeIT	Michigan Imputation Server	1000 Genomes Project Phase 3 Version 5	none	EPACTS (v3.2.6)	PC1-PC10	r^2
Lifelines	call rate <95%; sex mismatch; heterozygosity > 4SD from mean; non-CaucasiansIBS	Illumina Cyto SNP12 v2	GenomeStudio	samples with call rate < 0.8, excess heterozygosity, non-Caucasian ethnicity (as determined by PCA), high relatedness (pi-hat > 0.4) or a gender mismatch; SNPs with MAF < 1%, a HWE p-value ≤10-3, or a callrate < 95%	257,581		Minimac1	1000 Genomes Project Phase 1 Release Version 3 (March 2012)	none	PLINK 1.90 beta	PC1-PC10	r^2
MDC-CC	1. bad call rate<br>2. excess homozygosity<br>3. failed gender check<br>4. Related individuals/duplicates<br>5. Popoulation outliers	Illumina HumanOmniExpressExome BeadChip v. 1.0	GenomeStudio v2011.1	monormorphic, bad call rate (<95%), fail HWE (p<10^-6)	~800,000	ShapeIT2	Impute2	1000 Genomes Project Phase 1 Release Version 3 ALL (March 2012)	none	SNPTEST	PC1-PC10	Info Score
MESA-AFR	Sex discrepancy, duplicates, call rate <95%, pHW <1E-6, heterozygosity, and outliers	Affymetrix Genome-Wide Human SNP Array 6.0	Birdseed v2	call rate≥95%, MA>1%	897,979	ShapeIT2	Michigan Imputation Server	1000 Genomes Project Phase 3 Version 5 ALL	none	EPACTS (v3.2.6)	PC1-PC3	r^2
MESA-EUR	Sex discrepancy, duplicates, call rate <95%, pHW <1E-6, heterozygosity, and outliers	Affymetrix Genome-Wide Human SNP Array 6.0	Birdseed v2	call rate≥95%, MA>1%	897,979	ShapeIT2	Michigan Imputation Server	Haplotype Reference Consortium	none	EPACTS (v3.2.6)	PC1-PC3	r^2
MESA-HIS	Sex discrepancy, duplicates, call rate <95%, pHW <1E-6, heterozygosity, and outliers	Affymetrix Genome-Wide Human SNP Array 6.0	Birdseed v2	call rate≥95%, MA>1%	897,979	ShapeIT2	Michigan Imputation Server	1000 Genomes Project Phase 3 Version 5 ALL	none	EPACTS (v3.2.6)	PC1-PC3	r^2
MESA-EAS	Sex discrepancy, duplicates, call rate <95%, pHW <1E-6, heterozygosity, and outliers	Affymetrix Genome-Wide Human SNP Array 6.0	Birdseed v2	call rate≥95%, MA>1%	897,979	ShapeIT2	Michigan Imputation Server	1000 Genomes Project Phase 3 Version 5 ALL	none	EPACTS (v3.2.6)	PC1-PC3	r^2
METSIM	call rate, sex check, duplicate removal, PC outliers	Illumina HumanOmniExpress-12v1	GenomeStudio	call rate<95%, MAF<1%		ShapeIT2	Minimac3	Haplotype Reference Consortium 1.1	none	EPACTS	mixed-model	r^2
NESDA	Non-Caucasians, XO and XXY samples, and samples with a call rate <90%, high genome-wide homo- or heterozygosity, excess IBS	Perlegen-Affymetrix 5.0; Affymetrix 6.0 907K	Birdseed	call rate≤95%; MAF<0.01; pHWE<1E-5; ambiguous location or allele with reference; >20% allele frequency difference from reference; ambiguous SNPs with a MAF>35%	378,163	MACH	Minimac3	1000 Genomes Project Phase 1 Release Version 3 ALL (March 2012)	none	EPACTS	PC1-PC3	r^2
OGP	sex mismatches, sample duplications or swaps, sample call rate <95%	Affymetrix 500K Gene Chip	BRLMM	call rate < 95 %;MAF < 1%;pHWE < 1×10-6	347,517	BEAGLE	Michigan Imputation Server	Haplotype Reference Consortium	none	EPACTS (v3.2.6)	Genomic Kinship for quantitative traits; First 3 PCs for binary traits	r^2
PIVUS	Call rate <95%; sex mismatch; extreme heterozygosity; related individuals; ancestry outliers	Illumina OmniExpress and Metabochip	Genome Studio	call rate <95%, HWE p<10^-6, MAF<1%	738,583	ShapeIT2	Impute4	Haplotype Reference Consortium	info<0.4	SNPTEST	PC1-PC2	Info Score

POPGEN	sample call rate < 90 %, sex mismatches, duplicates Samples (IBD 0.185), samples with heterozygosity outside mean +-3SD, samples not mapping to CEU (Hapmap), i.e. outside median +- 3*IQR and samples with batch problems, i.e. outside median +- 3*IQR	Affymetrix Axiom, Affymetrix 6.0, Illumina Immunochip (Beadchip), Illumina Metabochip, Illumina 550k (merged after QC)	Illumina GenomeStudio or Illumina Opticall	SNP call rate < 5%, HWE < 1x10^-5, no MAF for QC but MAF pre Imputation	1049248	ShapeIT2	Impute2	1000 Genomes Project Phase 1 Release Version 3 ALL (March 2012)	removed SNPs with info <= 0.3	EPACTS	not required	Info Score
PREVEND	call rate <95%; sex mismatch; non-Caucasians; duplicated samples	Illumina Cyto SNP12 v2	Illumina GenomeStudio	call rate < 95%; MAF <1%; pHWE< 1E-4	232571	ShapeIT2	Michigan Imputation Server	Haplotype Reference Consortium	none	SNPTEST V2	PC1-PC5 and exclusion of PC outliers	Info Score
RS-I	MAF < 0.05, SNP callrate < 0.95 and/or HWE p-value < 1 x 10-7, excess heterozygosity, gender swaps, genetic ancestry and familial relationships	Illumina 550K	GeneCall	MAF < 0.05, SNP callrate < 0.95 and/or HWE p-value < 1 x 10-7	502668	MaCH	Minimac 3	Haplotype Reference Consortium 1.0	none	RVTEST	PC1-PC5	r^2
RS-II	MAF < 0.05, SNP callrate < 0.95 and/or HWE p-value < 1 x 10-7, excess heterozygosity, gender swaps, genetic ancestry and familial relationships	Illumina 550K	GeneCall	MAF < 0.05, SNP callrate < 0.95 and/or HWE p-value < 1 x 10-8	490409	MaCH	Minimac 4	Haplotype Reference Consortium 1.0	none	RVTEST	PC1-PC5	r^2
RS-III	MAF < 0.05, SNP callrate < 0.95 and/or HWE p-value < 1 x 10-7, excess heterozygosity, gender swaps, genetic ancestry and familial relationships	Illumina 610K and 660K	GeneCall	MAF < 0.05, SNP callrate < 0.95 and/or HWE p-value < 1 x 10-9	517658	MaCH	Minimac 5	Haplotype Reference Consortium 1.0	none	RVTEST	PC1-PC5	r^2
SHIP	duplicate samples (by IBS), reported/genotyped gender mismatch, callrate <= 92%	Affymetrix SNP 6.0	Birdseed2	pHWE <= 0.0001 or CallRate <= 0.95 or monomorphic SNPs, duplicate IDs, inconsistent reference alleles, mapping problem to build 37	823635	Eagle2	Minimac3	Haplotype Reference Consortium 1.1	none	EPACTS-3.2.6-patched	not required	r^2
SiMES	monomorphic, call rate <95%, pHW <1E-6, heterozygosity, related individuals/duplicates, discordant ethnicity, and gender discrepancy.	Illumina Human610-Quad Beadchips	Genomestudio GenTrain and GenCall	T2D DIAMANTE protocol: exclude SNPs do not match or not present in 1000 Genomes phase 3 reference panel, remove SNPs with allele freqeuncy difference >20% between scaffold and reference population in 1000Gp3, remove duplicates	549947	ShapeIT	Michigan Imputation Server	1000 Genomes Project Phase 3 Version 5 ALL	none	EPACTS (v3.2.6)	PC1, PC2	r^2
SINDI	monomorphic, call rate <95%, pHW <1E-6, heterozygosity, related individuals/duplicates, discordant ethnicity, and gender discrepancy.	Illumina Human610-Quad Beadchips	Genomestudio GenTrain and GenCall	T2D DIAMANTE protocol: exclude SNPs do not match or not present in 1000 Genomes phase 3 reference panel, remove SNPs with allele freqeuncy difference >20% between scaffold and reference population in 1000Gp3, remove duplicates	552278	ShapeIT	Michigan Imputation Server	1000 Genomes Project Phase 3 Version 5 ALL	none	EPACTS (v3.2.6)	PC1-PC3	r^2
SOLID-TIMI 52	individuals excluded if call rate <97%, >3rd degree relative determined by kindship coefficient estimates from KING, GWAS gene didn't match annotated gender	Axiom® Biobank Plus Genotyping Array		call rates <95%, monomorphic, Hardy-Weinberg <E-6,	~547000		HAPI-UR	1000 Genomes Project Phase 1 Release Version 3 ALL (March 2012)	none	EPACTS (v3.2.6)	PC1-PC10	Info Score
STABILITY	individuals excluded if call rate <95%, >3rd degree relative determined by kindship coefficient estimates from KING, GWAS gene didn't match annotated gender	Illumina HumanOmniExpressExome-8 v1 array		call rates <95%, monomorphic, Hardy-Weinberg <E-7,	881788	ShapeIT2	Minimac3	1000 Genomes Project Phase 1 Release Version 3 ALL (March 2012)	none	EPACTS (v3.2.6)	PC1-PC10	r^2
ULSAM	Call rate <95%; sex mismatch; extreme heterozygosity; related individuals; ancestry outliers	Illumina 2.5M and Metabochip	Genome Studio	call rate <95%, HWE p<10^-6, MAF<1%	1621481	ShapeIT2	Impute4	Haplotype Reference Consortium	info<0.4	SNPTEST	PC1-PC2	Info Score
Vanderbilt-660	sex check, duplicate removal, call rate (<98%), HapMap concordance check	Illumina 660W	Genome Studio	call rate <98%, HWE<0.001, MAF <0.001	527715	ShapeIT	Minimac3	Haplotype Reference Consortium 1.1	none	EPACTS	PC1-PC3	r^2
Vanderbilt-AA1M	sex check, duplicate removal, call rate (<98%), HapMap concordance check	Illumina 1M	Genome Studio	call rate <98%, HWE<0.001, MAF <0.001	784048	ShapeIT	Minimac3	Haplotype Reference Consortium 1.1	none	EPACTS	PC1-PC3	r^2
Vanderbilt-Omni1	sex check, duplicate removal, call rate (<98%), HapMap concordance check	Illumina OMNI-Quad	Genome Studio	call rate <98%, HWE<0.001, MAF <0.001	924162	ShapeIT	Minimac3	Haplotype Reference Consortium 1.1	none	EPACTS	PC1-PC3	r^2
Vanderbilt-Omni5	sex check, duplicate removal, call rate (<98%), HapMap concordance check	HumanOmni5-Quad	Genome Studio	call rate <98%, HWE<0.001, MAF <0.001	3702007	ShapeIT	Minimac3	Haplotype Reference Consortium 1.1	none	EPACTS	PC1-PC3	r^2
YFS	call rates < 95%, pHWE < 1E-6, sex mismatch, MDS outliers, excess heterozygosity	Illumina 670k Custom	Illuminus	call rate<95%, pHWE<1e-6, monomorphic removed	542086	Eagle2	Minimac3	Haplotype Reference Consortium 1.1	None	EPACTS	PC1-PC5	r^2
AugUR	sex check, duplicate removal, relatedness, call rate (<98%), HapMap concordance check	Infinium® Global Screening Array-24 v1.0	GenomeStudio	call rate<95%, pHWE<1e-6, monomorphic removed, removed variants not in reference	614130	ShapeIT	Minimac3	1000 Genomes Project Phase 3 Version 5 ALL	None	rvtests	PC1-PC4	r^2
HUNT	Only Europeans were included for this analysis. Samples with call rate <99%, departures from HWE, duplicates, gender mismatch, unusual XY composition, mismatch with reference genome, and samples with contamination > 2.5% were removed	Illumina Hum	GenCall fro	call rate <95%, MAF<0.5%, pHWE<10e-5	368139	Eagle2	Minimac3	Haplotype Reference consortium release 1.1 + 2,201 low-coverage whole-genome sequences samples from the HUNT study	r^2≥0.3	SAIGE v0.3!	PC1-PC4	r^2
MGI	Only European individuals were used for analysis, duplicates, gender mismatch, unusual XY composition, related samples, and samples with contamination > 2.5% were removed	the Illumina Infinium CoreExome-24	GenomeStudio	Sample call rate < 99%, chromosomal call-rate drop > 5%	502255	Eagle	Minimac3	HRC	none	rvtests	PC1-PC4	r^2
UKBB	variants showing batch effects, plate effects, departures from HWE, sex effects, array effects, discordance across control replicates. Samples: ancestry outliers, outliers for heterozygosity and missingness. Further QC details can be found here : https://www.biorxiv.org/content/early/2017/07/20/166298	UK BiLEVE Axiom array, UK Biobank Axiom array	Axiom GT1 algorithm as implemented in the Affymetrix Power Tools software	Failed QC in > 1 batch, call rate < 95%, MAF < 0.0001, further details can be found here : https://www.biorxiv.org/content/early/2017/07/20/166298	670739	ShapeIT3	Impute4	Haplotype Reference Consortium	None	Quicktest	PC1-PC10	Info Score

[1] References for cited software: MACH (PMID: 19715440); ShapeIT (PMID: 22138821); Eagle (PMID: 27270109); Beagle (PMID: 21310274).

[2] References for cited software: ImputeV2 (PMID: 19543373); minimac3 (PMID: 27571263); PBWT (PMID: 24413527); Sanger Imputation server (PMID: 27548312); Michigan Imputation Server (PMID: 27571263).

[3] References for cited software: EPACTS (Kang, H.M. Epacts: Efficient and Parallelizable Association Container Toolbox. University of Michigan: Department of Biostatistics and Center for Statistical Genetics (2012); PMID: 20208533); SNPTest (PMID: 20517342); RegScan (PMID: 24008273); RVTESTS (PMID: 27153000); PLINK 1.90 (PMID: 25722852); GenABEL (PMID: 17384015); ProbABEL (PMID: 20233392); GWAF (PMID: 20040588); GEMMA (PMID: 22706312); mach2qtl (PMID: 21058334).

Supplementary Table S3: Description of participating studies: phenotype distribution

Study	Subgroup	Ancestry (EA/AA/HIS/EAS/SA)	Time to followup median [years]	Male %	Diabetes at baseline %	Age at baseline median	Age at baseline mean (SD)	eGFRcrea at baseline median (Q1, Q3)	n decline		
									overall	DM at baseline	CKD at baseline
ADVANCE	5	EA	4.35	70%	100%	67.2	67.4 (6.6)	72.1 (59.5, 86.3)	752	752	192
	6	EA	4.35	62%	100%	67.2	67.4 (6.6)	74.0 (62.8, 86.1)	2,169	2,169	436
	UKB	EA	4.35	59%	100%	68.4	67.4 (6.6)	69.3 (57.4, 83.2)	1,061	1,061	319
AFTER EU		EA	6.00	57%	100%	42.7	43.7 (11.1)	89.7 (67.0, 103.9)	831	831	140
Amish		EA	7.00	50%	1%	48.0	48.3 (16.3)	100.4 (88.9, 111.6)	798	NA	NA
ARIC	AA	AA	8.38	37%	20%	53.3	53.9 (5.8)	115.0 (102.8, 123.9)	1,903	298	NA
	EA	EA	8.69	47%	9%	54.6	54.8 (5.7)	101.1 (94.2, 107.4)	7,284	545	NA
ASPS		EA	1.00	43%	0%	65.0	65.8 (8)	73.6 (63.7, 88.1)	469	NA	NA
ASPS-Fam		EA	4.00	40%	0%	68.0	64.6 (10.6)	76.6 (65.3, 86.8)	104	NA	NA
BioMe	Omni EA	EA	2.77	35%	5%	62.9	63.8 (8.7)	76.3 (63.8, 89.1)	852	110	134
	Omni AA	AA	5.34	52%	3%	47.0	47.1 (13.7)	96.6 (79.9, 114.8)	1,717	NA	153
	Omni HA	HIS	4.97	37%	6%	48.4	48.7 (14.8)	92.5 (77.0, 106.1)	2,123	123	180
CHS	AA	AA	4.00	39%	24%	72.0	72.9 (5.7)	72.0 (59.5, 87.2)	481	NA	100
	EA	EA	6.00	44%	12%	71.0	72.3 (5.4)	65.2 (55.3, 75.9)	2,080	210	673
Cilento		EA	8.00	44%	10%	53.0	52.6 (19.7)	92.2 (80.2, 107.1)	788	NA	NA
DECODE		EA	14.00	47%	5%	44.0	45.4 (18.9)	94.1 (78.56, 108.9)	117,666	9,471	10,086
DIACORE		EA	2.96	60%	100%	66.7	65.5 (8.8)	82.4 (67.8, 92.9)	2,169	2,169	352
ESTHER		EA	5.00	42%	17%	62.0	61.6 (6.5)	93.0 (76.5, 103.0)	1,090	155	NA
FHS		EA	15.00	47%	6%	54.0	54.0 (14.9)	74.4 (47.1, 102.1)	2,925	195	1,296
FINCAVAS		EA	8.90	61%	13%	57.8	55.1 (13.2)	90.8 (78.4, 100.0)	835	123	NA
GCKD		EA	2.00	60%	35%	63.0	60.1 (12)	46.4 (37.1, 57.4)	3,941	1,341	3,115
Geisinger Research (MyCode)		EA	13.00	42%	13%	50.0	49 (15.2)	95.1 (80.1, 107.6)	36,286	4,659	2,237
HANDLS		AA	5.00	44%	18%	49.0	48.5 (9)	102.6 (87.6, 116.4)	735	135	NA
HYPERGENES	controls	EA	1.50	61%	0%	57.5	59.5 (9.8)	87.7 (76.9, 97.5)	461	NA	NA
Jackson Heart Study (JHS)		AA	6.60	38%	22%	55.5	55.1 (12.8)	96.5 (80.6, 110.0)	2,162	418	NA
JMICC		EAS	5.03	40%	3%	54.3	54.0 (9.4)	102.2 (96.0,108.4)	975	NA	NA
KORA	F3	EA	10.00	47%	2%	47.0	47.3 (13.0)	104.4 (94.0, 113.8)	2,878	NA	NA
	F4	EA	7.00	49%	3%	49.0	49.2 (13.9)	93.9 (81.9, 105.2)	2,916	NA	NA
Lifelines		EA	5.50	42%	3%	47.0	48.1 (11.4)	94.2 (83.1, 104.1)	10,553	322	142
MDC-CC		EA	16.49	41%	4%	56.3	56.4 (5.7)	80.7 (70.9, 90.6)	2,889		
MESA	AFR	AA	4.00	46%	17%	63.0	62.3 (10.1)	82.3 (70.1, 95.1)	1,283	198	122
	EAS	EAS	4.00	49%	13%	62.0	62.7 (10.2)	83.2 (71.3, 93.7)	615	NA	NA
	EUR	EA	4.00	48%	5%	63.0	62.4 (10.4)	75.4 (65.6, 86.2)	2,199	128	297
	HIS	HIS	4.00	48%	17%	61.0	61.4 (10.3)	84.2 (71.1, 94.3)	1,176	187	NA
METSIM		EA	4.00	100%	13%	57.0	57.7 (7.1)	93.5 (85.3, 100.0)	5,349	596	NA
NESDA		EA	6.00	34%	4%	43.0	41.9 (13.1)	103.7 (93.9, 114.8)	1,758	NA	NA
OGP		EA	6.34	33%	7%	51.7	53.2 (17.7)	73.1 (61.5, 85.0)	407	NA	NA
PIVUS		EA	5.13	50%	11%	70.1	70.2 (0.2)	81.7 (67.4, 90.6)	539	NA	NA
POPGEN		EA	6.00	53%	3%	57.0	54.7 (14.2)	91.0 (80.0, 100.9)	821	NA	NA
PREVEND		EA	4.00	52%	4%	49.0	49.6 (12.5)	84.3 (73.7, 94.4)	2,932	105	149
RS	I	EA	7.22	40%	13%	72.3	73.2 (7.6)	74.5 (64.3, 84.2)	1,338	121	116
	II	EA	9.30	46%	12%	62.0	64.8 (8.0)	81.7 (71.5, 91.1)	1,248	NA	NA
	III	EA	5.34	44%	9%	56.9	57.2 (6.9)	86.5 (76.9, 95.5)	2,289	NA	NA
SHIP	1	EA	3.00	48%	9%	55.0	54.5 (15.3)	90.4 (75.9, 103.8)	2,163	133	NA
SiMES		EAS	3.67	49%	31%	58.8	59.6 (11.0)	79.3 (64.7, 92.4)	1,451	405	191
SINDI		EAS	4.68	51%	40%	56.8	58 (10.0)	93.9 (80.5, 103.0)	1,554	552	NA
SOLID-TIMI 52	EA	EA	2.00	75%	26%	64.0	64.5 (9.3)	78.9 (65.1, 91.0)	5,759	1,473	938
	EAS	EAS	3.00	83%	34%	65.0	64.7 (9.0)	84.3 (70.1, 92.1)	235	NA	NA
	SA	SA	1.00	79%	34%	62.0	61.0 (11.1)	76.9 (62.4, 92.9)	207	NA	NA
STABILITY	EA	EA	3.00	82%	37%	65.0	64.7 (9.1)	73.4 (61.2, 85.6)	7,687	2,821	1,677
	EAS	EAS	3.00	78%	43%	65.0	64 (9.1)	83.1 (68.1, 92.8)	523	222	NA
	SA	SA	3.00	84%	41%	59.0	58.5 (10.4)	78.5 (64.7, 90.6)	469	NA	NA
ULSAM		EA	6.75	100%	10%	71.0	71.0 (0.6)	57.7 (51.7, 63.9)	686	NA	424
Vanderbilt	660	EA	8.81	45%	4%	54.8	54.0 (15.8)	86.5 (71.1, 100.4)	1,429	NA	146
	AA1M	AA	9.76	34%	6%	47.5	47.6 (16.2)	100.3 (79.6, 119.3)	755	NA	NA
	Omni1	EA	8.97	53%	3%	55.4	54.0 (15.8)	88.2 (70.1, 102.8)	1,859	NA	244
	Omni5	EA	4.32	55%	8%	55.9	52.9 (17.2)	88.2 (69.4, 103.6)	508	NA	NA
YFS		EA	6.00	46%	0%	33.0	31.6 (5.0)	109.7 (100.2, 116.4)	1,683	NA	NA
AugUR		EA	2.40	55%	22%	76.7	77.6 (5.0)	68.9 (59.0, 80.3)	677	147	184
HUNT		EA	21.20	45%	5%	44.5	45.1 (13.7)	104.0 (92.7, 114.2)	46,328	2,235	502
MGI		EA	6.00	46%	39%	52.0	50.4 (15.5)	92.8 (77.7,105.6)	20,077	3,254	1,867
UKBB		EA	4.00	50%	4%	58.0	57.1 (7.3)	92.6 (83.1,99.0)	15,442	542	241

AA: African American ancestry; EA: European ancestry; HIS: Hispanics; SA: South Asian ancestry; EAS: East Asian ancestry
CKD=Chronic Kidney Disease: eGFRcrea at baseline < 60 mL/min/1.73m²

Supplementary Table S4: The 12 identified variants for eGFR-decline were associated with other kidney phenotypes, but not with DM-status. For the 12 identified variants, we show association results for eGFR based in cystatin C[S19](“eGFRcys”, n up to 460,826), blood urea nitrogen[S12] (“BUN”, n up to 416,178), urine albumin-to-creatinine ratio[S20] (“UACR”, n up to 564,257), chronic kidney disease[S12] (“CKD”, n up to 625,219) and DM[S21] (n up to 898,130) from published GWAS. Coded allele is the faster-decline allele (which is always the eGFR-lowering allele). Genome-wide significant P-values (P<5.00x10^{-8}) are stated in bold.

			eGFRcys		BUN		UACR		CKD		DM	
**SNPID**	**Locus Name**	**EA/ OA**	**Beta**	**P**	**Beta**	**P**	**Beta**	**P**	**OR**	**P**	**OR**	**P**
Variants with genuine association for eGFR-decline												
rs34882080	*UMOD-PDILT*	a/g	-0.011	**3.44x10^{-75}**	0.010	**4.56x10^{-20}**	-0.011	1.14x10^{-05}	1.205	**3.89x10^{-56}**	0.992	0.570
rs77924615	*UMOD-PDILT*	g/a	-0.012	**6.29x10^{-94}**	0.012	**3.71x10^{-42}**	-0.010	7.24x10^{-05}	1.232	**6.66x10^{-86}**	0.989	0.400
rs10254101	*PRKAG2*	t/c	-0.0090	**1.64x10^{-70}**	0.013	**4.52x10^{-43}**	-0.0029	0.191	1.107	**1.21x10^{-25}**	0.986	0.220
rs1028455	*SPATA7*	t/a	-0.0016	9.51x10^{-04}	0.0012	0.105	0.0026	0.213	1.028	7.65x10^{-04}	0.984	0.160
rs1458038	*FGF5*	c/t	-0.0029	**9.45x10^{-09}**	0.0043	**5.99x10^{-09}**	-0.0029	0.182	1.065	**7.36x10^{-14}**	0.978	0.047
rs4930319	*OVOL1*	c/g	-0.0055	**1.26x10^{-29}**	-0.0050	**3.74x10^{-11}**	-0.0038	0.066	1.060	**7.35x10^{-12}**	1.005	0.640
rs434215	*TPPP*	a/g	-0.0044	**3.10x10^{-14}**	0.0034	0.008	0.0034	0.201	1.043	1.28x10^{-03}	0.989	0.380
rs28857283	*C15ORF54*	g/a	-0.0022	5.76x10^{-06}	0.0026	5.20x10^{-04}	-0.0025	0.210	1.050	**1.19x10^{-08}**	0.986	0.200
rs13095391	*ACVR2B*	a/c	0.00020	0.662	0.0006	0.479	-0.0017	0.743	1.022	0.011	0.983	0.180
Variants without genuine association for eGFR-decline												
rs9998485	*SHROOM3*	a/g	-0.0090	**3.94x10^{-83}**	0.0031	7.68x10^{-04}	-0.012	0.023	1.052	**4.48x10^{-08}**	1.000	0.980
rs1047891	*CPS1*	a/c	0.0039	**3.40x10^{-15}**	-0.0068	**1.26x10^{-15}**	-0.019	**4.01x10^{-18}**	1.053	**2.99x10^{-08}**	0.983	0.130
rs2453533	*GATM*	a/c	-1.00x10^{-04}	0.844	1.00x10^{-04}	0.855	-0.013	**4.49x10^{-10}**	1.076	**8.57x10^{-17}**	0.972	0.010

SNPID=Variant identifier on GRCh37, **Locus name**=Nearest Gene, **EA/OA**=Effect allele / other allele, **Beta** and **P**=genetic effect coefficient of association and association P-value, **OR**=odds ratio, **P**=association P-value.

Supplementary Table S5: **The 12 identified variants for eGFR-decline do not show heterogeneity between ancestries and FHS is not an influential study**. We conducted MR-regression to test for heterogeneity between ancestries[S13] and the meta-analyses restricted to European or African American individuals (n=325,840 and 9,038, respectively; sample sizes for other ancestries were small). We also conducted a sensitivity meta-analysis excluding the FHS study (due to an initial uncertainty in the median eGFR-decline, n=2,925) and explored direction-consistency of genetic effects in FHS alone. Shown are the P-values for between-ancestry heterogeneity (P-anc-het) and beta-estimates in mL/min/1.73m² as well as P-values for the sensitivity analyses; significant P-values ($P_{decline} \leq 0.05/12 = 4.17 \times 10^{-3}$) are stated in bold. Among the 12 variants, there was no evidence for between-ancestry heterogeneity (P-anc-het≥0.05). Association estimates excluding FHS were similar to the original analysis estimates (**Table 1**) and FHS-specific estimates were mostly directionally consistent.

				European		African American		All no FHS		FHS	
**SNPID**	**Locus Name**	**EA/ OA**	**P-anc-het**	**Beta**	**P**	**Beta**	**P**	**Beta**	**P**	**Beta**	**P**
Variants identified with genuine association for eGFR-decline											
rs34882080	*UMOD-PDILT*	a/g	0.06	0.066	**2.36×10^{-31}**	-0.083	0.174	0.065	**9.70×10^{-30}**	0.091	0.112
rs77924615	*UMOD-PDILT*	g/a	0.85	0.074	**5.50×10^{-37}**	0.016	0.836	0.073	**3.77×10^{-37}**	0.16	0.0423
rs10254101	*PRKAG2*	t/c	0.16	0.020	**7.03×10^{-05}**	0.066	0.223	0.020	**4.35×10^{-05}**	0.019	0.710
rs1028455	*SPATA7*	t/a	0.90	0.020	**1.63×10^{-05}**	0.023	0.517	0.020	**1.12×10^{-05}**	0.076	0.124
rs1458038	*FGF5*	c/t	0.23	0.019	**3.79×10^{-05}**	-0.074	0.257	0.020	**3.03×10^{-05}**	-0.030	0.565
rs4930319	*OVOL1*	c/g	0.70	0.014	**2.19×10^{-03}**	0.043	0.426	0.015	**1.37×10^{-03}**	0.045	0.347
rs434215	*TPPP*[§]	a/g	0.33	0.021	**3.80×10^{-04}**	-0.044	0.532	0.021	**5.43×10^{-04}**	0.12	0.119
rs28857283	*C15ORF54*	g/a	0.22	0.021	**3.44×10^{-06}**	0.075	0.0730	0.022	**1.32×10^{-06}**	0.015	0.745
rs13095391	*ACVR2B*	a/c	0.29	0.018	**1.67×10^{-04}**	0.062	0.207	0.017	**1.77×10^{-04}**	NA	NA
Variants without genuine association for eGFR-decline											
rs9998485	*SHROOM3*	a/g	0.65	0.0048	0.242	0.049	0.222	0.0070	0.156	NA	NA
rs1047891	*CPS1*	a/c	0.35	0.0053	0.287	-0.0040	0.930	0.0040	0.482	0.037	0.456
rs2453533	*GATM*	a/c	0.69	0.0045	0.638	0.022	0.651	0.0010	0.785	0.043	0.360

SNPID=Variant identifier on GRCh37, **Locus name**=Nearest Gene, **EA/OA**=Effect allele / other allele, **P-anc-het**=P-value of the test for between ancestry heterogeneity, **beta** and **P**=genetic effect coefficient of association and association P-value. [§] Since the *TPPP* locus lead variant had imputation quality <0.6 in 45% of the studies (median 0.64), we analyzed this locus omitting the imputation quality filter in all studies.

Supplementary Table S6: No influence by DM-adjustment versus no DM-adjustment or by model-based versus formula-based adjustment for baseline eGFR (eGFR-BL) on the 12 variants' association with eGFR-decline. We conducted a validation meta-analysis for the 12 identified variants for eGFR-decline (total n=103,970) to compare models with different covariate adjustment. Shown are beta-estimates and P-values for eGFR-decline DM-adjusted versus DM-unadjusted, and adjusted for eGFR-baseline by model as well as by formula (**Supplementary Note S1**); all models are age- and sex-adjusted. We found no impact by DM-adjustment, but by adjustment for eGFR-BL (when compared to "not adjusted for DM", which is unadjusted for eGFR-BL). For adjustment for eGFR-BL, we found the same association statistics when model-computed versus formula-derived.

			**Adjusted for DM**		**Not adjusted for DM**		**Adjusted for eGFR-BL by model**		**Adjusted for eGFR-BL by formula**	
**SNPID**	**EA/OA**	**EAF**	**beta**	**P**	**beta**	**P**	**beta**	**P**	**beta**	**P**
Variants identified with genuine association for eGFR-decline										
rs34882080	a/g	0.83	0.058	4.86×10^{-15}	0.058	4.60×10^{-15}	0.077	2.40×10^{-27}	0.078	1.06×10^{-28}
rs77924615	g/a	0.19	0.066	1.68×10^{-19}	0.066	1.34×10^{-19}	0.088	7.83×10^{-37}	0.087	4.73×10^{-37}
rs10254101	t/c	0.28	0.016	0.0130	0.016	0.0125	0.032	1.17×10^{-07}	0.031	1.15×10^{-07}
rs1028455	t/a	0.34	0.020	8.23×10^{-04}	0.020	7.76×10^{-04}	0.021	1.87×10^{-04}	0.021	1.99×10^{-04}
rs1458038	c/t	0.35	0.019	1.67×10^{-03}	0.019	1.68×10^{-03}	0.025	1.04×10^{-05}	0.024	1.31×10^{-05}
rs4930319	c/g	0.34	0.013	0.0241	0.013	0.0279	0.025	1.09×10^{-05}	0.025	5.81×10^{-06}
rs434215	a/g	0.33	0.015	0.0395	0.015	0.0414	0.027	9.20×10^{-05}	0.027	8.72×10^{-05}
rs28857283	g/a	0.37	0.019	1.08×10^{-03}	0.019	1.08×10^{-03}	0.026	2.29×10^{-06}	0.026	2.73×10^{-06}
rs13095391	a/c	0.59	0.022	1.56×10^{-04}	0.022	1.26×10^{-04}	0.027	5.70×10^{-07}	0.027	6.41×10^{-07}
Variants without genuine association for eGFR-decline										
rs9998485	a/g	0.53	0.016	6.67×10^{-03}	0.015	7.05×10^{-03}	0.030	4.55×10^{-08}	0.030	1.78×10^{-08}
rs1047891	a/c	0.31	0.010	0.1232	0.010	0.126	0.031	1.66×10^{-07}	0.030	2.53×10^{-07}
rs2453533	a/c	0.40	-0.0027	0.6378	-0.0028	0.631	0.025	4.46×10^{-06}	0.025	2.78×10^{-06}

SNPID=Variant identifier on GRCh37, **EA/OA**=Effect allele / other allele, **EAF**=Effect Allele Frequency, **beta** and **P**=genetic effect coefficient of association and association P-value.

Supplementary Table S7: Associations of *APOL1* risk variants with eGFR-decline in African American and European ancestry. While our data was derived primarily from persons of European ancestry, we explored variants in the *APOL1* gene due to previous reports for chronic kidney disease progression in 8,500 African American individuals[S22]. We conducted GWAS with the additive model for eGFR-decline unadjusted for eGFR-baseline restricted to African Americans (n up to 9,038) or to European ancestry (n up to 325,840). Shown are beta-estimates (in mL/min/1.73m²), standard errors (SE) and P-values. From 6 previously reported *APOL1* risk variants (the 7th, indel rs71785313, not analyzable here), none was associated with eGFR-decline in African American ancestry (P≥0.05). Interestingly, we detected two yet unreported SNPs near/in *APOL1* suggestively associated with eGFR-decline with P=2.8x10^{-05} and 3.10x10^{-05} in African Americans (effect allele frequency=0.01; monomorphic in European), uncorrelated with the previously reported variants (r^2<0.01).

			African American			European		
**SNPID**	**EA/OA**	**EAF**	**Beta**	**SE**	**P**	**Beta**	**SE**	**P**
rs73885319	a/g	0.77	0.001	0.05	0.98	NA	NA	NA
rs60910145	t/g	0.78	0.002	0.05	0.97	NA	NA	NA
rs4821480	t/g	0.37	-0.011	0.04	0.78	-0.015	0.0142	0.28
rs2032487	t/c	0.37	-0.004	0.04	0.91	-0.010	0.0131	0.45
rs4821481	t/c	0.37	-0.007	0.04	0.85	-0.001	0.0131	0.45
rs3752462	t/c	0.73	0.027	0.04	0.51	-0.006	0.0047	0.24
rs114021047	a/g	0.01	1.034	0.25	2.80x10^{-05}	NA	NA	NA
rs115045136	t/c	0.01	1.027	0.25	3.10x10^{-05}	NA	NA	NA

SNPID=Variant identifier on GRCh37, **EA/OA**=Effect allele / other allele, **EAF**=Effect Allele Frequency, **beta, SE** and **P**=genetic effect coefficient, standard error of association and association P-value.

SUPPLEMENTARY REFERENCES

S1. Huffman JE. Examining the current standards for genetic discovery and replication in the era of mega-biobanks. *Nat Commun*. 2018;9(1):5054.

S2. Skol AD, Scott LJ, Abecasis GR, Boehnke M. Joint analysis is more efficient than replication-based analysis for two-stage genome-wide association studies. *Nat Genet*. 2006;38(2):209-213.

S3. Coresh J, Turin TC, Matsushita K, et al. Decline in estimated glomerular filtration rate and subsequent risk of end-stage renal disease and mortality. *JAMA - J Am Med Assoc*. 2014;311(24):2518-2531.

S4. Levey AS, Stevens LA, Schmid CH, et al. A new equation to estimate glomerular filtration rate. *Ann Intern Med*. 2009;150(9):604-612.

S5. The 1000 Genomes Project Consortium. An integrated map of genetic variation Supplementary Material. *Nature*. 2012;135:1-113.

S6. McCarthy S, Das S, Kretzschmar W, et al. A reference panel of 64,976 haplotypes for genotype imputation. *Nat Genet*. 2016;48(10):1279-1283.

S7. Howie BN, Donnelly P, Marchini J. A flexible and accurate genotype imputation method for the next generation of genome-wide association studies. *PLoS Genet*. 2009;5(6):e1000529.

S8. Fuchsberger C, Abecasis GR, Hinds DA. Minimac2: Faster genotype imputation. *Bioinformatics*. 2015;31(5):782-784.

S9. Fuchsberger C, Taliun D, Pramstaller PP, Pattaro C. GWAtoolbox: An R package for fast quality control and handling of genome-wide association studies meta-analysis data. *Bioinformatics*. 2012;28(3):444-445.

S10. Aschard H, Vilhjálmsson BJ, Joshi AD, Price AL, Kraft P. Adjusting for heritable covariates can bias effect estimates in genome-wide association studies. *Am J Hum Genet*. 2015;96(2):329-339.

S11. Willer CJ, Li Y, Abecasis GR. METAL: Fast and efficient meta-analysis of genomewide association scans. *Bioinformatics*. 2010;26(17):2190-2191.

S12. Wuttke M, Li Y, Li M, et al. A catalog of genetic loci associated with kidney function from analyses of a million individuals. *Nat Genet*. 2019;51(6):957-972.

S13. Mägi R, Horikoshi M, Sofer T, et al. Trans-ethnic meta-regression of genome-wide association studies accounting for ancestry increases power for discovery and improves fine-mapping resolution. *Hum Mol Genet*. 2017;26(18):3639-3650.

S14. Hastie T, Tibshirani R. Varying-Coefficient Models. *J R Stat Soc Ser B*. 1993;55(4):757-779.

S15. Wood SN. *Generalized Additive Models: An Introduction with R, Second Edition*.; 2017.

S16. Gorski M, Jung B, Li Y, et al. Meta-analysis uncovers genome-wide significant variants for rapid kidney function decline. *Kidney Int*. 2021;99(4):926-939.

S17. Wanner C, Krane V, März W, et al. Randomized controlled trial on the efficacy and safety of atorvastatin in patients with type 2 diabetes on hemodialysis (4D study): Demographic and baseline characteristics. *Kidney Blood Press Res*. 2004;27(4):259-266.

S18. Winkler TW, Günther F, Höllerer S, et al. A joint view on genetic variants for adiposity differentiates subtypes with distinct metabolic implications. *Nat Commun*. 2018;9(1):1946.

S19. Stanzick KJ, Li Y, Schlosser P, et al. Discovery and prioritization of variants and genes for kidney function in >1.2 million individuals. *Nat Commun*. 2021;12(1):4350.

S20. Teumer A, Li Y, Ghasemi S, et al. Genome-wide association meta-analyses and fine-mapping elucidate pathways influencing albuminuria. *Nat Commun*. 2019;10(1):4130.

S21. Mahajan A, Taliun D, Thurner M, et al. Fine-mapping type 2 diabetes loci to single-variant resolution using high-density imputation and islet-specific epigenome maps. *Nat Genet*. 2018;50(11):1505-1513.

S22. Parsa A, Kao WHL, Xie D, et al. *APOL1* Risk Variants, Race, and Progression of Chronic Kidney Disease. *N Engl J Med*. 2013;369(23):2183-2196.

Extended acknowledgements, study funding information and author contributions

The Deutsche Forschungsgemeinschaft (DFG, German Research Foundation) supported the meta-analysis – Project-ID 387509280 – SFB1350 (Subproject C6 to I.M.H.).

ADVANCE ADVANCE genomic sub-studies were supported by grants from the Ministry of Science and Innovation from the Quebec Government, from Genome Quebec, from the Consortium Québécois du Médicament, from the Canadian Institutes of Health Research and from Medpharmgene, OPTITHERA Inc and Les Laboratoires Servier.

AFTER EU The AFTER EU study is the Danish part of the EURAGEDIC study which was supported by the European Commission (contract QLG2-CT-2001– 01669). The genotyping for this study was part of the Genetics of Diabetic Nephropathy (GenDN) study, primarily funded by Juvenile Diabetes Research Foundation (JDRF) International Prime Award Number 17-2013- 8. Tarunveer S Ahluwalia was also funded by the GenDN study grant and Lundbeck foundation Travel Grant (Ref. Number 2013-14471).

AMISH We thank the Amish research volunteers for their long-standing partnership in research, and the research staff at the Amish Research Clinic for their work and dedication. The Amish contribution was supported by NIH grants R01 AG18728, R01 HL088119, U01 GM074518, U01 HL072515, U01 HL084756, and NIH K12RR023250, and P30 DK072488. Additional support was provided by the University of Maryland General Clinical Research Center, grant M01 RR 16500, the Baltimore Veterans Administration Medical Center Geriatrics Research, and the Paul Beeson Physician Faculty Scholars in Aging Program

ARIC The Atherosclerosis Risk in Communities study has been funded in whole or in part with Federal funds from the National Heart, Lung, and Blood Institute, National Institutes of Health, Department of Health and Human Services (contract numbers HHSN268201700001I, HHSN268201700002I, HHSN268201700003I, HHSN268201700004I and HHSN268201700005I), R01HL087641, R01HL086694; National Human Genome Research Institute contract U01HG004402; and National Institutes of Health contract HHSN268200625226C. The authors thank the staff and participants of the ARIC study for their important contributions. Infrastructure was partly supported by Grant Number UL1RR025005, a component of the National Institutes of Health and NIH Roadmap for Medical Research. The work of Anna Köttgen was supported by the Deutsche Forschungsgemeinschaft (DFG, German Research Foundation) – Project-ID 431984000 – SFB 1453, and a DFG Heisenberg Professorship (KO 3598/5-1).

ASPS The authors thank the staff and the participants for their valuable contributions. We thank Birgit Reinhart for her long-term administrative commitment, Elfi Hofer for the technical assistance at creating the DNA bank, Ing. Johann Semmler and Anita Harb for DNA sequencing and DNA analyses by TaqMan assays and Irmgard Poelzl for supervising the quality management processes after ISO9001 at the biobanking and

DNA analyses. The research reported in this article was funded by the Austrian Science Fond (FWF) grant number P20545-P05, P13180 and PI904 as well as by the Austrian National Bank (OeNB) Anniversary Fund grant number P15435 and the Austrian Federal Ministry of Science, Research and Economy under the aegis of the EU Joint Programme Neurodegenerative Disease Research (JPND)-www.jpnd.eu. The Medical University of Graz supports the databank of the ASPS.

AugUR Age-related diseases: Understanding Genetic and non-genetic influences - a study at the University of Regensburg. Cohort recruiting and management was funded by the Federal Ministry of Education and Research (BMBF-01ER1206, BMBF-01ER1507, to Iris M. Heid) and by the German Research Foundation (DFG HE 3690/7-1, to Iris M. Heid). Genome-wide genotyping and lipid concentrations phenotyping was funded by the University of Regensburg for the Department of Genetic Epidemiology. The computational work supervised by Iris M. Heid was funded by the German Research Foundation (DFG) – Project-ID 387509280 – SFB 1350 (to Iris M. Heid).

BioMe The Mount Sinai IPM Biobank Program is supported by The Andrea and Charles Bronfman Philanthropies. Ruth Loos is funded by R01DK110113, U01HG007417, R01DK101855, and R01DK107786.

CHS Cardiovascular Health Study: This research was supported by contracts HHSN268201200036C, HHSN268200800007C, HHSN268201800001C, N01HC55222, N01HC85079, N01HC85080, N01HC85081, N01HC85082, N01HC85083, N01HC85086, 75N92021D00006, and grants U01HL080295 and U01HL130114 from the National Heart, Lung, and Blood Institute (NHLBI), with additional contribution from the National Institute of Neurological Disorders and Stroke (NINDS). Additional support was provided by R01AG023629 from the National Institute on Aging (NIA). A full list of principal CHS investigators and institutions can be found at CHS-NHLBI.org. A The provision of genotyping data was supported in part by the National Center for Advancing Translational Sciences, CTSI grant UL1TR001881, and the National Institute of Diabetes and Digestive and Kidney Disease Diabetes Research Center (DRC) grant DK063491 to the Southern California Diabetes Endocrinology Research Center. The content is solely the responsibility of the authors and does not necessarily represent the official views of the National Institutes of Health.

CILENTO This study was supported by grants from the Italian Ministry of Universities (PON03PE_00060_7, IDF SHARID ARS01_01270), and the Assessorato Ricerca Regione Campania (POR CAMPANIA 2000/2006 MISURA 3.16). We address special thanks to the populations of Campora, Gioi, and Cardile for their participation in the study. We thank dr. Debora Chirico, dr. Michelina De Cristofaro, dr. Raffaele D'Urso and dr. Giovanni D'Arena for Cilento data collection.

DECODE The study was funded by deCODE Genetics/Amgen inc. We thank the study subjects for their valuable participation and our colleagues, who contributed to data collection, sample handling, and genotyping

DIACORE Cohort recruiting and management was funded by the KfH Stiftung Präventivmedizin e.V. (Carsten A. Böger). Genome-wide genotyping was funded the Else Kröner-Fresenius-Stiftung (2012_A147), the KfH Stiftung Präventivmedizin and the University Hospital Regensburg. The Deutsche Forschungsgemeinschaft (DFG, German Research Foundation) supported this work – Project-ID 387509280 – SFB 1350 (Subproject C6 to I.M.H.) and Iris Heid and Carsten Böger received funding by DFG BO 3815/4-1. This project has received funding from the Innovative Medicines Initiative 2 Joint Undertaking under grant agreement No 115974. This Joint Undertaking receives support from the European Union's Horizon 2020 research and innovation programme and EFPIA with JDRF (BEAT-DKD).

ESTHER The ESTHER study was funded by the Saarland state Ministry for Social Affairs, Health, Women and Family Affairs (Saarbrücken, Germany), the Baden-Württemberg state Ministry of Science, Research and Arts (Stuttgart, Germany), the Federal Ministry of Education and Research (Berlin, Germany) and the Federal Ministry of Family Affairs, Senior Citizens, Women and Youth (Berlin, Germany).

FHS The Framingham Heart Study is supported by HHSN268201500001.

FINCAVAS The Finnish Cardiovascular Study (FINCAVAS) has been financially supported by the Competitive Research Funding of the Tampere University Hospital (Grant 9M048 and 9N035), the Finnish Cultural Foundation, the Finnish Foundation for Cardiovascular Research, the Emil Aaltonen Foundation, Finland, the Tampere Tuberculosis Foundation, EU Horizon 2020 (grant 755320 for TAXINOMISIS; grant 848146 for To_Aition), and the Academy of Finland grant 322098. The authors thank the staff of the Department of Clinical Physiology for collecting the exercise test data.

GCKD The GCKD study was funded by the German Ministry of Research and Education (Bundesministerium für Bildung und Forschung, BMBF), by the Foundation KfH Stiftung Präventivmedizin. Unregistered grants to support the study were provided by Bayer, Fresenius Medical Care and Amgen. Genotyping was supported by Bayer Pharma AG. The work of Matthias Wuttke was supported by the Deutsche Forschungsgemeinschaft (DFG, German Research Foundation) Project-ID 431984000 – SFB 1453. The work of Yong Li was supported by DFG KO 3598/4-2.

GEISINGER We would like to acknowledge the participants, staff, and our colleagues associated with the Geisinger MyCode Community Health Initiative. We also thank the staff of the PACDC of Geisinger for assistance with the phenotypic data, and the staff of the Biomedical & Translational Informatics and Kidney Health Research Institute

HANDLS The authors thank all study participants and the Healthy Aging in Neighborhoods of Diversity across the Life Span (HANDLS) study medical staff for their contributions. The HANDLS study was supported by the Intramural Research Program of the NIH, National Institute on Aging (project # Z01-AG000513 and human subjects protocol number 09-AG-N248). Data analyses for the HANDLS study utilized the high-performance computational resources of the Biowulf Linux cluster at the National Institutes of Health, Bethesda, MD. (http://biowulf.nih.gov; http://hpc.nih.gov).

HUNT The Trøndelag Health Study (The HUNT Study) is a collaboration between HUNT Research Centre (Faculty of Medicine and Health Sciences, NTNU, Norwegian University of Science and Technology), Trøndelag County Council, Central Norway Regional Health Authority, and the Norwegian Institute of Public Health. The HUNT study was approved by the Central Norway Regional Committee for Medical and Health Research Ethics (REC Central no. 2015/1188), and written informed consent was given by all participants.

HYPERGENES Funding Source: HYPERGENES project (FP7-HEALTH-F4-2007-201550) and InterOmics (PB05 MIUR-CNR Italian Flagship Project).

JHS The Jackson Heart Study (JHS) is supported and conducted in collaboration with Jackson State University (HHSN268201800013I), Tougaloo College (HHSN268201800014I), the Mississippi State Department of Health (HHSN268201800015I/HHSN26800001) and the University of Mississippi Medical Center (HHSN268201800010I, HHSN268201800011I and HHSN268201800012I) contracts from the National Heart, Lung, and Blood Institute (NHLBI) and the National Institute for Minority Health and Health Disparities (NIMHD). The authors also wish to thank the staffs and participants of the JHS. The views expressed in this manuscript are those of the authors and do not necessarily represent the views of the National Heart, Lung, and Blood Institute; the National Institutes of Health; or the U.S. Department of Health and Human Services. Laura M. Raffield is supported by

T32 HL129982 and the National Center for Advancing Translational Sciences, National Institutes of Health, through Grant KL2TR002490.

J-MICC We thank Dr. Nobuyuki Hamajima for his work for initiating and organizing the J-MICC Study as the former principal investigator. This study was supported by Grants-in-Aid for Scientific Research for Priority Areas of Cancer (No. 17015018) and Innovative Areas (No. 221S0001) and by the Japan Society for the Promotion of Science (JSPS) KAKENHI Grant (No. 16H06277 [CoBiA]) from the Japanese Ministry of Education, Culture, Sports, Science and Technology. This work was also supported in part by funding for the BioBank Japan Project from the Japan Agency for Medical Research and Development since April 2015, and the Ministry of Education, Culture, Sports, Science and Technology from April 2003 to March 2015.

KORA The KORA study was initiated and financed by the Helmholtz Zentrum München – German Research Center for Environmental Health, which is funded by the German Federal Ministry of Education and Research (BMBF) and by the State of Bavaria. Furthermore, KORA research was supported within the Munich Center of Health Sciences (MC-Health), Ludwig-Maximilians-Universität, as part of LMUinnovativ. Statistical KORA analyses were supported by DFG BO-3815/4-1 (to Carsten A. Böger), BMBF 01ER1206, 01ER1507 (to Iris M. Heid), by the University of Regensburg and by the Deutsche Forschungsgemeinschaft (DFG, German Research Foundation) – Project-ID 387509280 – SFB 1350 (Subproject C6 to I.M.H.) and DFG BO 3815/4-1.

LifeLines The LifeLines Cohort Study, and generation and management of GWAS genotype data for the LifeLines Cohort Study is supported by the Netherlands Organization of Scientific Research NWO (grant 175.010.2007.006), the Economic Structure Enhancing Fund (FES) of the Dutch government, the Ministry of Economic Affairs, the Ministry of Education, Culture and Science, the Ministry for Health, Welfare and Sports, the Northern Netherlands Collaboration of Provinces (SNN), the Province of Groningen, University Medical Center Groningen, the University of Groningen, Dutch Kidney Foundation and Dutch Diabetes Research Foundation. The authors wish to acknowledge the services of the Lifelines Cohort Study, the contributing research centers delivering data to Lifelines, and all the study participants.

MDC-CC This study was supported by the European Research Council (Consolidator grant nr 649021, Orho-Melander), the Swedish Research Council, the Swedish Heart and Lung Foundation, the Novo Nordic Foundation, the Swedish Diabetes Foundation, and the Påhlsson Foundation, and by equipment grants from the Knut and Alice Wallenberg Foundation, the Region Skåne, Skåne University Hospital, the Linneus Foundation for the Lund University Diabetes Center and Swedish Foundation for Strategic Research for IRC15-0067.

MESA MESA and the MESA SHARe projects are conducted and supported by the National Heart, Lung, and Blood Institute (NHLBI) in collaboration with MESA investigators. Support for MESA is provided by contracts 75N92020D00001, HHSN268201500003I, N01-HC-95159, 75N92020D00005, N01-HC-95160, 75N92020D00002, N01-HC-95161, 75N92020D00003, N01-HC-95162, 75N92020D00006, N01-HC-95163, 75N92020D00004, N01-HC-95164, 75N92020D00007, N01-HC-95165, N01-HC-95166, N01-HC-95167, N01-HC-95168, N01-HC-95169, UL1-TR-000040, UL1-TR-001079, and UL1-TR-001420. Also supported by the National Center for Advancing Translational Sciences, CTSI grant UL1TR001881, and the National Institute of Diabetes and Digestive and Kidney Disease Diabetes Research Center (DRC) grant DK063491 to the Southern California Diabetes Endocrinology Research Center.

METSIM Funding Source: HYPERGENES project (FP7-HEALTH-F4-2007-201550) and InterOmics (PB05 MIUR-CNR Italian Flagship Project).

MGI The authors acknowledge the Michigan Genomics Initiative participants, Precision Health at the University of Michigan, the University of Michigan Medical School Central Biorepository, and the University of Michigan Advanced Genomics Core for providing data and specimen storage, management, processing, and distribution services, and the Center for Statistical Genetics in the Department of Biostatistics at the School of Public Health for genotype data curation, imputation, and management in support of the research reported in this publication. Funding was provided by the NIH (R35-HL135824).

NESDA Funding was obtained from the Netherlands Organization for Scientific Research (Geestkracht program grant 10-000-1002); the Center for Medical Systems Biology (CSMB, NWO Genomics), Biobanking and Biomolecular Resources Research Infrastructure (BBMRI-NL), VU University's Institutes for Health and Care Research (EMGO+) and Neuroscience Campus Amsterdam, University Medical Center Groningen, Leiden University Medical Center, National Institutes of Health (NIH, R01D0042157-01A, MH081802, Grand Opportunity grants 1RC2 MH089951 and 1RC2 MH089995). Part of the genotyping and analyses were funded by the Genetic Association Information Network (GAIN) of the Foundation for the National Institutes of Health.Computing was supported by BiG Grid, the Dutch e-Science Grid, which is financially supported by NWO.

OGP The Ogliastra Genetic Park study was supported by grant from the Italian Ministry of Education, University and Research (MIUR) no. 5571/DSPAR/2002. We thank all study participants for their contributions and the municipal administrations for their economic and logistic support.

PIVUS The PIVUS study was supported by Wellcome Trust grants WT098017, WT064890, WT090532, Uppsala University, Uppsala University Hospital, the Swedish Research Council and the Swedish Heart-Lung Foundation. Cecilia M. Lindgren is supported by the Li K**a** Shing Foundation, NIHR Oxford Biomedical Research Centre, Oxford, NIH (1P50HD104224-01), Gates Foundation (INV-024200), and a Wellcome Trust Investigator Award (221782/Z/20/Z).

POPGEN The PopGen 2.0 network was supported by a grant from the German Ministry for Education and Research (01EY1103). Sandra Freitag-Wolf was supported by German Research Foundation, Clusters of Excellence 306, Inflammation at Interfaces.

PREVEND The Prevention of Renal and Vascular Endstage Disease Study (PREVEND) genetics is supported by the Dutch Kidney Foundation (Grant E033), the EU project grant GENECURE (FP-6 LSHM CT 2006 037697), the National Institutes of Health (grant LM010098), the Netherlands organization for health research and development (NWO VENI grant 916.761.70), and the Dutch Inter University Cardiology Institute Netherlands (ICIN). Niek Verweij was supported by NWO VENI grant 016.186.125.

RS The Rotterdam Study (RS) has been approved by the Medical Ethics Committee of the Erasmus MC (registration number MEC 02.1015) and by the Dutch Ministry of Health, Welfare and Sport (Population Screening Act WBO, license number 1071272-159521-PG). The RS has been entered into the Netherlands National Trial Register (NTR; www.trialregister.nl) and into the WHO International Clinical Trials Registry Platform (ICTRP; www.who.int/ictrp/network/primary/en/) under shared catalogue number NTR6831. All participants provided written informed consent to participate in the study and to have their information obtained from treating physicians. The generation and management of GWAS genotype data for the RS (RS I, RS II, RS III) was executed by the Human Genotyping Facility of the Genetic Laboratory of the Department of Internal Medicine, Erasmus MC, Rotterdam, the Netherlands. The GWAS datasets are supported by the Netherlands Organisation of Scientific Research NWO Investments (nr. 175.010.2005.011, 911-03-012), the

Genetic Laboratory of the Department of Internal Medicine, Erasmus MC, the Research Institute for Diseases in the Elderly (014-93-015; RIDE2), the Netherlands Genomics Initiative (NGI)/Netherlands Organisation for Scientific Research (NWO) Netherlands Consortium for Healthy Aging (NCHA), project nr. 050-060- 810. We thank Pascal Arp, Mila Jhamai, Marijn Verkerk, Lizbeth Herrera and Marjolein Peters, and Carolina Medina-Gomez, for their help in creating the GWAS database, and Karol Estrada, Yurii Aulchenko, and Carolina Medina-Gomez, for the creation and analysis of imputed data. The RS is funded by Erasmus Medical Center and Erasmus University, Rotterdam, Netherlands Organization for the Health Research and Development (ZonMw), the Research Institute for Diseases in the Elderly (RIDE), the Ministry of Education, Culture and Science, the Ministry for Health, Welfare and Sports, the European Commission (DG XII), and the Municipality of Rotterdam. The authors are grateful to the study participants, the staff from the RS and the participating general practitioners and pharmacists.

SHIP SHIP is part of the Community Medicine Research net of the University of Greifswald, Germany, which is funded by the Federal Ministry of Education and Research (grants no. 01ZZ9603, 01ZZ0103, and 01ZZ0403), the Ministry of Cultural Affairs as well as the Social Ministry of the Federal State of Mecklenburg-West Pomerania, and the network 'Greifswald Approach to Individualized Medicine (GANI_MED)' funded by the Federal Ministry of Education and Research (grant 03IS2061A). Genome-wide data have been supported by the Federal Ministry of Education and Research (grant no. 03ZIK012) and a joint grant from Siemens Healthineers, Erlangen, Germany and the Federal State of Mecklenburg- West Pomerania. The University of Greifswald is a member of the Caché Campus program of the InterSystems GmbH.

SiMES The Singapore Malay Eye Study (SiMES) was funded by the National Medical Research Council (NMRC 0796/2003 and NMRC/STaR/0003/2008) and Biomedical Research Council (BMRC, 09/1/35/19/616). The Genome Institute of Singapore provided services for genotyping.

SINDI The Singapore Indian Eye Study (SINDI) was funded by grants from the Biomedical Research Council of Singapore (BMRC 09/1/35/19/616 and 08/1/35/19/550), and the National Medical Research Council of Singapore (NMRC/STaR/0003/2008). The Genome Institute of Singapore provided services for genotyping.

SOLID-TIMI 52 The SOLID-TIMI 52 trial was supported and funded by grants from GlaxoSmithKline.

STABILITY The STABILITY trial was supported and funded by grants from GlaxoSmithKline.

UK Biobank This research has been conducted using the UK Biobank resource under application number 20272.

ULSAM The ULSAM study was supported by Wellcome Trust grants WT098017, WT064890, WT090532, Uppsala University, Uppsala University Hospital, the Swedish Research Council and the Swedish Heart-Lung Foundation. Johan Ärnlöv was supported by The Swedish Research Council, and the Swedish Heart-Lung Foundation.

Vanderbilt The data used for the analyses were obtained from Vanderbilt University Medical Center's BioVU, which is supported by numerous sources: institutional funding, private agencies, and federal grants. These include the NIH funded Shared Instrumentation Grant S10RR025141; and CTSA grants UL1TR002243, UL1TR000445, and UL1RR024975. Genomic data are also supported by investigator-led projects that include U01HG004798, R01NS032830, RC2GM092618, P50GM115305, U01HG006378, U19HL065962, R01HD074711; and additional funding sources listed at https://victr.vanderbilt.edu/pub/biovu/. Jacklyn N. Hellwege is supported by the Vanderbilt Molecular and Genetic Epidemiology of Cancer training program, funded by T32CA160056.

YFS The Young Finns Study has been financially supported by the Academy of Finland: grants 322098, 286284, 134309 (Eye), 126925, 121584, 124282, 129378 (Salve), 117787 (Gendi), and 41071 (Skidi); the Social Insurance Institution of Finland; Competitive State Research Financing of the Expert Responsibility area of Kuopio, Tampere and Turku University Hospitals (grant X51001); Juho Vainio Foundation; Paavo Nurmi Foundation; Finnish Foundation for Cardiovascular Research ; Finnish Cultural Foundation; The Sigrid Juselius Foundation; Tampere Tuberculosis Foundation; Emil Aaltonen Foundation; Yrjö Jahnsson Foundation; Signe and Ane Gyllenberg Foundation; Diabetes Research Foundation of Finnish Diabetes Association; EU Horizon 2020 (grant 755320 for TAXINOMISIS; grant 848146 for To_Aition); European Research Council (grant 742927 for MULTIEPIGEN project); Tampere University Hospital Supporting Foundation and Finnish Society of Clinical Chemistry. We thank the teams that collected data at all measurement time points; the persons who participated as both children and adults in these longitudinal studies; and biostatisticians Irina Lisinen, Johanna Ikonen, Noora Kartiosuo, Ville Aalto, and Jarno Kankaanranta for data management and statistical advice.

Meta-analysis German Research Foundation (Project-ID 387509280, SFB1350, Subproject C6 to I.M.H.), computing resources by University of Regensburg.

LifeLines group author genetics

LifeLines Cohort Study: Behrooz Z Alizadeh (1), H Marike Boezen (1), Lude Franke (2), Pim van der Harst (3), Gerjan Navis (4), Marianne Rots (5), Harold Snieder (1), Morris Swertz (2), Bruce HR Wolffenbuttel (6), Cisca Wijmenga (2)

(1) Department of Epidemiology, University of Groningen, University Medical Center Groningen, The Netherlands
(2) Department of Genetics, University of Groningen, University Medical Center Groningen, The Netherlands
(3) Department of Cardiology, University of Groningen, University Medical Center Groningen, The Netherlands
(4) Department of Internal Medicine, Division of Nephrology, University of Groningen, University Medical Center Groningen, The Netherlands
(5) Department of Pathology and Medical Biology, University of Groningen, University Medical Center Groningen, The Netherlands
(6) Department of Endocrinology, University of Groningen, University Medical Center Groningen, The Netherlands

Author contributions

MG, HR, AT, PM, CAB, TB, CP, AK, FK and IMH **wrote the manuscript**. MG, HR, AT, MWu, CAB, AK, and CP **designed the study**. GS, MSc, BOT, TSA, JÄ, SJLB, BBa, BBr,GB, MB, EB, HB, RJC, JChal, CC, MCi, JCo, MHdB, KEc, ME, CF, RTG, VG, CG, DG, SHa, PH, AHi, KeH, BH, HH, KHv, MAI, BJ, MKä, CK, WKo, HKr, BKK, JK, MLa, TL, LL, RJFL, MAL, OM, YM, AMo, GNN, MNai, MLO, MO, AP, SAP, BWJHP, MP, BMP, OTR, RRe, MR, PR, CSa, HSc, RS, BS, MSi, HSn, KStar, KStef, HSto, KStr, HStr, PS, PvdH, UV, KW, LWal, DMW, HW, CW, TWo, MW, QY, MZ, UT, CAB, AK, FK, CP **managed an individual contributing study**. MG, HR, AT, SGr, FG, MWu, TW, YL, GS, JChai, AC, MCo, MF, AHo, KHo, MLi, MSc, BOT, ATi, JW, TSA, PA, MLBig, GB, RJC, JFC, JChal, MLiC, JC, SF, MGh, SGh, DG, PH, EH, SHw, NSJ, BJ, IK, CK, HKr, BK, LAL, LLy, AMa, PPM, NM, AMo, MNak, MN, BN, IMN, TN, JO, AP, SAP, MHP, LMR, MR, KMR, DR, KR, CSc, SSe, KBS, XS, KStan, KStar, SSz, CHLT, LT, JTr, PvdH, PJvdM, NV, MW, QY, LMY, CAB, AK, CP and IMH **performed statistical methods**

and analysis. MG, HR, SGr, MWu, TW, YL, GS, AC, MCo, AHo, KHo, MLi, MSc, JW, TSA, PA, RJC, FD, AF, PG, SGh, DG, PH, EH, NSJ, CK, LLy, YM, PPM, MNak, TN, JO, AP, SAP, MHP, KR, CSc, SSe, CMS, KBS, KStan, KStar, SSz, LT, JTr, PJvdM, SW, LMY, CAB and IMH **performed bioinformatics**. MG, LWal, HW, MW, LMY, UT, CAB, AK, CP and IMH **interpreted results**. AT, MF, JÄ, EB, CC, JC, AF, RTG, VG, PH, KHv, MKä, CK, WKo, BKK, MLa, LAL, TL, LL, LLy, TM, OM, YM, NM, AMo, JcM, MO, BWJHP, MHP, OTR, JIR, MSi, KStar, KDT, JTr, SV, PvdH, UV, KW, MWa, CAB and FK **performed genotyping**. MG, HR, AT, SGr, PM, FG, MWu, TW, YL, TB, GS, JChai, AC, MCo, MF, AHo, KHo, MLi, MSc, BOT, ATi, JW, TSA, PA, JÄ, BOA, SJLB, BBa, BBr,NB, MLBig, GB, MB, EB, EPB, HB, RJC, JFC, LC, JChal, MLiC, MLingC, CC, MCi, JC, JCo, DC, MHdB, FD, KEc, KEn, ME, AF, SF, CF, PG, RTG, MGh, SGh, VG, CG, DG, SHa, PH, AHi, KeH, EH, BH, HH, NH, KHv, SHw, MAI, NSJ, BJ, MKä, IK, CK, WKo, HKr, BKK, BK, JK, MLa, LAL, TL, WL, Lcs, LL, CL, RJFL, MAL, LLy, AMa, CM, TM, OM, YM, PPM, NM, AMo, JcM, GNN, MNai, MNak, MNal, MN, KN, BN, IMN, TN, MLO, JO, IO, MO, AP, SAP, BWJHP, MP, MHP, BMP, LMR, OTR, RRe, MR, KMR, FR, ARR, PR, JIR, DR, KR, CSa, ES, HSc, RS, BS, CSc, SSe, CMS, KBS, XS, MSi, HSn, KStan, KStar, KStef, HSto, KStr, HStr, PS, SSz, KDT, CHLT, LT, JTr, SV, PvdH, PJvdM, NV, UV, KW, MWa, LWal, SW, DMW, HW, CW, TWo, MW, QY, LMY, MZ, AZ, UT, CAB, AK, FK, CP and IMH **critically reviewed the manuscript**. EPB, HB, JChal, MLingC, CC, MCi, JCo, KEc, RTG, VG, PH, AHi, HH, NH, KHv, BJ, MKä, BKK, MLa, TL, WL, LL, CM, MNai, KN, MLO, IO, SAP, BWJHP, OTR, MR, ARR, PR, RS, MSi, KStar, KStef, SV, KW, LWal, HW, TWo, MW, MZ, UT, CAB, AK and CP **recruited subjects**.
